# Shuffling the yeast genome using CRISPR/Cas9-generated DSBs that target the transposable Ty1 elements

**DOI:** 10.1371/journal.pgen.1010590

**Published:** 2023-01-26

**Authors:** Lei Qi, Yang Sui, Xing-Xing Tang, Ryan J. McGinty, Xiao-Zhuan Liang, Margaret Dominska, Ke Zhang, Sergei M. Mirkin, Dao-Qiong Zheng, Thomas D. Petes

**Affiliations:** 1 Ocean College, Zhejiang University, Zhoushan, China; 2 Department of Molecular Genetics and Microbiology, Duke University, Durham, North Carolina, United States of America; 3 Department of Biology, Tufts University, Medford, Massachusetts, United States of America; 4 Department of Biomedical Informatics, Harvard Medical School, Boston, Massachusetts, United States of America; 5 College of Life Science, Zhejiang University, Hangzhou, China; Sorbonne Universite UFR de Chimie, FRANCE

## Abstract

Although homologous recombination between transposable elements can drive genomic evolution in yeast by facilitating chromosomal rearrangements, the details of the underlying mechanisms are not fully clarified. In the genome of the yeast *Saccharomyces cerevisiae*, the most common class of transposon is the retrotransposon Ty1. Here, we explored how Cas9-induced double-strand breaks (DSBs) directed to Ty1 elements produce genomic alterations in this yeast species. Following Cas9 induction, we observed a significant elevation of chromosome rearrangements such as deletions, duplications and translocations. In addition, we found elevated rates of mitotic recombination, resulting in loss of heterozygosity. Using Southern analysis coupled with short- and long-read DNA sequencing, we revealed important features of recombination induced in retrotransposons. Almost all of the chromosomal rearrangements reflect the repair of DSBs at Ty1 elements by non-allelic homologous recombination; clustered Ty elements were hotspots for chromosome rearrangements. In contrast, a large proportion (about three-fourths) of the allelic mitotic recombination events have breakpoints in unique sequences. Our analysis suggests that some of the latter events reflect extensive processing of the broken ends produced in the Ty element that extend into unique sequences resulting in break-induced replication. Finally, we found that haploid and diploid strain have different preferences for the pathways used to repair double-stranded DNA breaks. Our findings demonstrate the importance of DNA lesions in retrotransposons in driving genome evolution.

## Author summary

Transposable elements can affect gene expression and gene function. Recombination between elements located on the same chromosome can produce deletions, duplications, and inversions, whereas recombination between transposable elements on non-homologous chromosomes can generate translocations. Thus, recombination between transposable elements is an important cause of genetic variation. In this report, we present a comprehensive analysis of recombination events induced in both haploid and diploid strains of *Saccharomyces cerevisiae* by targeting the Ty1 family of retrotransposons with CRISPR/Cas9. Chromosome rearrangements were stimulated more than 1000-fold. DNA sequencing, using both conventional short “reads” and ultra-long “reads”, showed that almost all of these rearrangements had Ty1 elements at their breakpoints. In contrast, we also observed a stimulation of allelic mitotic recombination events between homologs, most of which did not involve direct Ty-Ty interactions. Our results provided novel insights into the mechanism of genome evolution as regulated by repetitive sequences.

## Introduction

### Transposable yeast (Ty) elements: general considerations

In the yeast *Saccharomyces cerevisiae*, there are about 50 retrotransposons (termed Ty elements), although the number of such elements shows considerable strain-to-strain variation [1]. Each 6-kb element contains sequences necessary for its expression and transposition [2]. The most common class of retrotransposon (Ty1) is flanked by 330-bp long terminal repeats (LTRs) called delta elements [2]. The Ty2 element is closely related to Ty1 in its DNA sequences and is also flanked by delta elements [3]. There are 32 intact Ty1 elements in the S288c genome (the reference genome in the Saccharomyces Genome Database) and two truncated Ty1 elements [1, 3].

### Ty elements as agents of genomic alterations

**Fig 1:**
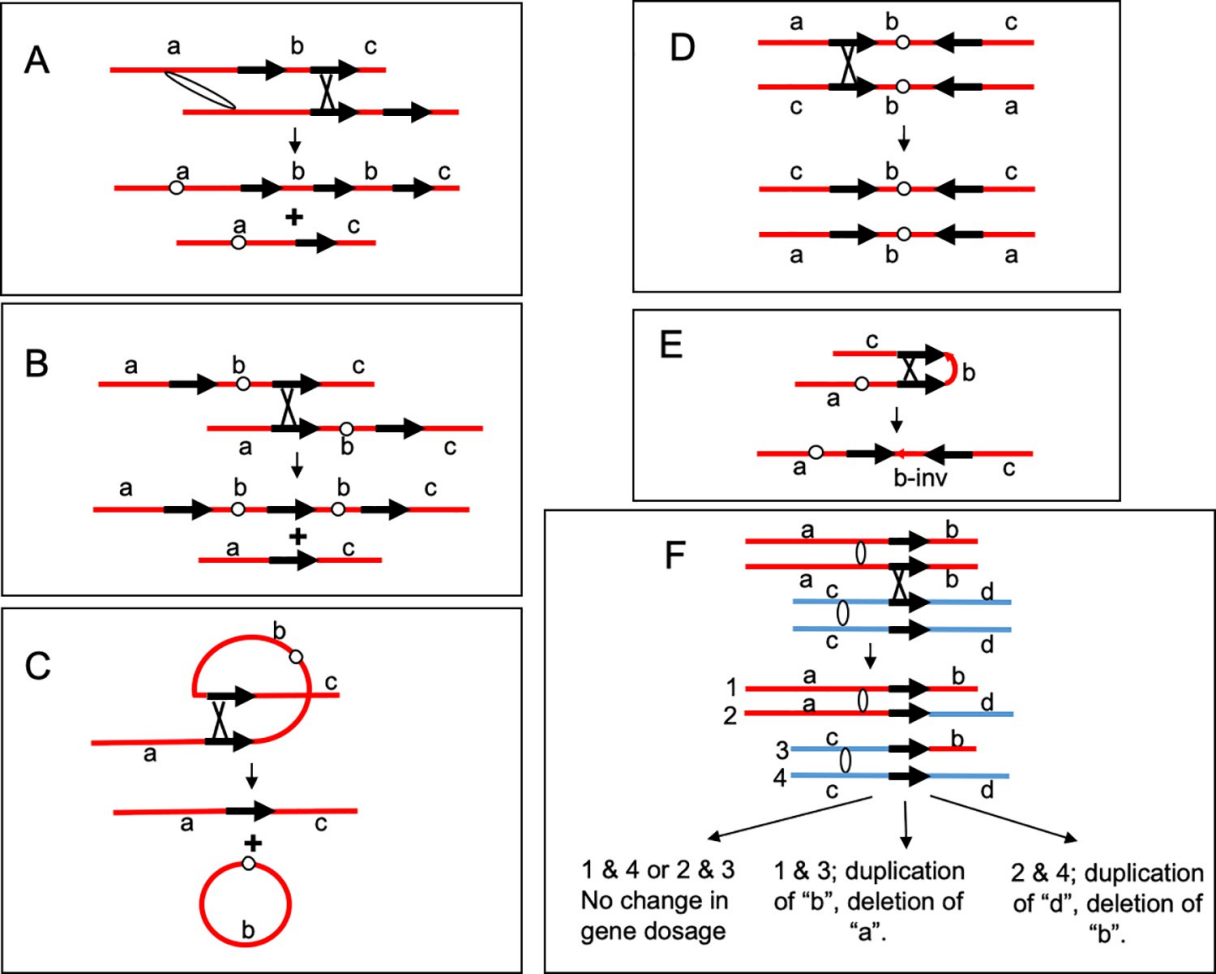
Ectopic recombination between Ty elements resulting in chromosome rearrangements. Ty elements are depicted with black arrows, and centromeres are shown as white ovals or circles. **(A)** An unequal crossover between Ty elements located on sister chromatids can produce both duplications and deletions. Deletions between direct repeats can also occur by the single-strand annealing pathway (Fig 2B). **(B)** A crossover between repeats on opposite chromosome arms of sister chromatids can generate dicentric and acentric chromosomes. **(C)** An intrachromatid crossover between repeats on opposite arms can produce an acentric linear chromosome and a centromere-containing circle. **(D)** A crossover between Ty elements located on opposite arms in inverted orientation can generate two isochromosomes. **(E)** A crossover between inverted Ty elements located on the same arm results in an inversion in the segment located between the Ty elements. **(F)** Recombination between repeats located on non-homologous chromosomes can generate reciprocal translocations. Depending on the segregation pattern of the chromosomes, this event can produce cells that have a coupled terminal duplication and a terminal deletion. The two homologs are shown in red and blue.

The Ty1 retrotransposon is associated with two types of genomic alterations. First, a small fraction of spontaneous mutations in yeast are a consequence of insertion of a Ty element within or nearby the target gene [4,5]. In addition, since Ty1 elements represent non-allelic regions of sequence homology, homologous recombination between non-allelic Ty elements located on the same chromosome can result in a variety of chromosome rearrangements (Fig 1A–1E), and recombination between Ty elements located on non-homologous chromosomes can produce translocations [5–14] (Fig 1F). It should also be noted that DSBs located near but not within Ty elements can stimulate Ty-Ty recombination [15].

In most previous studies of Ty1-Ty1 interactions, events were selected to involve a specific region of the genome, either because of the method used to identify recombinants [4,7,8,13,15–17] or because of the placement of the targets for site-specific endonucleases [15–17]. In our experiments, we used the CRISPR/Cas9 system to target all Ty1 elements in a diploid genome and identified Ty1-Ty1 recombination events without selection except for viability. In addition, diploid strains were used to allow the identification of allelic mitotic recombination events. We also compared the effects of expressing CRISPR/Cas9 in haploid and diploid cells. A detailed comparison of our analysis and those of others will be reserved for the Discussion.

Non-allelic recombination between repeated transposons is also important in the generation of structural variants in human cells, some of which are disease-associated [18]. It is estimated that there are between one and four non-allelic homologous recombination events (mostly between Alu and L1 elements) per cell during development in humans. Diseases associated with Alu-Alu recombination are hypercholesterolemia, Fanconi anemia, von Hippel-Lindau syndrome, and others [19]. Thus, studies of this phenomenon in yeast are relevant to understanding genome evolution and pathogenesis in higher organisms.

### Pathways of homologous recombination

**Fig 2:**
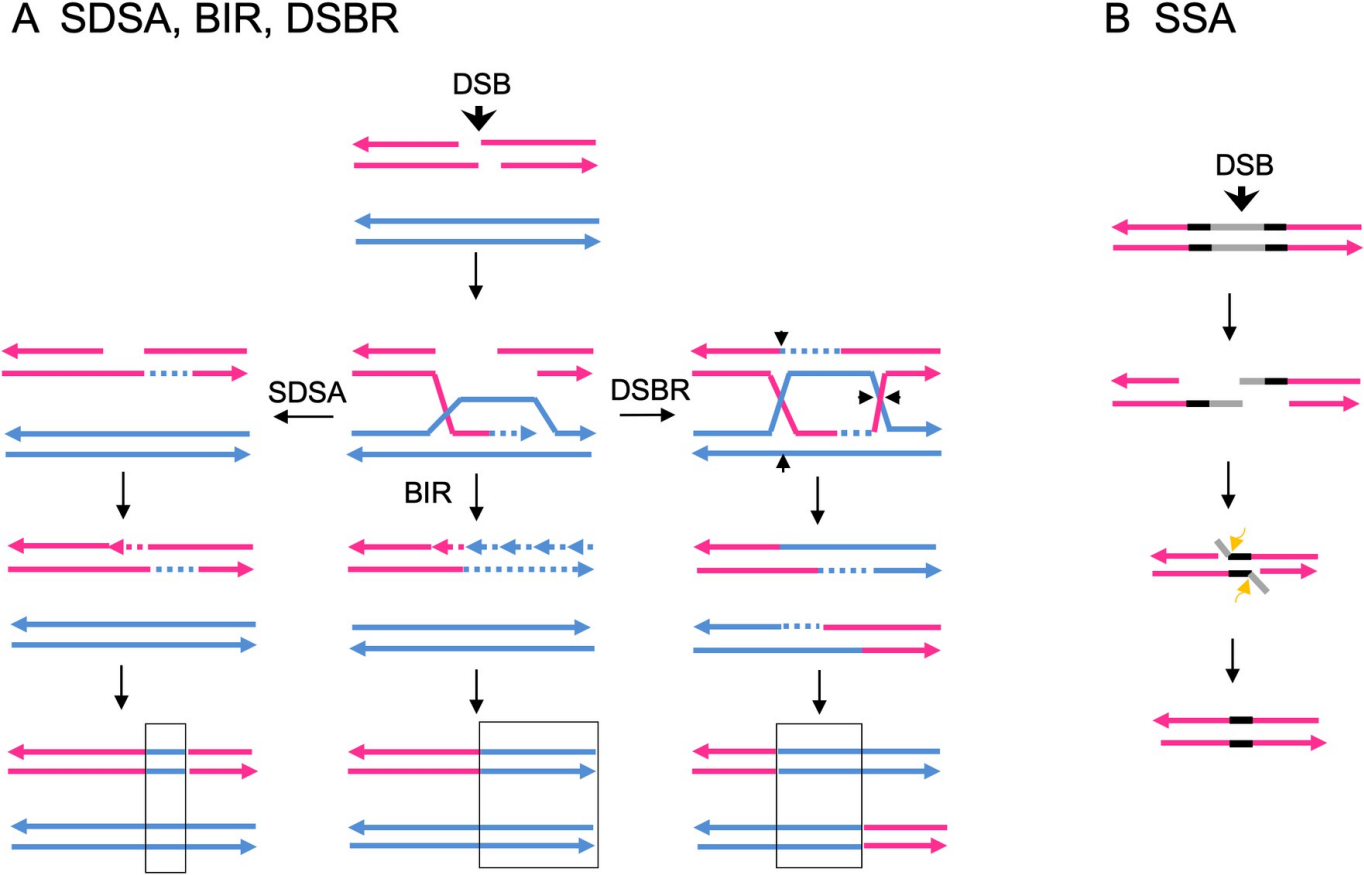
Pathways of homologous recombination. **(A)** Repair of a double-strand break (DSB) by synthesis-dependent strand annealing (SDSA), break-induced replication (BIR), and double-stranded break repair (DSBR). All pathways are initiated by a DSB shown by the vertical arrow, followed by 5’ to 3’ resection of the broken ends; the 3’ ends of the strands are marked with an arrow. The 3’ single-strand of one broken end invades the unbroken chromosome. DNA synthesis (dotted line) displaces a strand from the unbroken template. In the SDSA pathway, the invading strand is displaced and re-pairs with one of the broken ends. Mismatch repair of the heteroduplex (duplex formed from red and blue strands) results in a region of gene conversion (boxed) in which the flanking sequences are in the original parental configuration. In the BIR pathway, one of the broken products of the “red” chromosome is lost, and replaced by DNA synthesis from the unbroken template. This conservative synthesis involves the use of the blue strand as a template for one strand (continuous dotted line), and the new strand as a template for synthesis of the second strand (multiple short fragments with dotted lines). The net result of this process is a non-reciprocal LOH event that may extend to the end of the chromosome. The DSBR pathway is characterized by pairing between the displaced blue single-strand and one of the broken red strands. The resulting double-Holliday junction is resolved by cleaving the strands that connect the two duplexes. If the strands are cut as shown by the short arrows, the resulting duplexes have a region of conversion flanked by sequences in the recombinant configuration. It should be pointed out that extensive strand resection from the DSB may allow the recombination breakpoint to be displaced from the location of the DSB by >10 kb, as observed by Hoang *et al*. [15]. **(B)** Single-strand annealing (SSA) pathway. This intrachromosomal pathway is used to repair a DSB located between two repeated sequences (shown in black) that are separated by a region of non-repeated DNA (shown in gray). Resection of both ends exposes homology within the two repeats, allowing pairing. The unpaired single-strands are removed by endonucleases. The net result of this event is loss of one repeat and the intervening non-repeated sequence. Since Ty elements are flanked by 330-bp repeats, loss of the Ty and retention of one delta sequence may reflect the SSA pathway.

As described above, most large structural variations of chromosomes in *S*. *cerevisiae* reflect homologous recombination (HR) between non-allelic Ty elements [10]. There are a number of pathways of HR (Fig 2) [20]. In all of these pathways, the event is initiated by a processed single-stranded end invading either an allelic or non-allelic region of homology. If both ends are involved in the repair (double-strand break repair (DSBR) pathway), a double Holliday junction is formed. This junction can be resolved to generate a crossover of flanking sequences or a non-crossover, although we show only the crossover event in Fig 2A. The DSBR and other pathways have heteroduplex regions as intermediates, shown in the figure as a duplex in which the DNA strands are of different colors. If the heteroduplex contains mismatches, repair of those mismatches will result in either a gene conversion event (both chromosomes containing the same allele) or in a restoration event (maintenance of heterozygosity for the alleles). Gene conversion events unassociated with crossovers can also arise by the synthesis-dependent strand annealing (SDSA). In this pathway, following strand invasion and DNA synthesis, the invaded strand is displaced and reanneals to the other broken end. For DSB-induced events between dispersed repeats, conversions unassociated with crossovers are more frequent than conversions associated with crossovers [20]. In break-induced replication (BIR) events, one broken end of the chromosome is lost and the other end invades the unbroken chromosome, copying the unbroken chromosome to the end [21]. In wild-type yeast cells, DSBR-related crossovers are more common than BIR events [22]. Lastly, if a DSB occurs between two directly-oriented repeats such as the delta repeats in a Ty1 element, processing of the broken end followed by reannealing of the repeated sequences can produce a deletion [20]; this pathway is termed SSA, “single-strand annealing.” As described below, we detected most of the events shown in Fig 1, occurring by the pathways shown in Fig 2.

## Results

### Targeting Ty1 with CRISPR/Cas9

**Fig 3:**
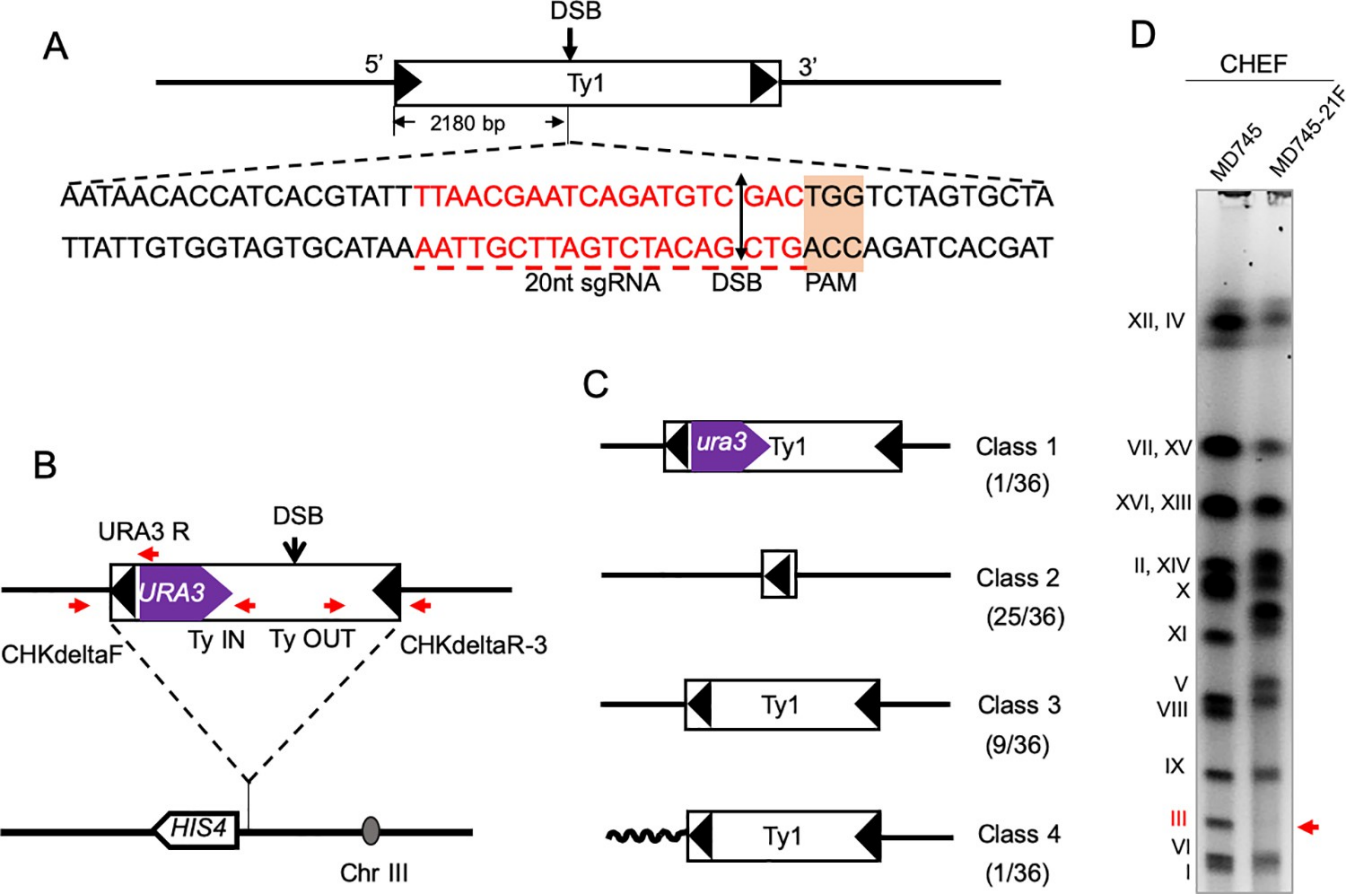
Sequence of CRISPR/Cas9 target in the Ty element, and diagnosis of events causing loss of *URA3* insertion from *Ty1*::*URA3*. **(A)** Sequence in Ty1 targeted by the guide RNA (shown in red). **(B)** Position of marked Ty element on chromosome III, and location of PCR primers used to diagnose 5-FOA-resistant isolates. The distance between the DSB site (CRISPR/Cas9 target) and the 3’ end of *URA3* is about 2.5 kb. Large black arrows show the LTRs (delta elements) associated with Ty1. **(C)** Different classes of 5-FOA-resistant isolates based on PCR analysis. The sizes of the fragments resulting from PCR reactions with various combinations of primers are given in S3 Table. The wavy line in Class 4 indicates a different flanking sequence from the original Ty1 element. **(D)** CHEF gel analysis of rearranged chromosome III in MD745-21F. The size of chromosome III was altered as a result of a crossover-associated gene conversion. MD745 is the control strain.

In our analysis of the effect of DSBs targeted to the Ty1 retrotransposon, we used strains containing the plasmid pMD97 which encodes a guide RNA directed to a DNA sequence conserved in Ty1 elements but not the other classes of *S*. *cerevisiae* transposons such as Ty2. The cut site is located about 2180 bp from the 5’ end of the 5’ terminal delta element (Fig 3A). In our experimental strains, the *CAS9* gene is regulated by the *GAL1*,*10* promoter that is integrated into the *HIS3* locus on chromosome XV. Thus, high levels of DSBs within genomic Ty elements are expected to be induced when the strains are grown in the presence of galactose, but not when grown in the presence of glucose. Details concerning the construction of the pMD97 plasmid and the yeast strains containing this plasmid are given in S1 Text, and S1 and S2 Tables.

### Analyzing CRISPR/Cas9-targeted Ty1 recombination events in haploid strains

To ensure that the CRISPR/Cas9 system would induce chromosome alterations, we first examined the effects of this system in a control experiment with the haploid strain FW588 (isogenic with S288c) [23]. In this strain, a Ty1 element containing the wild-type *URA3* gene is located upstream of the *HIS4* gene (*his4-912(URA3-b)*, resulting in a His^-^ phenotype [24]. The *URA3* gene is separated from the Cas9 cleavage site by about 2.5 kb (Fig 3B).

We constructed a haploid strain MD745 (details in S1 Text) containing *his4-912(URA3-b)* in addition to the plasmid pMD97 expressing the guide RNA and a galactose-regulatable *CAS9* gene; the control strain MD747 was isogenic with MD745 except it had a plasmid pAA2 that did not contain the guide RNA. Mitotic recombination events initiated within the *URA3*-marked Ty1 will often result in loss of the *URA3* gene [8]. Such strains will be 5-fluoro-orotate (5-FOA) resistant.

To induce cleavage in Ty1 elements, we placed cells of MD745 (containing pMD97), pre-grown in SD-leucine medium (2% glucose, 0% galactose) on solid medium lacking leucine and containing 2% raffinose and 0.01% galactose; the medium used in these experiments lacked leucine to force retention of the *LEU2*-containing pMD97 plasmid. Following colony formation, individual colonies were resuspended in water and appropriate dilutions were plated on SD-leucine medium and SD-leucine + 5-FOA medium. Based on the frequencies of 5-FOA-resistance (5-FOA^R^) derivatives in more than 30 individual colonies, we calculated a rate of 5-FOA^R^ using the method of the median [25,26]. A similar procedure was performed with the control strain MD747 that contains the plasmid pAA2 which lacks the guide RNA.

The rates of 5-FOA^R^ isolates in the control MD747 strain, when colonies were grown without galactose or with galactose were 5.2x10^-7^/cell division and 1.5x10^-6^/cell division, respectively. This result indicates that expression of Cas9 in the absence of the guide RNA has little effect on the formation of recombinogenic lesions. In contrast, in the MD745 strain, the rates of 5-FOA^R^ derivatives on glucose- and galactose-containing media were 1.6x10^-5^ and 3.6x10^-4^, respectively. Thus, elevated expression of Cas9 in a strain with the guide RNA increases the rate of 5-FOA-resistant colonies by about 240-fold relative to the strain without the guide RNA. The rate of 5-FOA^R^ derivatives in the MD745 strain grown in glucose (1.6x10^-5^), although 23-fold lower than the rate of the strain grown in galactose (3.6x10^-4^), is still about 30-fold higher than the rate observed in the control strain grown on glucose. The likely explanation of this result is that *GAL1* promoter results in a very low level of expression in cells grown in glucose [27] and, therefore, can induce a low level of Cas9 in the absence of galactose. It should also be pointed out that the rate of CRISPR/Cas9-induced events may be an underestimate, since more than 99% of the cells were killed by long-term expression of CRISPR/Cas9, and there may have been selection for cells with lower expression levels of the construct.

We confirmed the cutting of Ty1 elements by CRISPR/Cas9 by Southern analysis of MD745 (S1 Fig). *Xho*I cuts the LTR sequences associated with Ty1 sequences, producing a fragment of about 5.6 kb (S1A Fig). In the haploid strain MD745 containing pMD97 (expressing the guide RNA specific to Ty1) grown on galactose-containing medium (allowing expression of CRISPR/Cas9), we found two additional DNA fragments of sizes 3.7 and 1.9 kb, corresponding to the expected sizes of cleavage of the *Xho*I 5.6 kb fragment by CRISPR/Cas9 (S1B and S1C Fig). Assuming all Ty1 elements were cleaved with the same efficiency, we calculate that the efficiency of cutting of Ty1 elements in MD745 was about 8%. The control haploid MD747 (containing the plasmid lacking the guide RNA) had no bands corresponding to CRISPR/Cas9-induced cleavage.

We examined 36 independent 5-FOA^R^ isolates derived from MD745 grown in galactose by PCR. From previous results [8], we expected several classes of 5-FOA^R^ isolates: mutation of the embedded *URA3* gene (Class 1); replacement of the marked Ty1 with a solo delta elements as a consequence of recombination between the flanking delta elements (Class 2); gene conversion with a non-allelic Ty1 event unassociated with crossing over, resulting in loss of the *URA3* insertion but retention of the Ty element (Class 3); and gene conversion with a non-allelic Ty1 element associated with a crossover, resulting in a chromosome rearrangement (Class 4). To distinguish among these classes, we used PCR analysis with four sets of primers shown in Fig 3B. Based on the existence and sizes of the diagnostic PCR fragments (shown in S3 Table), we sorted the isolates into the classes described above that are shown in Fig 3C.

Class 1 (mutation of the *URA3* gene within the *his4-912(URA3-b)* allele) was an uncommon alteration, found in only 1 of 36 isolates. This result is expected since the rate of 5-FOA-resistant isolates in MD745 grown in galactose was 3.6 x 10^−4^/division, > 1000-fold higher than the rate of *URA3* point mutations found in other studies [28,29]. When we sequenced the gene in the Class 1 isolate, we found that it contained the same *ura3* mutation (G to A at position 701) as the mutant *ura3-1* gene on chromosome V. Thus, the Class 1 isolate is likely the consequence of a gene conversion event between the *URA3* gene on chromosome III and the mutant gene on chromosome V rather than a *de novo* mutation.

Replacement of the Ty element by a solo delta (Class 2) was the most common alteration, present in 25 of 36 isolates. This alteration could reflect either a crossover between the flanking delta elements (intrachromosomal or unequal sister-strand exchange, Fig 1A) or SSA between the delta elements (Fig 2B). Since the events are initiated by a DSB in the sequences located between the delta elements, SSA is the most likely mechanism for the event. Class 3 events, loss of the *URA3* insertion with retention of the Ty element, were also common (9 of 36 events). These events likely reflect a gene conversion event with a non-allelic Ty element that is unassociated with a crossover. Lastly, we found one Class 4 event. In this class, although the junctions of the Ty element with the flanking chromosome III sequences are conserved, based on PCR evidence, the left and right junctions are no longer contiguous, since no PCR product was observed with primers CHKdeltaF and CHKdeltaR-3 (Fig 3B and S3 Table). The simplest explanation of this pattern is that there was a gene conversion event between the *his4-912(URA3-b)* Ty and a non-allelic Ty that deleted the *URA3* gene, and this conversion event was associated with a crossover. By Clamped Homogeneous Electric Field (CHEF) gel analysis (Fig 3D), we confirmed that the wild-type chromosome III of the starting strain was missing in this isolate, confirming a chromosome III rearrangement.

We also examined the relative frequency of delta-delta recombinants and Ty1-associated rearrangements by long-read Nanopore sequencing of ten isolates of QL62, a haploid containing pMD97 that is closely related to (but not isogenic with) MD745. This sequencing method allows detection of delta-delta recombinants since the reads are often >20 kb. We found nine delta-delta recombinants and no chromosome rearrangements involving Ty1-Ty1 recombination.

In experiments similar to ours, Parket and Kupiec [30] found spontaneous recombination events involving a marked Ty in a haploid strain occurred at a rate of about 3x10^-6^/cell division. About two-thirds of these events were delta-delta recombination events and one-third were gene conversions with non-allelic Ty elements. Consistent with our results, translocations were very infrequent in the previous study. As described below, the spectrum of events observed in diploid strains expressing CRISPR/Cas9 was broader than that observed in haploids.

### System for analyzing CRISPR/Cas9-targeted Ty1 recombination events in diploid strains

Our subsequent experiments were performed in a diploid generated by crossing two sequence-diverged haploids, YJM789 and W303-1A. Since our study was not specific for any selected Ty1-Ty1 interaction, it was necessary to map the location of all Ty1 elements in the haploid parental strains in order to interpret the chromosome rearrangements. The details of this mapping are described in the S1 Text and the location of Ty elements in W303-1A, YJM789, and S288c (the reference strain in the Saccharomyces Genome Database (SGD)) are in the S1 Data and S2 Fig. The W303-1A strain had 37 Ty1 and 15 Ty2 elements, whereas YJM789 had only 17 Ty1 and 9 Ty2 elements. Most of the Ty1 elements in the two strains are in non-allelic positions (S2 Fig).

**Fig 4:**
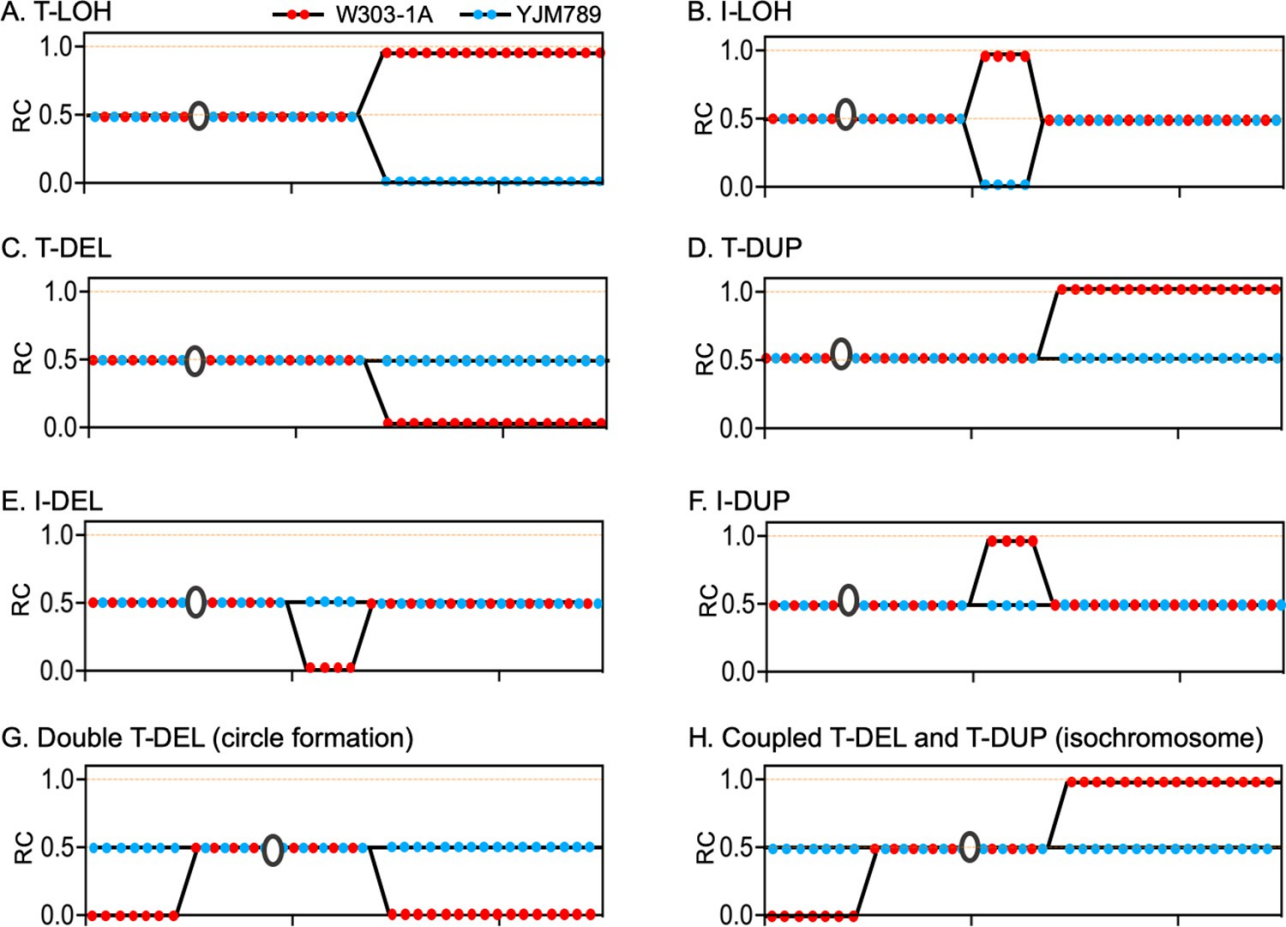
Depictions of various classes of LOH events along one chromosome as analyzed by DNA sequencing or microarrays. The Y-axis shows the ratio of coverage (RC), the number of “reads” for each SNP divided by the average “reads” of the two types of SNPs for the whole genome. Thus, a ratio of 0.5 indicates heterozygosity for a SNP. The RCs for each W303-1A- or YJM789-specific SNP are shown by red and blue circles, respectively. The open black circles indicate centromeres. The X-axis represents the SGD (Saccharomyces Genome Database) coordinate of the chromosome of the reference strain (S288c) numbered from the left to the right telomere. **(A)** Terminal LOH (T-LOH) event, reflecting a crossover or BIR. **(B)** Interstitial LOH (I-LOH) resulting from gene conversion or a double-crossover (less likely). **(C)** Terminal deletion (T-DEL). **(D)** Terminal duplication (T-DUP). **(E)** Interstitial deletion (I-DEL). **(F)** Interstitial duplication (I-DUP). **(G)** Double T-DEL or I-DUP that contains a centromere. **(H)** Coupled intrachromosomal T-DEL and T-DUP; isochromosome.

The diploids used in our study (MD704 and MD741) had the same insertion of the galactose-induced *CAS9* gene and CRISPR-containing plasmid pMD97 plasmid used in our haploid experiments. We expressed CRISPR/Cas9 for various lengths of time, and then examined multiple isolates by genomic sequencing or SNP-specific microarrays [31]. The diploids used in our analysis, in addition to being heterozygous for many Ty elements, were also heterozygous for about 55,000 single-nucleotide polymorphisms (SNPs). For these isolates, we determined the gene dosages for each SNP. This determination allowed us to distinguish both chromosome rearrangements (Fig 4) and mitotic recombination between homologs (S3 Fig).

In Fig 4, we show some of the patterns of sequence coverage (Ratio of Coverage as defined in the Fig 4 legend) that allowed us to identify the chromosome alterations; examples of the sequencing data are in S4 Fig. There are two types of events that result in loss of heterozygosity (LOH) of SNPs. In one class, the SNPs from one homolog are duplicated and the SNPs from the other homolog are deleted (for example, Fig 4A and 4B). In the second class, the SNPs from one homolog are duplicated or deleted without a compensating change in the SNPs of the other homolog (for example, Fig 4C and 4D). In this manuscript, we will reserve the LOH designation for events of the first type.

A reciprocal crossover or a BIR event results in a terminal LOH event (T-LOH, Figs 4A and S3), whereas an SDSA event (gene conversion) results in interstitial LOH (I-LOH, Figs 4B and S3). The pattern in which an isolate has both a terminal deletion (T-DEL) of one homolog (Fig 4C) and a terminal duplication (T-DUP) of a different homolog (Fig 4D) is usually a consequence of recombination between Ty1 elements located on different homologs forming a translocation (Fig 1F); additional experiments in support of this conclusion will be described below. Interstitial deletions (I-DEL, Fig 4E) or duplications (I-DUP, Fig 4F) are usually a consequence of recombination between directly-oriented Ty1s on one chromosome arm, reflecting unequal sister-chromatid exchange (Fig 1A), SSA, or intrachromatid crossing-over. A recombination event between directly-oriented Ty1 elements located on different arms of one chromosome (Fig 1C) would produce a double deletion (Fig 4G); although such an event would generate both a centromere-containing circle and a linear acentric fragment, the acentric fragment would likely be lost. An exchange between Ty elements in inverted orientation on different sides of the centromere could produce a T-DEL on one arm of the chromosome and a T-DUP on the other (Figs 1D and 4H). Aside from chromosome rearrangements, genomic sequencing can also identify loss or gain of whole chromosomes, or loss of one homolog with a duplication of the other (uniparental disomy, UPD).

The evidence that most of the alterations shown in Figs 1 and 4 are a consequence of Ty1-Ty1 recombination is primarily based on the demonstration that the breakpoints of the rearrangements contain Ty1 elements (S2 Data). However, not all chromosome rearrangements can be readily identified by short-read DNA sequencing alone. For example, recombination events between inverted repeats on one chromosome arm do not change sequence coverage, and thus they can only be identified using sequencing methods that yield “reads” of 10 kb or greater. Although we used such methods for a number of isolates, all samples were not examined by long-read sequencing. Another class that is not easily detected by sequence coverage analysis is reciprocal translocations in which both translocation products are segregated into the same daughter cell (balanced translocations). Similarly, reciprocal mitotic crossovers in which both recombinant chromosomes co-segregate do not result in LOH. As described below, we examined some of the isolates resulting from expression of CRISPR/Cas9 by other methods to detect such events including CHEF gel electrophoresis coupled with Southern analysis and long-read Nanopore sequencing.

### Cas9-mediated breaks at Ty1 elements stimulate global genomic alterations in the diploid genome

Two different protocols were used to generate diploids with CRISPR/Cas9-induced chromosome alterations. First, we grew cells with the galactose-inducible *CAS9* gene on solid medium for several days, allowing them to form colonies (long-term induction). Second, we limited the exposure of cells with the *GAL-CAS9* construction to two or four hours in galactose-containing medium (transient induction). Following exposure of the diploids to galactose, we selected derivatives of the strains that lost pMD97 (the plasmid containing the Ty1-directed guide RNA) on medium containing glucose for analysis of chromosome rearrangements. Although the same diploid strain was used for both types of experiments, the isolates from the long-term and short-term exposure were designated MD741 and MD704, respectively.

### Genomic rearrangements induced by long-term expression of CRISPR/Cas9

Long-term expression of CRISPR/Cas9 killed about 99.9% of the cells. After screening for independent survivors that lost pMD97, we examined the isolates by Illumina sequencing (details in S1 Text); some isolates were also analyzed by using SNP-specific microarrays [31]. The patterns of genomic alterations observed in this experiment and the location of the breakpoints of each event are given in the Dataset S2.1 in S2 Data with depictions of each class of event in the Dataset S2.2 in S2 Data.

The numbers of each type of chromosome rearrangement or mitotic recombination event are shown in Table 1. The total number of events in 18 isolates was 58, about 3 per isolate. After one cycle of growth from a single cell to a colony in galactose-containing medium, this frequency of chromosome rearrangements was much greater (>100-fold) than observed in an isogenic wild-type strain that lacked the CRISPR/Cas9 construct [12]. We did not estimate a rate of genomic alterations for MD741 because we found that most (14 of 18) isolates lost the *CAS9* gene during growth of the colony as the consequence of a mitotic event on chromosome XV (the location of the heterozygous *CAS9* gene) or loss of the chromosome XV (Dataset S2.1 in S2 Data and S3 Data). For this reason, we also performed experiments in which we measured rates of instability in strains transiently exposed to CRISPR/Cas9 (as described below).

**Table 1:** Summary of homologous recombination events for diploid strains with long-term and transient CRISPR/Cas9 expression.

Recombination Class^a^	MD741 (long-term exposure)	MD704 (transient exposure)		WT^b^	
	Number of events	Number of events	Rate of events (normalized to WT)	Number of events	Rate of events
**Intrachromosomal alterations**					
Large deletions^c^	5	12	6.32E-01 (14000)	12	4.55E-05
Large duplication^d^	1	4	2.11E-01 (9300)	6	2.27E-05
Circle formation^e^	1	1	5.26E-02 (14000)	1	3.79E-06
Isochromosome^f^	3	0	0	0	0
Complex events^g^	1	0	0	0	0
**Translocations**					
Simple translocations	11	12	6.32E-01 (170000)	1	3.79E-06
Complex translocations^h^	1	3^i^	1.58E-01	0	0
**Allelic recombination** ^**j**^					
I-LOH	19	9	4.74E-01 (150)	859	3.25E-03
T-LOH	16	13	6.84E-01 (510)	356	1.35E-03
Total Events	58	54	2.84E+00 (610)	1235	4.68E-03

^a^Only breakpoints involving Ty1 elements are counted in this table for intrachromosomal alterations and translocations.

^b^The rates (per cell division) of recombination events in the wild-type strain were measured by Sui *et al*. [12].

^c^Only large internal deletions (>1kb) with Ty1 elements at both breakpoints are included in this category, including classes h1 and h2 in Dataset S2.2 in S2 Data.

^d^Only large internal duplications (>1kb) with Ty1 elements at both breakpoints are included in this category, including classes g1 and g2 in Dataset S2.2 in S2 Data.

^e^Terminal deletions occurred on both the left and right arms of the same chromosome, and PCR verification confirmed chromosomal circularization.

^f^This category includes recombination within the same chromosome, a terminal deletion at one arm end combined with terminal duplication at the other arm.

^g^This category includes events with multiple transitions (>3) between homozygous and heterozygous regions in the same chromosome.

^h^This class represents for translocations between different chromosomes with more than two transitions.

^i^The complex pattern LOH events on chromosomes II and III were counted as one complex translocation in isolate MD704-4h-2. Also, the rearrangements involving chromosomes III, VII, and XIII were classified as one complex translocation in isolate MD704-4h-6P.

^j^All LOH events were included in this table. I-LOH (interstitial LOH events) includes Classes a1, a2 and c1 in Dataset S2.2 in S2 Data. T-LOH (terminal LOH events) includes Classes b1, b2, b3, d1 and d2 in Dataset S2.2 in S2 Data.

As shown in Table 1, although both intrachromosomal alterations and translocations were common among the MD741 isolates, allelic mitotic recombination events (I-LOH and T-LOH) were also common. We also observed five monosomic chromosomes and two uniparental disomy events (Dataset S3.1 in S3 Data) [32].

Since we targeted Ty1 elements with CRISPR/Cas9, we expected most of the chromosome alterations to have Ty1 elements at their breakpoints. This expectation was met for the I-DEL and I-DUP events, and the T-DEL and T-DUP events in which 6 of 6 events (I-DEL or I-DUP) have Ty1 elements at both breakpoints; and 31 of 32 events (T-DEL and T-DUP), respectively, had Ty1 elements at their breakpoints. The large I-DEL and I-DUP events likely reflect unequal crossovers between non-allelic Ty elements located on the same chromosome arm (Fig 1A), whereas the coupled T-DEL and T-DUP events are a consequence of recombination between Ty elements on non-homologous chromosomes (Fig 1F). Of the 17 T-DEL events, 15 were associated with a T-DUP event in the same isolate. Further evidence that these paired deletions/duplications are a consequence of translocations will be described below. In contrast to the interstitial and terminal deletion/duplication events, of the 35 LOH events, only one had a Ty1 element at the breakpoint; breakpoints were defined as the region between heterozygous SNPs for I-LOH events and as 20 kb “windows” centered at the midpoint of heterozygous and homozygous SNPs for T-LOH events [12,31].

We also examined single-base mutations among the isolates. Among the 18 samples, we found 13 single-base alterations and one single-base deletion (S4 Data). As expected, all induced mutations were heterozygous (S4 Data).

### Genomic rearrangements induced by transient expression of CRISPR/Cas9

Since a large fraction of the diploids expressing CRISPR/Cas9 for many cell divisions lost the heterozygous *CAS9* gene as a consequence of mitotic recombination or chromosome loss (as described above), we examined patterns of genomic alterations in the same diploid strain (MD704) following transient expression of CRISPR/Cas9. The diploid was pre-grown for three days on solid medium containing only glucose and lacking leucine. The strain was then incubated in liquid medium containing 2% galactose for two or four hours. Following exposure to galactose-containing medium, the cells were allowed to form colonies on rich growth medium containing glucose. For both the two-hour and four-hour samples, viability was >75% following the galactose incubation. DNA was isolated and sequenced from nineteen colonies, nine from the two- and ten from the four-hour samples; results were similar for both types of samples. By Southern analysis, about 4% of the Ty1 elements in diploid strains that were transiently exposed to CRISPR/Cas9 were cut (S1 Fig).

The detailed data are in the Dataset S2.3 in S2 Data and are summarized in Table 1. Since the results for the two- and four-hour samples were very similar, we show the sum of these conditions in Table 1. Assuming that the genomic alterations occur during the first cell division in galactose-containing medium, we can calculate the rates of each class of event compared to the rates in the isogenic wild-type strain; this rate estimate is approximate, since we cannot exclude the possibility that the CRISPR/Cas9 activity persists for several generations after the cells are transferred to glucose-containing medium. The rates of most types of chromosome alterations were elevated by two to three orders of magnitude (Table 1).

Aneuploidy was also very significantly elevated in the strains exposed to galactose for two or four hours. We observed 12 monosomic, one trisomic, and two uniparental disomic chromosomes (Dataset S3.2 in S3 Data). The rate of aneuploidy per cell division is 7.9x10^-1^, greater than 1000-fold higher than observed in wild-type cells [12]. The very high rate of monosomy is consistent with the expected high rate of DSB formation and incomplete repair of DSBs, resulting in chromosome loss.

We observed only a small number of mutations (six) in cells with transient exposure to CRISPR/Cas9 (S4 Data). The calculated rate of mutations/base pair/cell division was 1.4x10^-8^ or about 70-fold higher than observed in an isogenic wild-type diploid [12]. This estimate, however, is based on a small number of events.

### Properties of the genomic rearrangements associated with expression of CRISPR/Cas9

**Fig 5:**
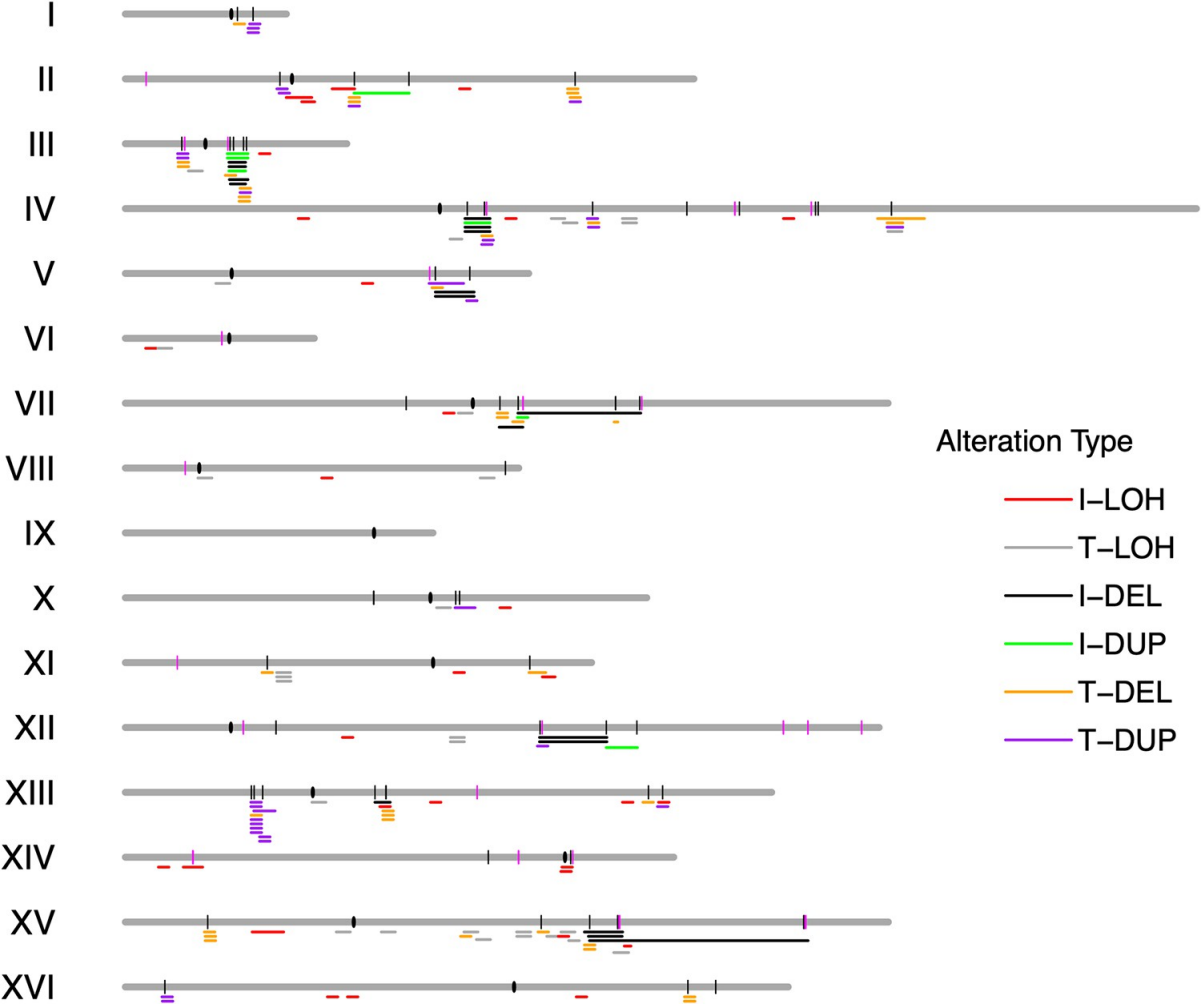
Locations of CRISPR/Cas9-induced genomic alterations relative to the positions of Ty elements. The locations of Ty1 and Ty2 elements are shown as short vertical black or red lines, respectively, and the centromeres are shown as black ovals. Two partial Ty elements with the CRISPR/Cas9 target (one at 803 kb on IV and one at 215 kb on XII) are also shown as black lines. The breakpoints for various types of events are indicated by horizontal lines of different colors. The color code for genomic alterations is: I-LOH (red), T-LOH (gray), I-DEL (black), I-DUP (green), T-DEL (yellow), T-DUP (purple). Most I-LOH and T-LOH events are not associated with Ty elements at their breakpoints, whereas the breakpoints of I-DEL, T-DEL, I-DUP, and T-DUP events are associated with Ty elements.

We observed 151 chromosome rearrangements (excluding aneuploidy) among the isolates obtained from transient or long-term expression of CRISPR/Cas9. As shown in S2 Data and Fig 5, the breakpoints of most (91 of 92) T-DELs or T-DUPs, and I-DELs and I-DUPs had Ty1 elements or delta sequences at the breakpoints. In contrast, only 12% (7 of 59) of the breakpoints of I-LOH (reflecting gene conversions) and T-LOH events (reflecting crossovers or BIR) involved Ty1 elements. Possible explanations for this observation will be given in the Discussion.

The locations of the genomic rearrangements determined in our study are shown in Fig 5. From this figure, both the association of I-DEL, I-DUP, T-DEL, and T-DUP with Ty1 elements is clear, as is the lack of association between LOH breakpoints and Ty1 elements. Although the Ty1-associated recombination events were broadly distributed among the yeast chromosomes, two Ty1 elements were associated with significantly elevated levels of chromosome rearrangements, the Ty1 element located on chromosomes III (*W-IIIR-WTy1-5*, p<0.001) and IV (*Y-IVR-CTy1-2*, p = 0.03). The “hot” Ty1 element on chromosome III is adjacent to an inverted Ty1 element, and together these elements were shown to be a fragile site in strains with low levels of DNA polymerase alpha [33]. The “hot” Ty1 element on chromosome IV is about 25 kb of the Ty1 *Y-IVR-CTy1-1*. Since many of the events observed for these two elements involve closely-linked Ty1s, it is possible that these elements are not preferred targets of CRISPR/Cas9, but have an elevated probability of recombination with the neighboring element.

Although Ty2 does not contain the target site for the guide RNA used in our experiment, Ty1 and Ty2 share considerable homology and a DSB induced in Ty1 might be able to recombine with the 24 copies of Ty2 in the MD704/MD741 genomes. The average identity between Ty1 and Ty2 in the S288c background is about 74%, although there are numerous blocks of identity 250–750 bp in length; the levels of sequence identity for Ty1-Ty1 or Ty2-Ty2 comparisons are about 96% for both [15]. Nonetheless, of 124 breakpoints involving at least one Ty1 element, 118 were Ty1-Ty1, only 5 were Ty1-Ty2, and 1 was Ty1-delta. This strong preference for Ty1-Ty1 interactions may reflect the sequence diversity around the CRISPR/Cas9 target site between Ty1 and Ty2 (average of 75% in the 500 bp flanking the target). Mismatches within the heteroduplex would be expected to reduce the frequency of exchange [34],

Hoang *et al*. [15] found that there was a 50-fold preference for intrachromosomal non-allelic Ty interactions over interchromosomal non-allelic interactions. We examined the same issue in our data (details of the calculations in S1 Text). Considering all possible interactions of Ty1 elements that would give a detectable change in gene dosage, if there is no preference for intrachromosomal or interchromosomal recombination, the expected proportion is 0.10 for intrachromosomal events and 0.90 for interchromosomal events. The observed number of intrachromosomal and interchromosomal Ty-Ty events were 43 and 52, respectively; by chi-square analysis, the excess of intrachromosomal events is very significant (p<0.001). The magnitude of the excess of intrachromosomal events (about five-fold) is considerably less in our study than in that of [15], possibly because their study involved DSBs induced on the right arm of chromosome III which has a very high density of Ty elements. The preference for intrachromosomal recombination is also observed for duplications of non-Ty1 repeats [35].

### Physical analysis of translocations and other chromosome rearrangements by CHEF gel electrophoresis, microarray analysis and/or by Nanopore sequencing

As described above, analysis of short-read DNA sequencing and DNA microarrays indicated that large chromosome alterations (large deletions and duplications, as well as putative translocations) could be induced by expression of CRISPR/Cas9. To provide additional evidence for these conclusions, we analyzed genomic DNA from some of the MD741 (long-term expression of CRISPR/Cas9) and MD704 (short-term expression of CRISPR/Cas9) isolates by CHEF gels (allowing the separation of intact chromosomal DNAs), PCR analysis, and/or Nanopore sequencing.

**Fig 6:**
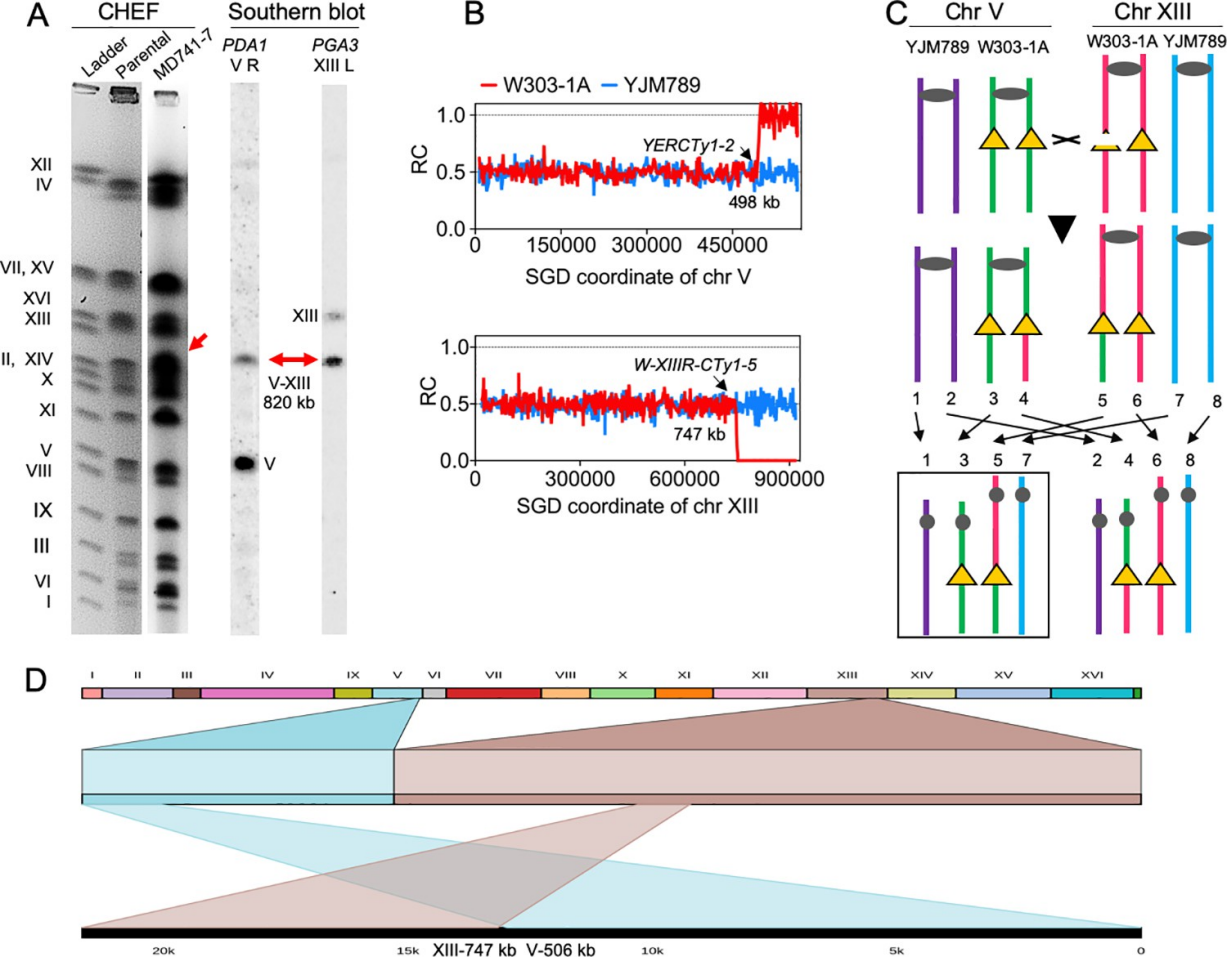
Analysis of the translocation in MD741-7. **(A)** CHEF gel showing a novel chromosome band at 820 kb. This chromosome hybridizes to probes derived from the right end of chromosome V and the left end of chromosome XIII. **(B)** Sequencing data showing a T-DUP on chromosome V and a T-DEL on chromosome XIII; both breakpoints are at Ty1 elements (Dataset S2.1 in S2 Data). **(C)** Production of the translocation by crossing-over between Ty elements (shown as yellow triangles). The translocation could also result from a BIR event (Fig 6B). **(D)** Nanopore sequencing of a chromosome V-XIII translocation (depiction by Ribbon software). The breakpoints of the translocation are 493,149 on V (close to a Crick-oriented Ty at 493 kb, S2 Data) and 748,223 (close to a Crick-oriented Ty at 748 kb, S2 Data) on XIII.

The detailed description of these chromosome rearrangements will be given in the S1 Text and SI Figures. Here, we restrict the discussion of these rearrangements to a single example, a translocation in the isolate MD741-7. CHEF gel analysis of DNA from MD741-7 showed a novel chromosome of about 820 kb (Fig 6A). Whole-genome sequencing detected a T-DUP on chromosome V near coordinate 498 kb (the location of *YERCTy1-2*) and a T-DEL on chromosome XIII near coordinate 747 kb (the location of a Crick-oriented Ty1 element) (Fig 6B). Based on the location of these elements and the sizes of the unrearranged chromosomes, a reciprocal crossover between these two Ty elements would produce two translocations of about 820 kb and 670 kb. Segregation of the 820-kb translocation and untranslocated copies of chromosomes V and XIII would generate an isolate with the observed deletion-duplication pattern (Fig 6C). As expected, the 820-kb translocation hybridizes to probes from the left end of XIII (*PGA3*) and the right end of V (*PDA1*). Alternatively, the same pattern could be produced by a BIR event initiated by a DSB in the Ty element on chromosome XIII.

We also confirmed the translocation between V and XIII by using long-read Nanopore sequencing (Fig 6D). For displaying the Nanopore data, we use Ribbon software [36]. In this display, the 16 chromosomes are shown in different colors at the top of the figure with chromosomes XIII and V colored in brown and light blue, respectively. At the bottom of Fig 6D, the diagram shows a segment of DNA with a fusion of sequences from chromosomes XIII and V with the approximate locations of these sequences within the chromosome indicated in the upper and middle portions of the diagram. Both segments were located near the right ends of the two chromosomes, and both segments contained Crick-oriented Ty1 elements. The novel arrangement included a Ty1 element at the breakpoint.

With similar methods, we analyzed nine other translocations in MD741-3, MD741-5, MD741-6, MD741-8, and MD741-9 (S1 Text and SI Figures). Although most of the isolates had only one translocation, MD741-9 had a pair of III-IV and IV-III balanced translocations (S15 Fig). This result argues that at least some of the events are reciprocal crossovers rather than BIR events. We also did detailed examinations of four isolates of MD704 (S1 Text). We detected three examples of recombination between Ty1 elements arranged in inverted orientation on opposite chromosome arms (for example, as shown in S11 Fig), but no recombination events between inverted repeats located on the same chromosome arm.

As described above, in Nanopore sequencing of ten haploid isolates in which the Ty1-directed CRISPR/Cas9 was expressed, we found nine examples of replacement of a Ty1 element with a solo delta element (a “pop-out” likely due to single-strand annealing) and no examples of a chromosome rearrangement reflecting Ty1-Ty1 recombination. We also examined five diploid strains expressing CRISPR/Cas9 (two MD741 and three MD704) by Nanopore sequencing and detected only one “pop-out” and eight Ty1-Ty1 recombination events. The differences in the recovery of these two classes of events in haploids and diploids is very significant (p < .001 by Fisher exact test). We also examined whether Ty1 elements in haploid strains expressing CRISPR/Cas9 had small deletions of the target site as expected if the DSBs could be repaired by non-homologous end-joining. No such deletions were found in examining 185 Ty1 elements.

### Analysis of the location of breakpoints within Ty elements using Nanopore sequencing

**Fig 7:**
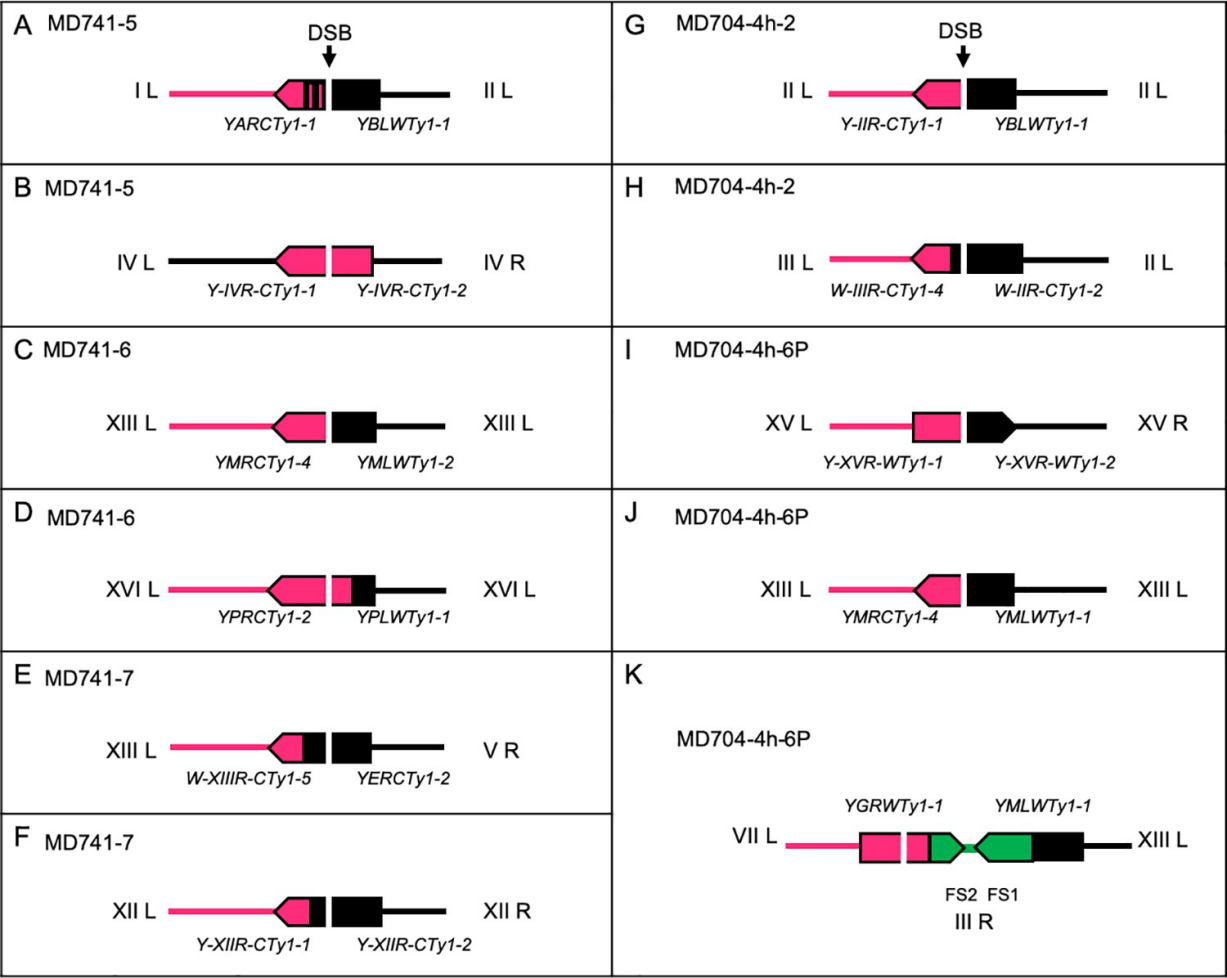
Breakpoints within Ty elements as determined by Nanopore sequencing. Since Ty elements often have polymorphisms that distinguish different elements, we can sometimes determine the breakpoints within the Ty elements involved in homologous recombination. These Ty-Ty recombination events sometimes involve elements located on non-homologous chromosomes (giving rise to translocations; A, E, H, and K) or elements located on the same chromosome (giving rise to I-DEL, I-DUP or other rearrangements; B, C, D, F, G, I, and J). The position of the target guide RNA is indicated by an arrow marked “DSB” and different colors represent sequences derived from different elements. In some of the recombination events (C, G, I, and J), the breakpoint between the contributions of the different Ty elements to the recombinant element is near the DSB site. In most of the other events, the transition between the different Ty elements does not coincide exactly with the DSB site. In these cases, it is likely that the repair of the DSB was associated with a gene conversion event. Lastly, one of the isolates (K) was associated with a tripartite recombination event between Ty elements located on three different chromosomes as illustrated in S24 and S25 Figs.

In addition to using Nanopore sequencing to confirm translocations and other chromosome rearrangements, we used long-range sequencing to map the breakpoints of the recombination events within the Ty1 elements for a sub-set of the rearrangements. The approach is feasible since non-allelic Ty1 elements often have multiple polymorphisms that distinguish one element from the other. Our summary of the mapping of eleven events is shown in Fig 7. We analyzed hybrid Ty elements associated with four translocations, four intrachromosomal recombination events resulting in a deletion of one chromosome arm and a duplication of the other arm, and three internal deletion events. For each event, we detected a transition between the SNPs of the heterozygous Ty elements within the Ty element. As expected, since the repair of DSBs is associated with a region of gene conversion (Fig 2), the transition between the SNPs of one Ty element and those of the other was usually close to, but not at, the position of the CRISPR/Cas9 target sequence (S5 Fig).

A detailed analysis of the breakpoints within the Ty elements shows one other point of interest. In the isolate MD741-6, an isochromosome was formed by recombination between Ty1 elements on the right (*YPRCTy1-2*) and left (*YPLWTy1-1*) arms. This rearrangement can be formed either by a BIR event or by a reciprocal crossover. As shown in S6 Fig, in a BIR event, the breakpoint between SNPs is likely to be located at the site of the DSB, whereas for a reciprocal crossover, the breakpoint can be displaced from the DSB. The observation that the breakpoint is displaced from the DSB site (S5 Fig) argues that this event is likely a crossover rather than a BIR event. We cannot rule out, however, that mismatch correction occurs during the initial strand invasion step of BIR resulting in a displacement of the observed breakpoint from the position of the DSB.

## Discussion

As described in the Introduction, genomic rearrangements resulting from ectopic recombination between Ty elements have been observed in many studies, our study analyzed the nature of genomic alterations in a diploid in which all Ty1 elements are potential targets for CRISPR/Cas9, and in which both allelic and non-allelic recombination can be detected. We also examined Ty1-associated genomic events in haploids as well as diploids. Our main conclusions are: (1) The types of recombination events observed in haploid strains are different from those observed in diploid strains; (2) In diploid strains, expression of CRISPR/Cas9 greatly increases the frequency of large deletions, duplications, translocations, and other chromosome rearrangements reflecting homologous recombination of non-allelic Ty1 elements; (3) Many different Ty1 elements were involved in the rearrangements, although two Ty elements had significantly more events than the average; (4) Although Ty1 and Ty2 share homology, almost all rearrangements were between two Ty1 elements; in addition, Ty1-Ty1 recombination events are biased toward intrachromosomal events; (5) Using polymorphisms that distinguish Ty1 elements, we showed that the breakpoint of the Ty1-Ty1 was usually near, but not at, the CRISPR/Cas9 target site; (6) Unlike the chromosome rearrangements, only a small fraction (12%) of the allelic mitotic recombination events had Ty1 elements at their breakpoints. Below, we will emphasize only those issues not previously discussed in the Results section.

### Comparisons of Ty1-Ty1 interactions in haploid and diploid strains

In haploids, the events were biased to delta-delta recombinants, presumably the result of SSA. In diploids, in contrast, most Ty1-related events were crossovers or BIR between non-allelic Ty1 elements. Although our initial observations in haploids and diploids utilized two different methods for detecting genomic rearrangements, this conclusion was subsequently confirmed by Nanopore sequencing in both haploids and diploids. Note however, that our haploid strains were isogenic or very similar to S288c/W303-1A, whereas the diploid was a hybrid of W303-1A and the sequence-diverged YJM789 strain. Thus, experiments in which a homozygous diploid is generated from W303-1A by mating type switching are needed to strengthen our conclusion.

Our observations concerning the relative frequencies of pop-outs and Ty1-Ty1-associated crossover/BIR events are consistent with previous observations of Kupiec and co-workers [8,30]. In haploid strains with a single marked Ty1 element, Parket and Kupiec [30] found that two-thirds of the events resulting in loss of the insertion were a consequence of delta-delta recombination, whereas in diploids with a *Ty1*::*URA3* insertion, only 10% (4 of 42) of the ectopic recombination events causing loss of *URA3* were the result of delta-delta recombination [8]. Loss of a marker by delta-delta recombination is likely to reflect a different recombination pathway (single-strand annealing) than ectopic exchanges that delete the marker (SDSA and/or DSBR). One possibility is that a gene required for SSA, but not for SDSA and DSBR, is expressed at higher levels in haploids than in diploids. No such candidate gene is known, although only a limited number have been tested.

Another explanation is that more extensive resection is required for delta-delta recombination than for non-allelic SDSA or DSBR, and that resection is more extensive in haploids than diploids. Such a difference has not been reported. It has been suggested by Agmon *et al*. [37] that there are competing pathways for homology searches involving intrachromosomal repeats and interchromosomal repeats. Although the details of this competition have not been elucidated, it is possible that haploids more effectively engage in an intrachromosomal homology search than diploids. It is also possible that heteroduplexes between non-allelic Ty1 elements are usually resolved without crossovers in haploids but are often associated with crossovers in diploids. Alternatively, BIR events may be more processive in diploids (resulting in translocations and other chromosome rearrangements) than in haploids. Regardless of the mechanisms involved, these results argue that the genomes of haploids and diploids evolve by qualitatively different pathways.

### LOH events in diploid strains expressing Ty1-targeted CRISPR/Cas9 do not directly involve Ty elements

Besides Ty1-mediated chromosome rearrangements in the diploid strains MD704 and MD741, we found that LOH events (sum of I-LOH and T-LOH) were elevated about 280-fold in cells expressing CRISPR/Cas9 compared to the wild-type strain [12]. Strikingly, most of these LOH events did not have Ty elements at the breakpoints. In a previous study by Fleiss *et al*. [16] in which a CRISPR/Cas9 guide RNA was targeted to a sub-set of Ty3 LTRs, it was observed that some of the chromosome rearrangements did not have the appropriate targets at the breakpoints. They suggested that some of the DSBs could generate chromosome rearrangements by a template-switching mechanism that did not require extensive sequence homology [38]. Since the LOH events in our study likely involve extensive sequence homology, this mechanism does not apply to our observations.

Although we cannot offer a single explanation for the stimulation of mitotic recombination in genomic regions without Ty elements, a number of possibilities exist. First, the guide RNA used in our CRISPR/Cas9 experiments may produce “off-target” DSBs. We do not favor this explanation for two reasons. In BLAST searches of the yeast genome with the guide RNA sequence, no non-Ty sequence is found. Based on BLAST searches with guide RNA sequences in which we introduced sequence changes *in silico*, related sequences with one or two mismatches could have been detected, but were not observed. In addition, if one or a few non-Ty genomic sequences were recognized by the guide RNA, we would expect that the non-Ty LOH events would have strongly preferred breakpoints. No such preferences were observed.

A second possibility is that a fraction of Ty-Ty recombinants produces dicentric chromosomes. Breakage of a dicentric could produce recombinogenic broken ends that do not have Ty elements at the termini. Although it is likely that a substantial fraction of the Ty-Ty recombination events results in formation of a dicentric, the cycles of repair and breakage required to produce monocentric products likely select against such isolates. A third explanation is that a strain with multiple DSBs may alter the ratio of breaks repaired by sister-strand recombination (an event that does not lead to LOH) in favor of recombination between homologs. Sister-strands are used as a template for repair of DSBs with a preference of at least 10-fold over the homolog [22,39]. By this model, the frequency of DSBs targeted to non-Ty sequences is not altered in strains expressing CRISPR/Cas9. A related possibility is that large numbers of DSBs result in an arrest of the cell cycle in a recombination-prone stage.

Hoang *et al*. [15] showed the DSBs located more than 10 kb from a Ty element could stimulate recombination events involving that element, suggested very long excision tracts from the broken ends could stimulate exchange. This observation raises the possibility that DSBs within the Ty element could stimulate recombination at sites distal to the broken Ty1. One observation consistent with this possibility is that there is a strong bias (24:1) for T-LOH events to lose W303-1A sequences and duplicate YJM789 sequences (S2 Data); this bias is not observed in isogenic wild-type cells that do not express CRISPR/Cas9 (151 T-LOH events homozygous for YJM789, 129 homozygous for W303-1A) [12]. One plausible explanation for the bias is that the W303-1A-derived homologs break more frequently because of the higher density of Ty1 elements on these homologs, and these breaks are then repaired by BIR using the YJM789 homologs as templates. This model predicts that the Ty event that is responsible for the T-LOH event would be located centromere-distal to the observed breakpoint (S26A Fig). We measured the distances between the T-LOH breakpoints and the closest centromere-distal Ty1 elements in a wild-type diploid not expressing CRISPR/Cas9 [12] (S26B Fig) and an isogenic strain expressing CRISPR/Cas9 (S26C Fig). The distribution is skewed toward smaller distances in the strain expressing CRISPR/Cas9, consistent with the hypothesis that the T-LOH events that do not have Ty1 at the breakpoint may nonetheless be induced by Ty1-associated breaks.

However, there are several observations that suggest that all LOH events are not initiated by extensive process of DSBs within Ty1 elements. First, I-LOH events do not show the same bias toward DSB formation on the W303-1A homolog. Of the I-LOH events, 13 were homozygous for YJM789 alleles and 15 were homozygous for W303-1A alleles. In addition, expression of CRISPR-Cas9 elevated T-LOH about 500-fold, but I-LOH only 150-fold (Table 1). Thus, either the initiation or subsequent repair events for T-LOH and I-LOH events must be different.

Hicks *et al*. [40] showed that BIR elevated mutation rates more than 1000-fold. From the data in the S2 Data, we calculated that 0.025 of the total genomes of 27 isolates were in regions of T-LOH. Of the 20 mutations detected in these strains, two were in these T-LOH regions and 18 were outside these regions. By chi-square analysis, mutations were not significantly over-represented in the T-LOH regions (p = 0.15). This conclusion, however, is based on a small number of mutations.

Currently, we do not have data that distinguish among these models. It is likely that T-LOH events, as well as other chromosome alterations, can be produced by both BIR and reciprocal crossovers. As described above, we found one isolate (MD741-9) with reciprocal translocations, arguing that reciprocal crossovers can be formed between non-allelic Ty elements. It is important to consider the possibility that yeast strains with large numbers of DSBs or DSBs that undergo multiple cycles of breakage may utilize recombination pathways differently than wild-type strains with very low levels of breaks observed in uninduced cells. In wild-type cells grown under non-stressed conditions, most T-LOH events occur by reciprocal crossovers rather than BIR [22]. It is possible that BIR events are more common in strains attempting to repair multiple breaks.

### Closing thoughts

In summary, expression of CRISPR/Cas9 directed to the Ty1 elements is an efficient method of generating a variety of novel types of chromosome rearrangements. Given the ease with which such alterations are generated, it is perhaps surprising that most (80%) of the genomes of *S*. *cerevisiae* strains obtained from the wild are co-linear [41], and co-linearity is also observed among several *Saccharomyces* species that are closely related to *S*. *cerevisiae* [42]. This observation suggests that the arrangement of genes among the 16 chromosomes may be under genetic selection. Two other important issues raised by our study are: 1. The degree to which recombination events in haploids and diploids are different, and 2. Whether the patterns of recombination in cells that receive large numbers of DSBs or repeated cycles of DSB formation are different from cells that receive single DSBs (as expected from spontaneous events).

## Materials and methods

### Strains and plasmids

The genotypes of the yeast strains used in this study are shown in S1 Table, and constructions of strains and plasmids are described in S1 Text.

### SNP microarray analysis

A custom SNP microarray designed in our previous studies was used to detect the genomic alterations in diploid strains [31,43]. In brief, the control DNA extracted from the heterozygous strain JSC24-2 was labeled with Cy3-dUTP, and the experimental DNA was labeled with Cy5-dUTP. The labeled DNA was mixed and competitively hybridized at 62°C. A GenePix scanner and GenePix Pro-6.1 software were used to examine the ratio of hybridization of the control and experimental DNA. The designs of the array are on the Gene Expression Omnibus (GEO) dataset, with the accession number of GPL20144.

### Illumina sequencing

Genomic DNA of yeast cells was extracted using the EZNA yeast DNA kit (Omega, Doraville, USA). Whole-genome Illumina sequencing was performed on a NovaSeq 6000 platform using a 150-bp paired-end indexing protocol. Data analysis was done as described previously [12]. In brief, the software BWA [44] was used to align reads to the reference genome of S288c (sacCer3). The output.sam files were processed by Samtools [45] and VarScan 2 [46] to detect sequencing coverage and mutations.

### PacBio sequencing

The genomic DNA of the strain JSC20-1 (YJM789 background) was extracted using a modified CTAB DNA Extraction method [47]. Standard sequencing library construction was carried out with the PacBio DNA Template Prep Kit 2.0. Genome sequencing was performed on a Pacific Biosciences RS II instrument with P6C4 chemistry. Assemblies were carried out using the software Falcon [48], followed by mapping the reads back to the assembled sequence with BLASR [49]; consensus calling was done with Arrow software [50].

### Nanopore sequencing

Yeast cells were grown in 50 ml of YPD to a concentration of >10^7^ cells/ml. The methods of library construction and Nanopore sequencing were described in [51]. Briefly, input DNA was treated with NEBNext FFPE RepairMix (M6630; NEB) to repair single-strand nicks, then DNA ends were repaired to form blunt ends using NEBNext End Repair Module (E6050; NEB). Samples were purified using AMPure XP beads. Each sample was barcoded using the Oxford Nanopore native barcoding genomic DNA kit (EXP- NBD104), followed by adapter ligation using ligation-based library kit (SQK-LSK109). After final purification, multiple barcoded libraries were loaded onto MinION flow cells (FLO-MIN106D R9.4.1) and analyzed on a MinION sequencer (MIN-101B).

The monitoring of the sequencing process, as well as real-time basecalling, were performed using Minknow (v21.02.1). The build-in basecaller (Guppy,4.3.2) was used to simultaneously convert raw electrical signals into fastq files. Output fastq files were aligned to S288c genome (sacCer3) using NGMLR [52]. The resulting.sam files were converted to.bam files using Samtools [45]. The sorted bam files were used to call structural variants by Sniffles [52] generating.vcf files. Both sorted bam file and.vcf file were uploaded to Ribbon [36] to visualize genomic structural variants.

### CHEF gel electrophoresis and Southern blot analysis

Intact chromosomal DNA was prepared by embedding yeast cells in plugs made of 0.8% low-melt agarose followed by the treatment of the plugs with Zymolase and Proteinase K as described in [9]. A Biorad CHEF Mapper XA system was used to perform the CHEF gel electrophoresis. The chromosomal DNA was transferred to nylon membrane for Southern analysis. The preparation of hybridization probes, and the conditions used for hybridization are described in our previous study [53].

### Data deposit

The raw data of SNP microarray is deposited as a GEO dataset with the accession number GSE205199. The raw sequencing data are available at the National Center for Biotechnology Information (NCBI) Sequence Read Archive (SRA) under BioProject PRJNA544995.

## Supporting information

S1 Fig Southern analysis of CRISPR/Cas9-induced DSBs within Ty1 elements. **(A)** Location of CRISPR/Cas9 target with respect to flanking *Xho*I sites. Most of the Ty1 elements have *Xho*I sites in the flanking delta elements. A cut within the CRISPR/Cas9 target would be expected to produce fragments of about 1.9 and 3.6 kb in *Xho*I-treated DNA. The location of the three hybridization probes used in the experiment are shown as short black lines within the Ty1. **(B)** Ethidium-bromide-stained gel of *Xho*I-treat DNA. DNA was isolated from cells grown in liquid medium containing 2% raffinose (R), or grown in 2% raffinose then harvested, washed, and grown in 2% raffinose plus 2% galactose (G) for four hours. The relevant genotypes (full genotypes in S1 Table) are: MD703 (diploid without plasmid), MD704 (diploid with pMD97), MD745 (haploid with pMD97), MD747 (haploid with control plasmid lacking the guide RNA). **(C)** Hybridization pattern of *XhoI*-treated samples. In both the haploid and diploid isolates with the pMD97 plasmid, bands of the sizes expected for cleavage at the CRISPR/Cas9 target are observed after galactose induction. There are also 3 kb bands in all of the samples likely to reflect cross-hybridization of the probes with DNA fragments derived from the 2-micron plasmid.
(TIF)

S2 Fig Location of Ty elements in strains S288c, W303-1A, and YJM789. Ty1 and Ty2 were indicated by orange and violet respectively, and gray ovals show the locations of centromeres. The direction of the arrows indicates whether the Ty element is annotated in the Watson or Crick orientations. The triangles with nicks represent incomplete Ty elements. The black, red and blue horizontal lines represent S288c, W303-1A and YJM789 chromosomes, respectively. Chromosomes are normalized to the same size with SGD coordinates shown above the top lines.
(TIF)

S3 Fig Recombination events that lead to T-LOH or I-LOH. Red and blue lines indicate different homologs and ovals/circles show centromeres. All events are initiated as a DSB on the red homolog. The mechanistic details of these events are outlined in Fig 2. **(A)** Reciprocal crossover associated with conversion. The region of conversion is shown within the horizontal rectangle. The products shown in the vertical rectangle are homozygous for the blue SNPs located distal to the exchange. **(B)** The same pattern of T-LOH shown in S3A Fig can be the result of a BIR event. **(C)** In this figure, the DSB is repaired by an interaction that is not associated with a crossover, leading to an I-LOH event.
(TIF)

S4 Fig Examples of different types of chromosome alterations as determined by genomic sequencing. As described in the legend to Fig 4, the Y-axis shows the Ratio of Coverage, the normalized numbers of reads for each SNP, and the X-axis shows SGD coordinates. **(A)** I-LOH event on XIII. **(B)** T-LOH event on XV. **(C)** T-DUP on V. **(D)** T-DEL on XIII. **(E)** I-DUP on XII. **(F)** I-DEL on XII.
(TIF)

S5 Fig Nanopore sequence analysis of a hybrid Ty elements created by recombination between *YPLWTy1-1* and *YPRCTy1-2*. The sequence shown is that of *YPRCTy1-2*. We did a BLAST analysis with the sequence of *YPRWTy1-1*, and the SNPs that distinguish these two Ty1 elements are highlighted in purple, green or black; the sequence of the target site for the guide RNA is shown in yellow. We examined 15 Nanopore “reads” that included the hybrid Ty element in isolate MD741-6. Those SNPs that matched the *YPRCTy1-2* sequence in at least two-thirds of the reads are shown in purple, and those that matched the *YPLWTy1-1* SNPs are shown in black. The SNPs shown in green matched the *YPLWTy1-2* SNP, but in less than two-thirds of the reads. As shown in the figure, the putative region of gene conversion extended to both sides of the guide RNA target.
(TIF)

S6 Fig Analysis of the breakpoints of Ty-Ty recombinants in MD741-6. **(A)** Based on microarray data and CHEF gel analysis, we predicted a recombination event between two Ty1 elements located on opposite arms of chromosome XVI (left arm of XVI and right arm of XVI shown in black and red, respectively). Such an exchange requires that one chromatid is looped relative to the other. One daughter cell (shown in a rectangle) would contain a duplication of the segment of XVI near the left end of the chromosome, and a deletion of sequences near the right end; this pattern of deletion/duplication was observed in MD741-6. Nanopore sequencing of MD741-6 (shown in S5 Fig) demonstrates the existence of a hybrid Ty element in which most of the element is derived from *YPRCTy1-2* with a smaller contribution from *YPLWTy1-1*. Notably, sequences from *YPRCTy1-2* are found flanking the guide RNA target site as indicated by the green arrow. **(B)** Predicted pattern of gene conversion events associated with BIR. The region of conversion is expected to be restricted to one side of the DSB, assuming that mismatch repair does not occur during the initial strand invasion. **(C)** Predicted pattern of gene conversion associated with double-strand break repair. For many of these events, the region of gene conversion will occur on both sides of the initiating DSB.
(TIF)

S7 Fig Chromosome rearrangements in MD741-3. **(A)** Whole-genome sequencing analysis of chromosomes II, XII, and XIII. Two T-DELs (near coordinates 328 kb on II and 372 kb on XIII) and two T-DUPs (601 kb on XII and 196 kb on XIII) were detected. There is also a T-LOH event on XII that partially obscures the T-DEL on XII. **(B)** CHEF gels and Southern blots verified two rearranged chromosomes (red arrows). The larger one was generated by the fusion of segments from XII (601 kb to the right end) and XIII (0 kb to 372 kb), while the smaller one consists of segments from II (0 kb to 328 kb) and XIII (0 kb to 196 kb). The probes used for Southern analysis were *SEA4* (on the left arm of II), *RIF2* (on the right arm of XII), and *PGA3* (on the left arm of XIII).
(TIF)

S8 Fig Chromosome rearrangements in MD741-5. **(A)** Microarray analysis of chromosomes I, II, IV, X, XIII, and XV. The variation of ratio of coverage (RC) indicates copy-number changes of W303-1A- (red) and YJM789 (blue)-derived SNPs. Chromosomes I, IV, and XV have T-DELs, whereas chromosomes II, X, and XIII have T-DUPs. **(B)** Based on the patterns of terminal duplications and deletions, as well as the sizes of novel chromosome bands, we calculated that the three translocations involved are I and II, XIII and XV, and IV and X (details in S1 Text). The left part of B. shows the ethidium-bromide-stained gel of the control and MD741-5 samples with red arrows indicating novel chromosome bands. The nature of the translocations was verified by Southern analysis using the following probes: *FLC2* (left arm of I), SEA4 (left arm of II), *SOR1* (right arm of X), *HPR1* (right arm of IV), *PGA3* (left arm of XIII) and *FRE5* (right arm of XV).
(TIF)

S9 Fig Detection of chromosome rearrangements in MD741-5 by Nanopore sequencing. **(A)** Chromosome I-II translocation. The numbers below the black line are coordinates in a contiguous sequence demonstrating the translocation. Based on the coordinates in SGD, the breakpoint on chromosome I was 166,162 (close to a Crick-oriented Ty1, S2 Data) and the breakpoint on chromosome II was 226, 954 (close to a Watson-oriented Ty1, S2 Data). These breakpoints are also consistent with the microarray data (S2 Data). **(B)** Chromosome IV deletion. Based on the Nanopore sequencing data, there is a deletion on chromosome IV between coordinates 489,024 and 513,669, the approximate location of two Crick-oriented Ty elements in the YJM789 homolog (Dataset S1.3 in S1 Data). The Ty1 elements are in the gap in the Nanopore sequencing read (bottom part of figure) since the elements are not present in the reference strain S288c.
(TIF)

S10 Fig Analysis of sub-clones derived from MD741-5. By genomic sequencing, we examined the original isolate of MD741-5 in addition to two sub-clones derived from the original isolate (MD741-5-S1 and MD741-5-S2). **(A)** Patterns of LOH on chromosome IV. On chromosome IV, all three strains had the same deletion near coordinate 500 kb. Both MD741-5 and MD741-5-S1 had a terminal deletion near coordinate 1100 kb. However, MD741-5-S2 had a terminal LOH event with a breakpoint at approximately the same position. **(B)** Strains MD741-5 and MD741-5-S1 had terminal duplications near coordinate 480 kb. In contrast, MD741-5-S2 had an I-LOH event near coordinate 670 kb.
(TIF)

S11 Fig Chromosome rearrangements in MD741-6. **(A)** Genomic sequence analysis of chromosomes XIII and XVI. T-DELs were evident on chromosomes XIII and XVI on one arm, and T-DUPs were evident on the opposite arm of the same chromosome. Based on the observed sizes of the novel chromosomes and the breakpoints on XIII and XVI, we hypothesized that the chromosome of 860 kb represented a recombination event between Ty elements on the left and right arms of XIII, and the 580 kb chromosome was a consequence of recombination between Ty elements on the left and right arms of XVI (additional details in S1 Text). **(B)** By Southern analysis, we found that the 860 kb chromosome hybridized strongly to a probe from the left arm of XVI (*PLC1*), and the 560 kb chromosome hybridized strongly to a probe from the left arm of XIII (*PGA3*).
(TIF)

S12 Fig Detection of chromosome rearrangements in MD741-6 by Nanopore sequencing. **(A)** Coupled duplication and deletion on chromosome XIII as a consequence of recombination between Ty elements located on the opposite arms of XIII. The breakpoints obtained from Nanopore sequencing were 202,221 (close to a Watson-oriented Ty at 202 kb, S2 Data) and 378,618 (close to a Crick-oriented Ty at 379 kb, S2 Data). **(B)** Coupled duplication and deletion on chromosome XVI as a consequence of recombination between Ty elements located on the opposite arms of XVI. The breakpoints obtained from Nanopore sequencing were 62,375 (close to a Watson-oriented Ty at 62 kb, S2 Data) and 810,564 (close to a Crick-oriented Ty at 810 kb, S2 Data).
(TIF)

S13 Fig Nanopore analysis of chromosome rearrangements in MD741-7. **(A)** Chromosome V-XIII translocation. The breakpoints of the translocation are 493,149 on V (close to a Crick-oriented Ty at 493 kb, S2 Data) and 748,223 (close to a Crick-oriented Ty at 748 kb, S2 Data) on XIII. **(B)** I-DEL on chromosome XII. The breakpoints of the deletion on XII are 599,053 (close to Crick-oriented Ty at 599 kb, S2 Data) and 688,178 (close to a Crick-oriented Ty at 688 kb).
(TIF)

S14 Fig Unbalanced translocations in MD741-8 and MD741-9. **(A)** From the genomic sequence analysis, isolate MD741-8 has a T-DEL event on chromosome II, and a T-DUP event on XIII. If these changes in gene dosage reflect the II-XIII translocation, the expected size of the translocation is about 510 kb, and a novel chromosome of this size is detectable by CHEF gel analysis (S14C Fig). **(B)** In MD741-9, chromosomes II and XVI represent T-DEL and T-DUP products, respectively. If these products reflect the II-XVI translocation event, the expected size of the translocation is about 700 kb, and a novel chromosome (marked with red arrows in S14C and S14D Fig) is observed at this position. **(C)** By Southern analysis, the 510 kb chromosome in MD741-8 hybridizes to probes derived from the left arm of II (*SEA4*) and the left arm of XIII (*PGA3*). The 700 kb chromosome in MD741-9 hybridizes to a probe from the left arm of II (*SEA4*). **(D)** By Southern analysis, the 700 kb chromosome hybridizes to a probe from the left arm of XVI (*PLC1*), confirming the II-XVI translocation in MD741-9.
(TIF)

S15 Fig Balanced translocation in MD741-9. In addition to a translocation between II and XVI that was detectable by changes in gene dosage (S14B Fig), MD741-9 had several novel chromosomes that did not result in changes in gene dosage; the novel chromosomes were the result of a balanced translocation between chromosomes III and IV. **(A)** Microarray analysis showing that chromosomes III and IV had no alterations in gene dosage. **(B)** CHEF gel analysis indicating the presence of two novel chromosomes, one about 1240 kb and one about 610 kb (marked with red arrows). Southern analysis showed that the 1240 kb chromosome had a portion of the sequences from the right arm of IV (*RAD34*, coordinate 1092 kb) and the right arm of III (*FEN2*, coordinate 171 kb). The 610 kb chromosome hybridized to sequences derived from the right arm of IV (*HIM1*, coordinate 1102 kb) and the right arm of III (*NPP1*, coordinate 164 kb), although the breakpoints for the two translocations were different. **(C)** Depiction of the recombination event producing the III-IV reciprocal translocation. Ty elements are marked with black arrows. Co-segregation of the two translocations would maintain a balanced gene dosage for chromosomes III and IV. **(D)** Confirmation of the IV-III translocation of the 1240 kb chromosome by PCR. Primers located at coordinate 1095 kb of IV and 170 kb of III were used in a PCR reaction with genomic DNA of MD741-9. A fragment of the expected size (about 10 kb) was observed in the experimental, but not the control strain.
(TIF)

S16 Fig Formation of a circular chromosome in MD741-18. **(A)** By genomic sequence analysis, the strain MD741-18 had T-DELs on both the left and right arms of chromosome XV, but no T-DUPs on other chromosomes. **(B)** Generation of a double deletion by recombination between Ty elements on the opposite arms of chromosome XV derived from the W303-1A homolog. The acentric linear product would be expected to be lost, generating the observed double deletion. Continuous and dotted lines indicate the sequences on chromosome XV near the translocation breakpoint, and black triangles show the involved Ty elements. **(C)** PCR analysis showing the band expected for the circular chromosome. Primers XV-A and XV-S (shown in S16B Fig) were used in the PCR reaction.
(TIF)

S17 Fig Nanopore analysis of chromosome rearrangements in MD704-2h-1. The I-DEL (coordinates 489–514 kb) on chromosome IV, evident by sequencing, has the same Ty-associated breakpoints as in MD741-5 (S9B Fig). The Ty elements that are at the breakpoints of the YJM789-derived homolog, but are absent in S288c are shown as a gap.
(TIF)

S18 Fig Patterns of LOH observed in MD704-4h-1. **(A)** By microarray analysis, this isolate has T-DEL events on chromosomes III and IV with breakpoints in Ty elements. As shown in S19 Fig, this pattern can be explained as a consequence of a crossover between the Ty elements on the right arms of chromosomes III (FS2 has an inverted pair of Ty elements near coordinates 169 kb) and IV (Ty element at coordinate 668 kb), followed by a non-disjunction event. **(B)** CHEF gel analysis demonstrating the III-IV translocation. By ethidium-bromide staining, there an incompletely visualized novel chromosome in MD704-4h-1 with a size of about 817 kb. This band hybridizes to a probe from the right arm of chromosome III (*PAT1* located at coordinate 250 kb) and the left arm of chromosome IV (*ADY3* at coordinate 26 kb). The expected size of this translocation is 817 kb.
(TIF)

S19 Fig Pathway for producing the double deletion observed in isolate MD704-4h-1. Chromosomes III and IV are shown as thick and thin lines, respectively, with the red color indicating W303-1A-derived homologs and the blue color indicating YJM789-derived homologs. The event begins with a crossover between a Watson-oriented Ty1 at coordinate 668 kb on IV and a Watson-oriented Ty1 on III in FS2 at 169 kb. The second step is a non-disjunction event in which both chromatids with *CEN3* sequences derived from W303-1A are segregated into one daughter cell. The daughter cell that is outlined in the black rectangle will have the pattern of LOH events observed in MD704-4h-1.
(TIF)

S20 Fig Patterns of chromosome rearrangements in MD704-4h-2. **(A)** Genomic sequencing analysis of gene dosages on chromosomes II and III. Chromosome II has multiple transitions between regions with different gene dosages, whereas chromosome III has a single T-DEL event. On chromosome II, S20A Fig does not show a small region of heterozygosity between coordinates 221–230 kb at the scale used in the figure. **(B)** Based on Dataset S2.3 in S2 Data, we depict the ratio of coverage for different segments of chromosome II and III with the breakpoints of transitions shown as SGD coordinates. Horizontal arrows indicate Ty elements. Red and blue lines show W303-1A and YJM789 sequences, respectively. Thick and thin lines represent chromosome II and III sequences, respectively. **(C)** Arrangement of chromosome segments in MD704-4h-2 that are consistent with the analysis in S20A and S20B. Recombination events that could produce these rearranged chromosomes are shown in S22 Fig.
(TIF)

S21 Fig Nanopore analysis of chromosome rearrangements in MD704-4h-2. **(A)** Inversion/pseudo-isochromosome rearrangement on II. Based on microarray data, we predicted an inversion on chromosome II with a breakpoint fusing coordinates 227 kb and 406 kb (S22C Fig and S2 Data). By Nanopore sequencing, the inversion involves similar breakpoints at 226,953 and 405,572. There are Ty1 elements at both breakpoints. **(B)** II-III translocation. As predicted by the microarrays, the Nanopore sequencing confirmed a translocation between coordinate 336,548 of II and 168,934 of III; Ty1 sequences are located at both breakpoints.
(TIF)

S22 Fig Pathway for generating chromosome rearrangements in MD704-4h-2. In this figure, we show one pathway that could produce the patterns of LOH and I-DELs observed in MD704-4h-2. Other homologous recombination events could likely produce the same patterns. Other observations in support of these chromosome rearrangements are described in S1 Text. W303-1A- and YJM789-derived homologs are shown as red and blue lines, respectively. Green arrows show the position of DSBs that initiate the events, and black arrows show the SGD coordinates at the breakpoints of the rearrangements (consistent with the depictions in S20 Fig). **(A)** Gene conversions and a crossover on chromosome II. One conversion event, unassociated with a crossover, is the result of a DSB near coordinate 230 kb on the W303-1A homolog with a conversion tract that extends through the centromere to coordinate 266 kb. A second DSB occurs in the Ty element near coordinate 328 kb. The repair of this DSB is associated with a conversion event that extends to coordinate 295 kb and a crossover between the two homologs. Ty elements are shown as short horizontal arrows. **(B)** Recombination between chromosomes II and III. A DSB in a Ty element located on the W303-1A homolog of chromosome III is repaired by a BIR event with a Ty1 element on one of the chromosome II homologs. The resulting translocation would be about 660 kb. Chromosome III and II are shown as a thin and thick lines, respectively. **(C)** Recombination between Ty elements located on opposite arms of chromosome II. A DSB within a Ty element located on the right arm of YJM789 homolog of II is repaired by a BIR event involving the Ty element located at 221 kb on the left arm of the W303-1A homolog of II. The resulting translocation would be 620 kb. Co-segregation of the chromatids marked with asterisks would produce the observed patterns of LOH.
(TIF)

S23 Fig Patterns of LOH and I-DELs observed in isolate MD704-4h-6P. **(A)** Chromosome III has a duplication of the sequences between Ty elements located in FS1 and FS2 in the W303-1A homolog. **(B)** Chromosome VII has a T-DEL with a breakpoint in a Ty located near coordinate 536 kb. **(C)** Chromosome XIII has a complex pattern of alterations (breakpoints of each transition in parentheses): 1. Triplication of W303-1A-derived sequences (from left end of chromosome to 184 kb), 2. One copy of W303-1A and YJM789 sequences (184–280 kb), 3. Two copies of YJM789-derived sequences and no copies of W303-1A-derived sequences (280–372 kb), and 4. One copy of YJM789-derived sequences and no copies of W303-1A-derived sequences (372 kb to the right end of chromosome).
(TIF)

S24 Fig Nanopore analysis of chromosome rearrangements in MD704-4h-6P. **(A)** Tripartite VII-III-XIII translocation. As shown in S23 and S25 Figs, the microarray experiments indicate formation of a tripartite translocation as the result of a DSB on chromosome VII being repaired by a BIR event involving chromosome III, followed by a dissociation and a re-invasion of chromosome XIII. All breakpoints occur at the positions of Ty elements. **(B)** Chromosome XIII-XIII pseudo-isochromosome. This event reflects a recombination event between chromosome XIII homologs involving a Crick-oriented Ty1 element on the right arm of XIII near coordinate 372 kb and a Watson-oriented Ty element on the left arm of XIII located near coordinate 184 kb (S23C Fig). **(C)** I-DEL on chromosome XV. The Nanopore sequencing confirms that the deletion on chromosome XV results from recombination between two Watson-oriented Ty1 elements located near coordinates 664 kb and 710 kb (S2 Data).
(TIF)

S25 Fig Pathway for generating the rearrangements observed in MD704-4h-6P. W303-1A and YJM789 homologs are drawn in red and blue, respectively; arrows show the location of Ty elements and ovals indicate centromeres. Chromosomes XIII and VII are shown as thick and thin lines, respectively. Chromosome III is shown as a dotted line. **(A)** Recombination events involving chromosome XIII. A reciprocal crossover occurs between the two homologs at a breakpoint near coordinate 280 kb. In a second event, a DSB within the YJM789 homolog at a Ty located at coordinate 372 kb is repaired by a BIR event involving an invasion of the end into a Ty element located at position 184 kb on the W303-1A homolog. The resulting chromosome is about 556 kb, and has sequences from the left arm of XIII on both ends. **(B)** Recombination between chromosomes VII and XIII. A break occurs on the W303-1A-derived VII at a Ty element located at 536 kb. The broken end is repaired by two consecutive BIR events, the first invading a Ty at 169 kb on chromosome III and replicating sequences to a non-allelic Ty at position 149 kb. The end is then extruded and invades a Ty on the left arm of chromosome XIII at coordinate 184 kb, performing a second BIR event. The resulting chromosome contains sequences derived from chromosomes III, VII, and XIII and is about 740 kb. The bottom part of this figure shows the chromosomes (marked with asterisks) that could segregate together to yield the observed microarray pattern.
(TIF)

S26 Fig Distance between centromere-distal Ty1s and recombination breakpoints. Chromosomes are shown as double-stranded DNA molecules with centromeres indicated as circles or ovals. Dotted lines indicate DNA synthesis during the BIR event. **(A)** A DSB in the Ty1 followed by degradation of the centromere-proximal broken end can produce a recombinant chromosome in which the breakpoint is displaced from the initiating DSB. **(B)** For spontaneous T-LOH events, we examined the distance between the recombination breakpoint and the nearest centromere-distal Ty1 (shown as a percentage of the total events) [12]. **(C)** For T-LOH events induced by CRISPR/Cas9, we show a bar graph comparable to that of S26B Fig.
(TIF)

S1 Data Positions of Ty elements in S288c, W303-1A, and YJM789. (XLSX)

S2 Data Summary of chromosome rearrangements in MD741 and MD704 isolates. (XLSX)

S3 Data Summary of changes in chromosome number in MD741 and MD704 isolates. (XLSX)

S4 Data Point mutations in strains expressing CRISPR/Cas9. (XLSX)

S1 Table Yeast strains used in this study. (DOCX)

S2 Table Oligonucleotide primers used in this study. (DOCX)

S3 Table Diagnosis of 5-FOA-resistant haploid isolates by PCR. (DOCX)

S1 Text Supplemental methods and discussions. (DOCX)
