# Supplementary figures and images for: Shuffling the yeast genome using CRISPR/Cas9-generated DSBs that target the transposable Ty1 elements
Source: PLoS Genet. 2023 Jan 26;19(1):e1010590. doi: 10.1371/journal.pgen.1010590 (PMC9879454; doi:10.1371/journal.pgen.1010590)

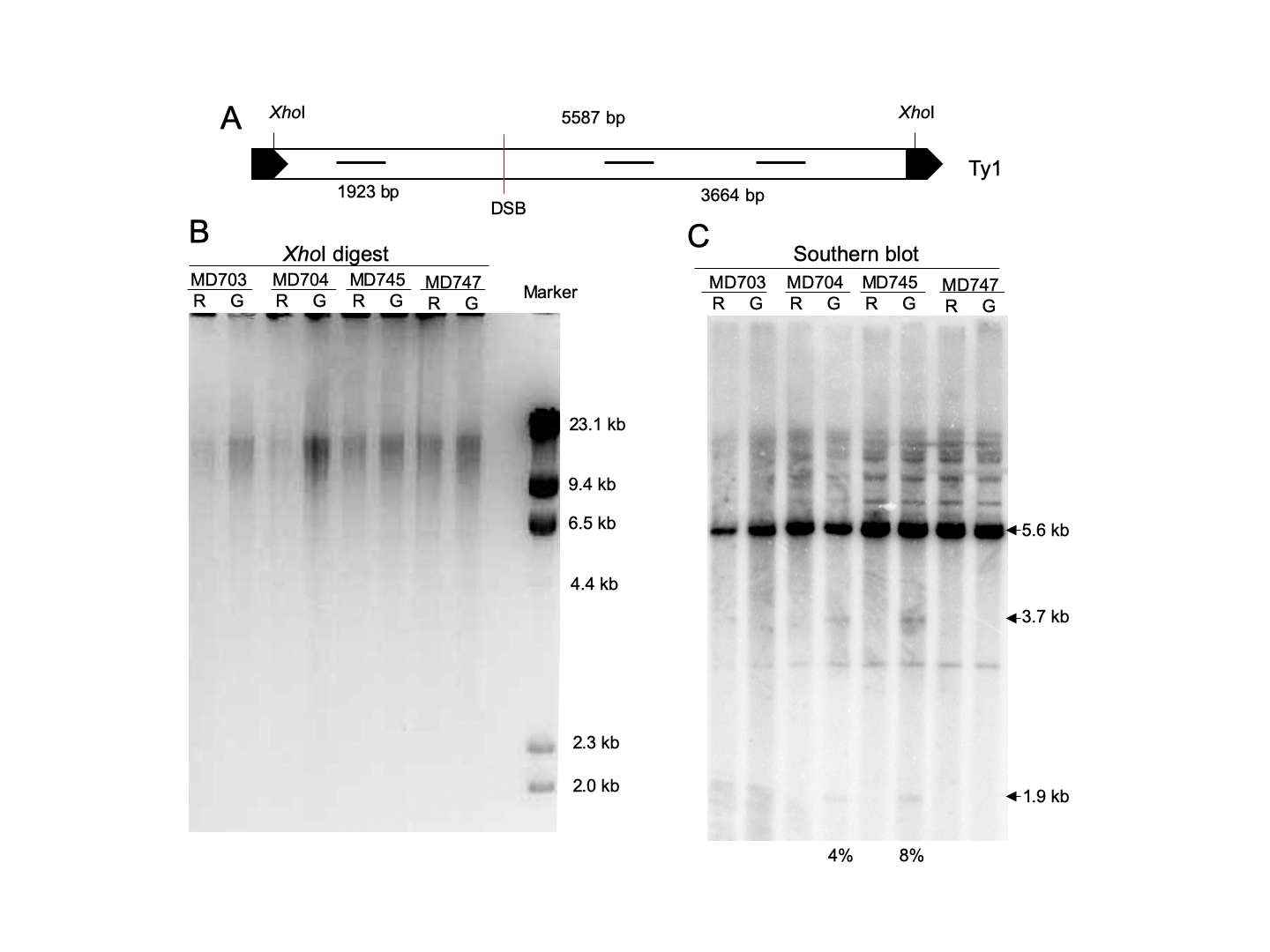

Supplement: S1 Fig — (A) Location of CRISPR/Cas9 target with respect to flanking XhoI sites. Most of the Ty1 elements have XhoI sites in the flanking delta elements. A cut within the CRISPR/Cas9 target would be expected to produce fragments of about 1.9 and 3.6 kb in XhoI-treated DNA. The location of the three hybridization probes used in the experiment are shown as short black lines within the Ty1. (B) Ethidium-bromide-stained gel of XhoI-treat DNA. DNA was isolated from cells grown in liquid medium containing 2% raffinose (R), or grown in 2% raffinose then harvested, washed, and grown in 2% raffinose plus 2% galactose (G) for four hours. The relevant genotypes (full genotypes in S1 Table) are: MD703 (diploid without plasmid), MD704 (diploid with pMD97), MD745 (haploid with pMD97), MD747 (haploid with control plasmid lacking the guide RNA). (C) Hybridization pattern of XhoI-treated samples. In both the haploid and diploid isolates with the pMD97 plasmid, bands of the sizes expected for cleavage at the CRISPR/Cas9 target are observed after galactose induction. There are also 3 kb bands in all of the samples likely to reflect cross-hybridization of the probes with DNA fragments derived from the 2-micron plasmid. (TIF) [file pgen.1010590.s001.tif]

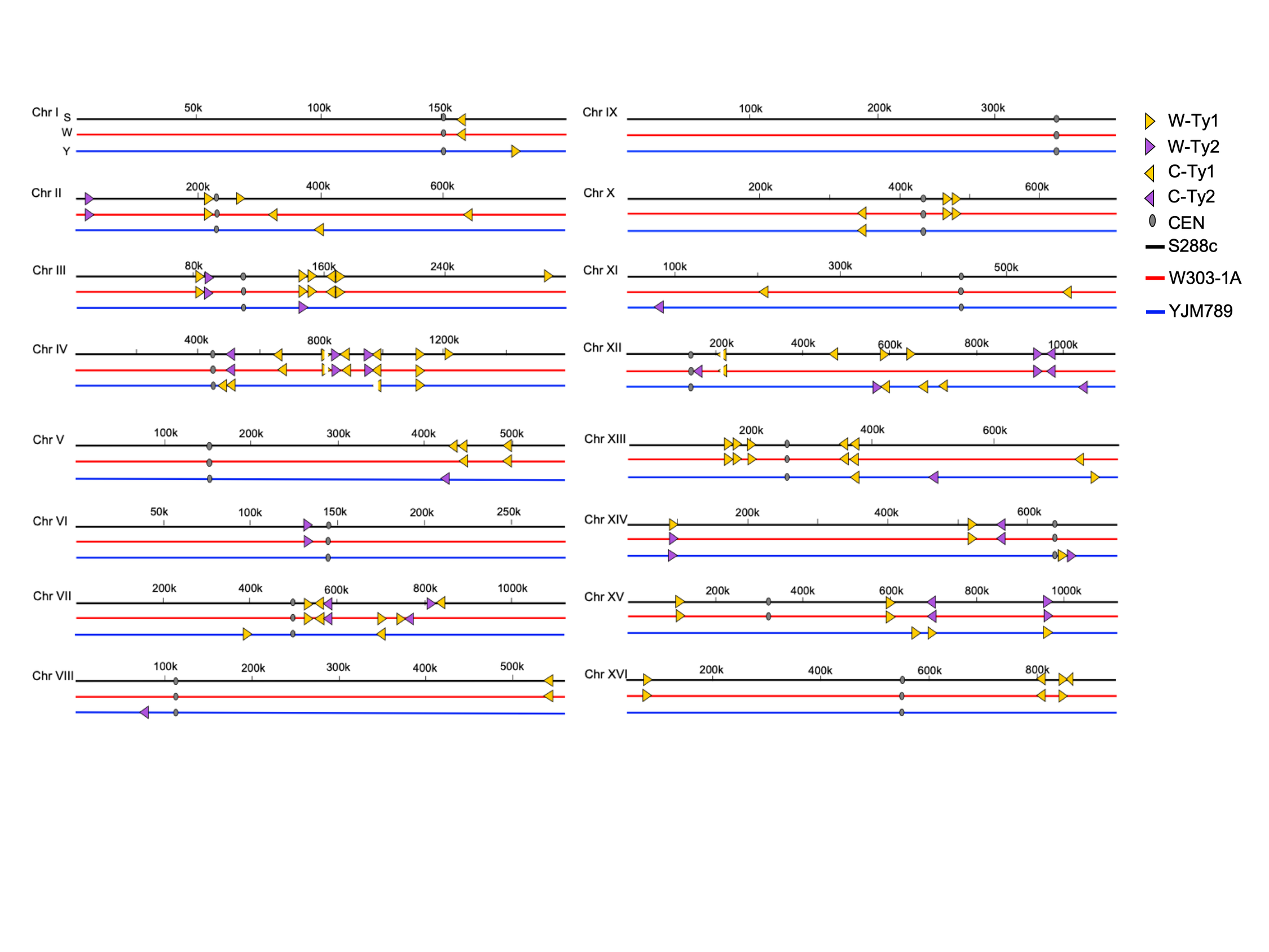

Supplement: S2 Fig — Ty1 and Ty2 were indicated by orange and violet respectively, and gray ovals show the locations of centromeres. The direction of the arrows indicates whether the Ty element is annotated in the Watson or Crick orientations. The triangles with nicks represent incomplete Ty elements. The black, red and blue horizontal lines represent S288c, W303-1A and YJM789 chromosomes, respectively. Chromosomes are normalized to the same size with SGD coordinates shown above the top lines. (TIF) [file pgen.1010590.s002.tif]

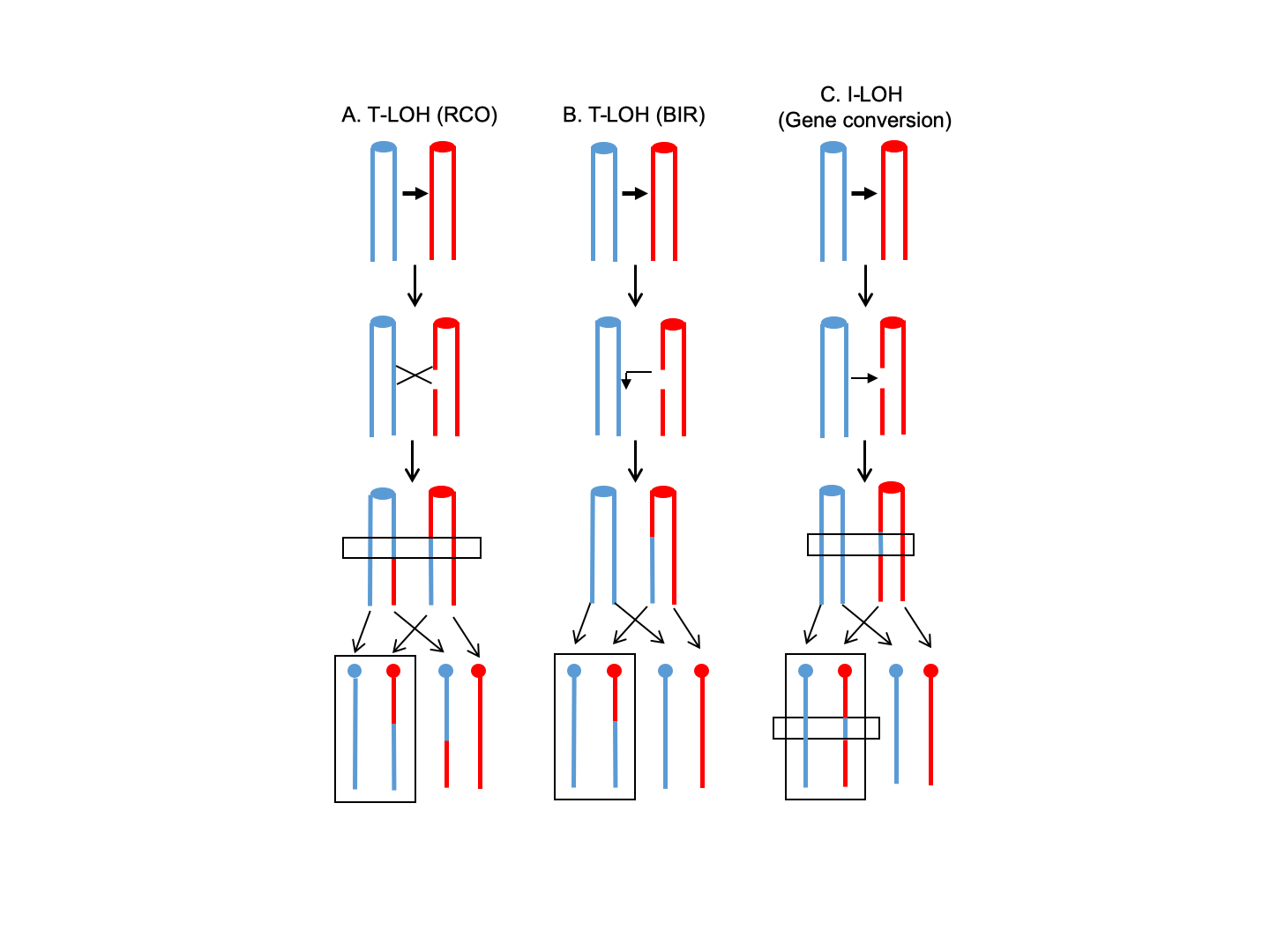

Supplement: S3 Fig — Red and blue lines indicate different homologs and ovals/circles show centromeres. All events are initiated as a DSB on the red homolog. The mechanistic details of these events are outlined in Fig 2. (A) Reciprocal crossover associated with conversion. The region of conversion is shown within the horizontal rectangle. The products shown in the vertical rectangle are homozygous for the blue SNPs located distal to the exchange. (B) The same pattern of T-LOH shown in S3A Fig can be the result of a BIR event. (C) In this figure, the DSB is repaired by an interaction that is not associated with a crossover, leading to an I-LOH event. (TIF) [file pgen.1010590.s003.tif]

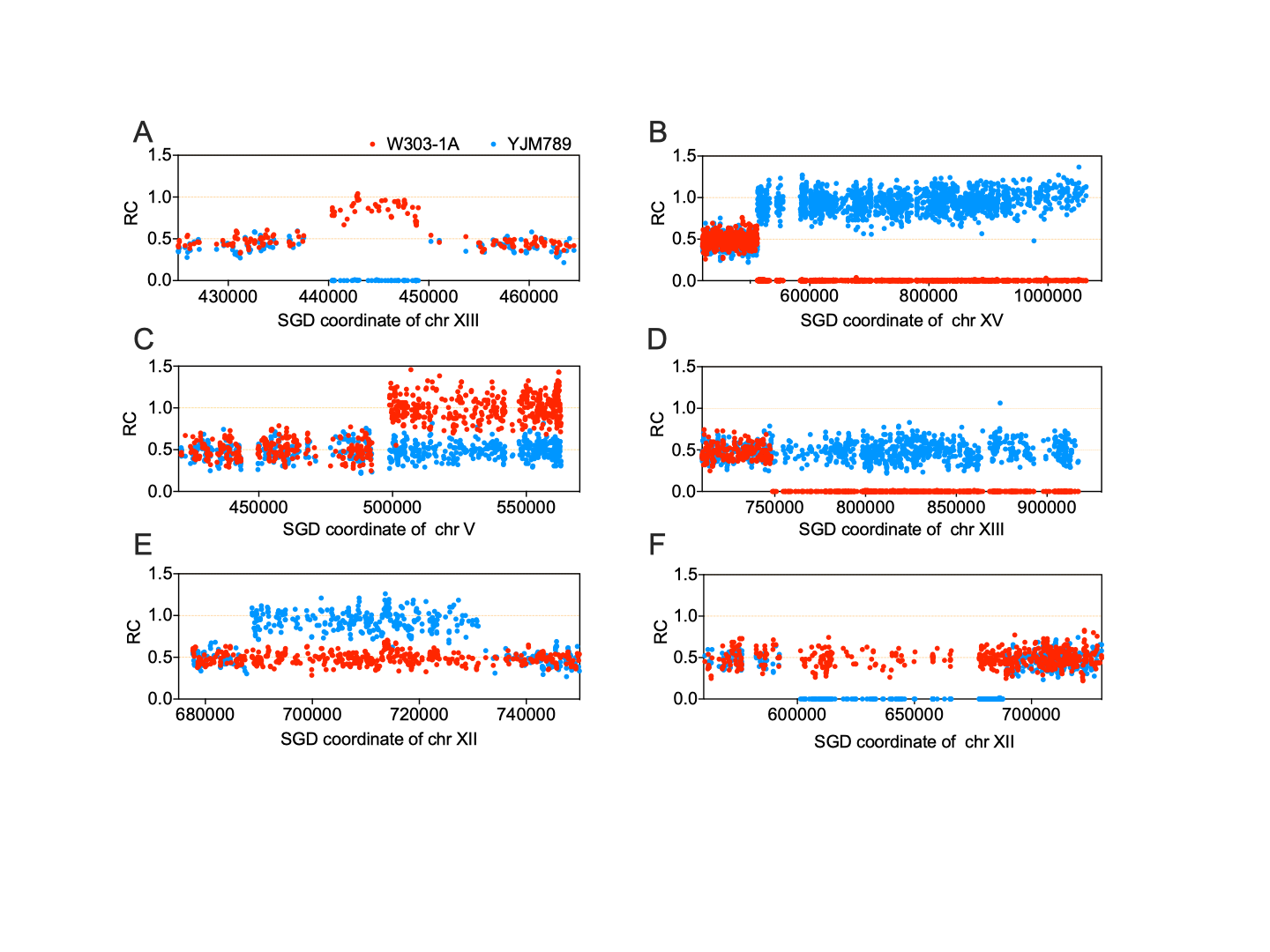

Supplement: S4 Fig — As described in the legend to Fig 4, the Y-axis shows the Ratio of Coverage, the normalized numbers of reads for each SNP, and the X-axis shows SGD coordinates. (A) I-LOH event on XIII. (B) T-LOH event on XV. (C) T-DUP on V. (D) T-DEL on XIII. (E) I-DUP on XII. (F) I-DEL on XII. (TIF) [file pgen.1010590.s004.tif]

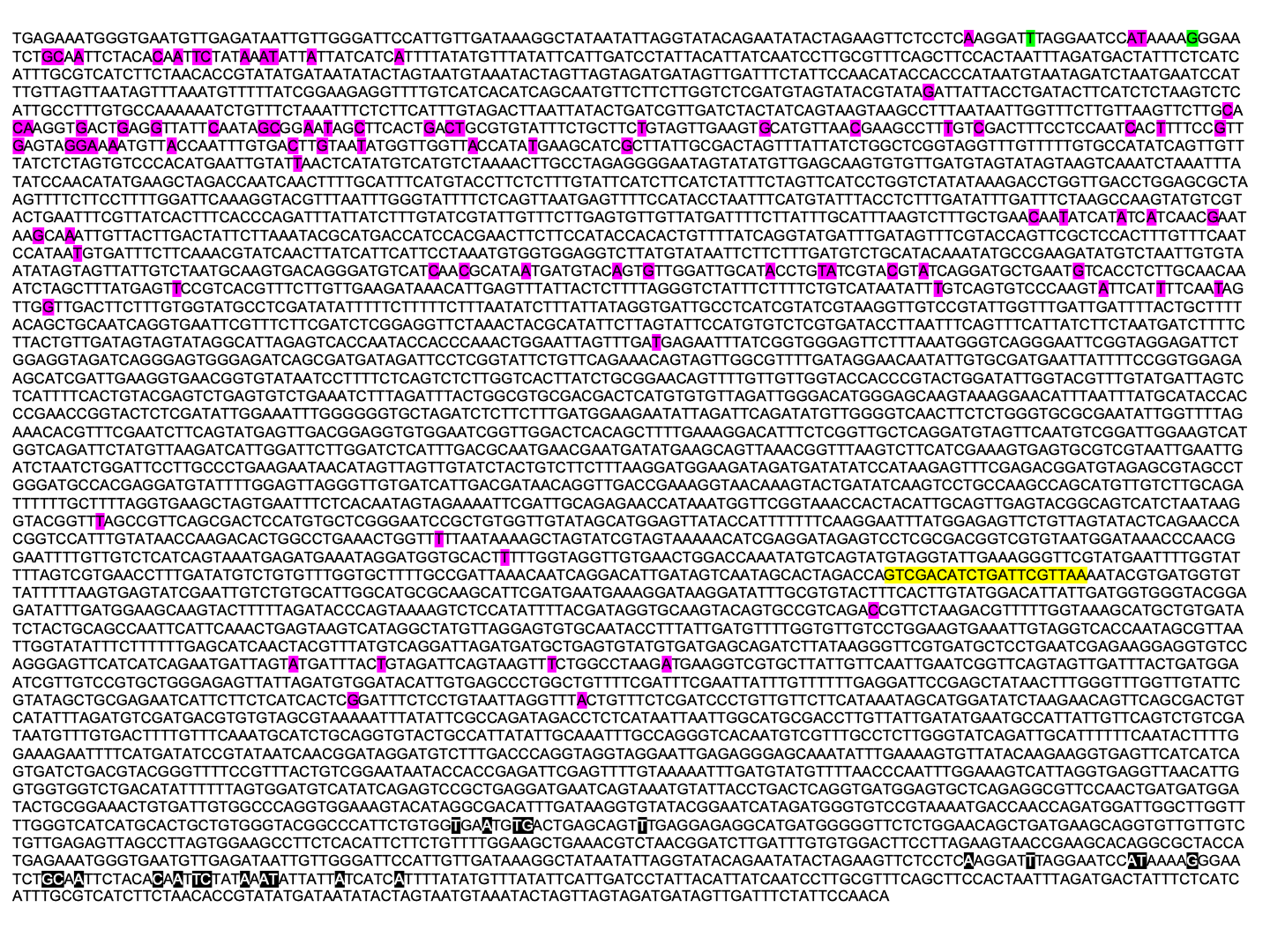

Supplement: S5 Fig — The sequence shown is that of YPRCTy1-2. We did a BLAST analysis with the sequence of YPRWTy1-1, and the SNPs that distinguish these two Ty1 elements are highlighted in purple, green or black; the sequence of the target site for the guide RNA is shown in yellow. We examined 15 Nanopore “reads” that included the hybrid Ty element in isolate MD741-6. Those SNPs that matched the YPRCTy1-2 sequence in at least two-thirds of the reads are shown in purple, and those that matched the YPLWTy1-1 SNPs are shown in black. The SNPs shown in green matched the YPLWTy1-2 SNP, but in less than two-thirds of the reads. As shown in the figure, the putative region of gene conversion extended to both sides of the guide RNA target. (TIF) [file pgen.1010590.s005.tif]

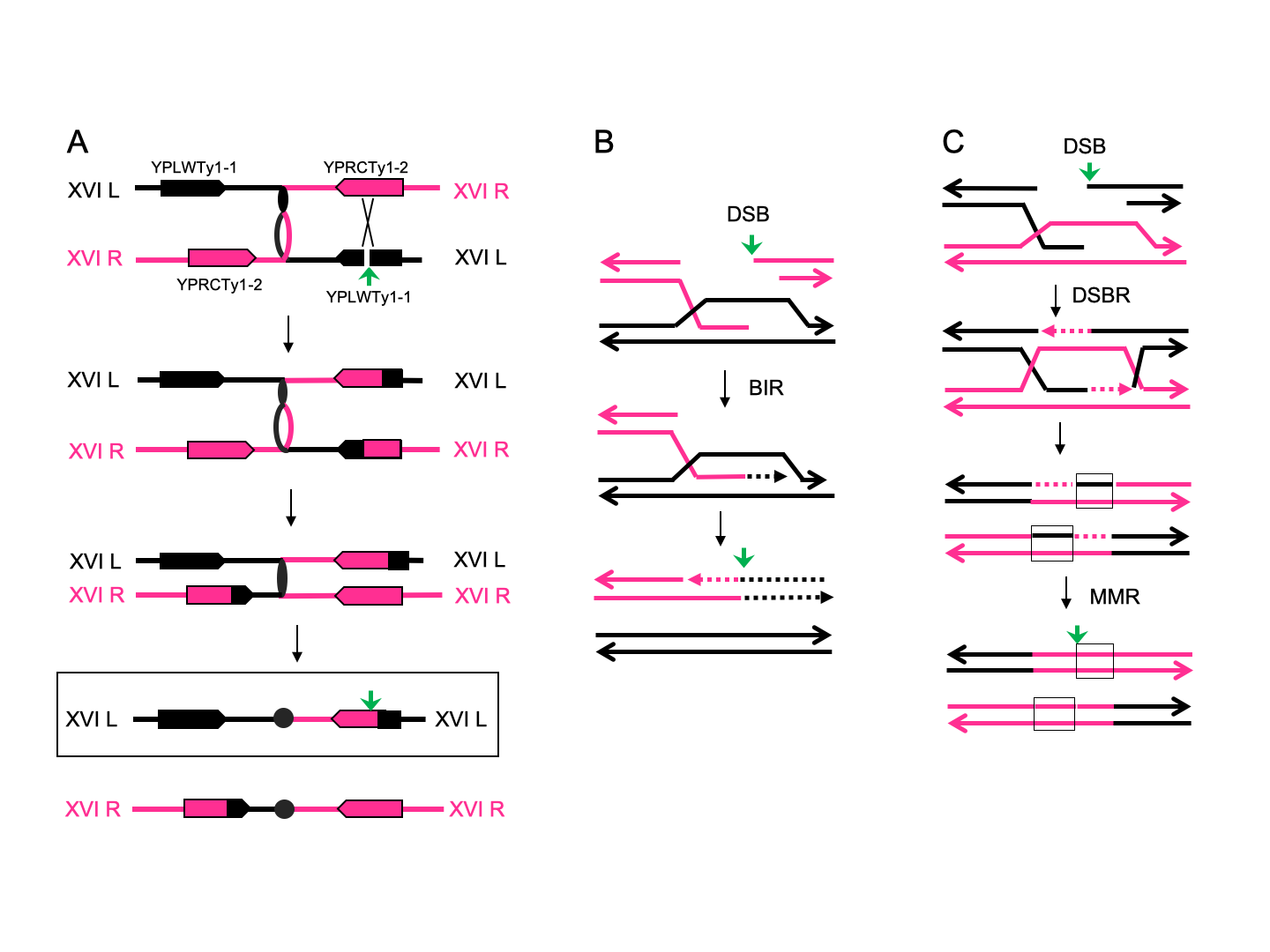

Supplement: S6 Fig — (A) Based on microarray data and CHEF gel analysis, we predicted a recombination event between two Ty1 elements located on opposite arms of chromosome XVI (left arm of XVI and right arm of XVI shown in black and red, respectively). Such an exchange requires that one chromatid is looped relative to the other. One daughter cell (shown in a rectangle) would contain a duplication of the segment of XVI near the left end of the chromosome, and a deletion of sequences near the right end; this pattern of deletion/duplication was observed in MD741-6. Nanopore sequencing of MD741-6 (shown in S5 Fig) demonstrates the existence of a hybrid Ty element in which most of the element is derived from YPRCTy1-2 with a smaller contribution from YPLWTy1-1. Notably, sequences from YPRCTy1-2 are found flanking the guide RNA target site as indicated by the green arrow. (B) Predicted pattern of gene conversion events associated with BIR. The region of conversion is expected to be restricted to one side of the DSB, assuming that mismatch repair does not occur during the initial strand invasion. (C) Predicted pattern of gene conversion associated with double-strand break repair. For many of these events, the region of gene conversion will occur on both sides of the initiating DSB. (TIF) [file pgen.1010590.s006.tif]

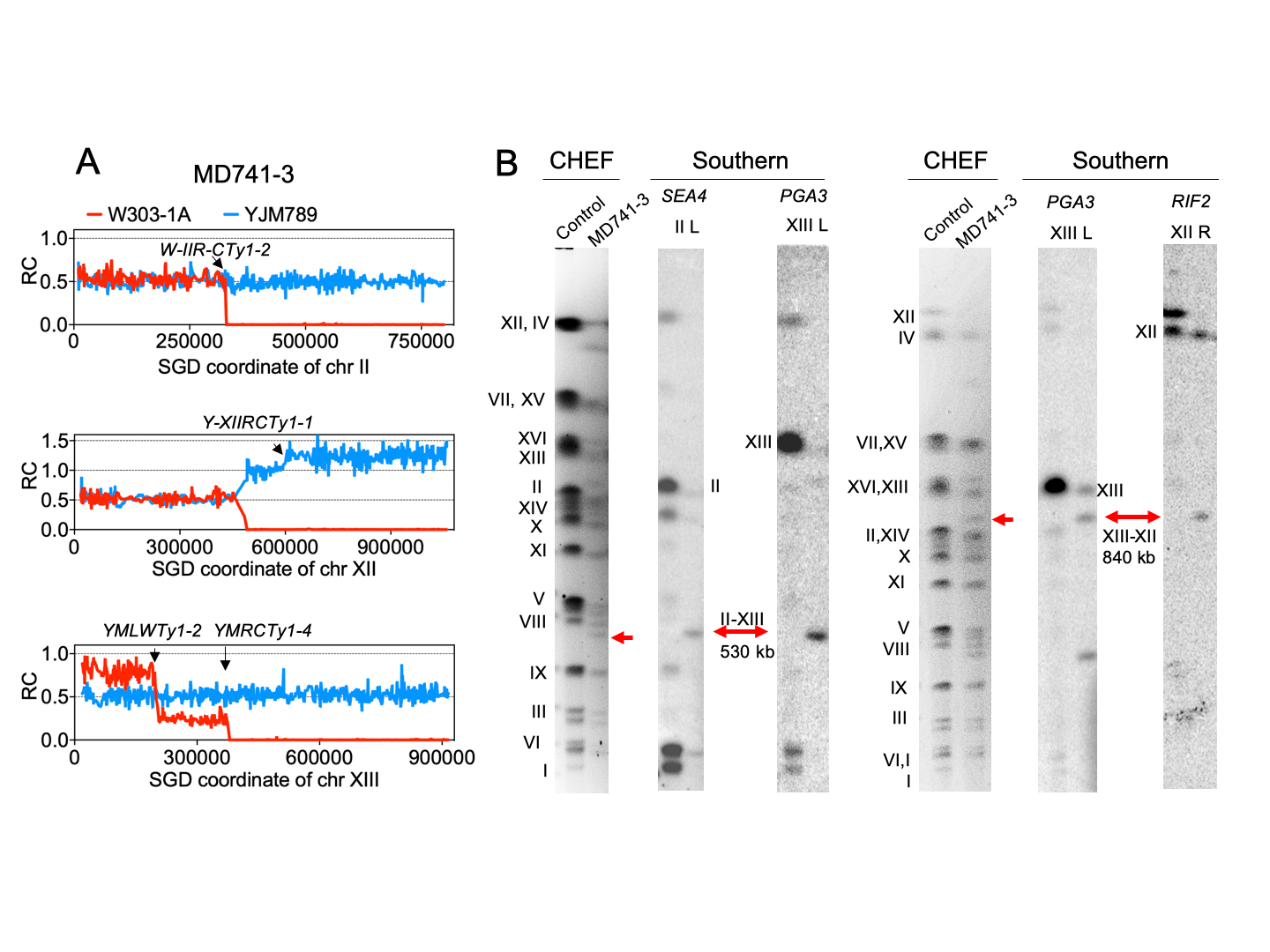

Supplement: S7 Fig — (A) Whole-genome sequencing analysis of chromosomes II, XII, and XIII. Two T-DELs (near coordinates 328 kb on II and 372 kb on XIII) and two T-DUPs (601 kb on XII and 196 kb on XIII) were detected. There is also a T-LOH event on XII that partially obscures the T-DEL on XII. (B) CHEF gels and Southern blots verified two rearranged chromosomes (red arrows). The larger one was generated by the fusion of segments from XII (601 kb to the right end) and XIII (0 kb to 372 kb), while the smaller one consists of segments from II (0 kb to 328 kb) and XIII (0 kb to 196 kb). The probes used for Southern analysis were SEA4 (on the left arm of II), RIF2 (on the right arm of XII), and PGA3 (on the left arm of XIII). (TIF) [file pgen.1010590.s007.tif]

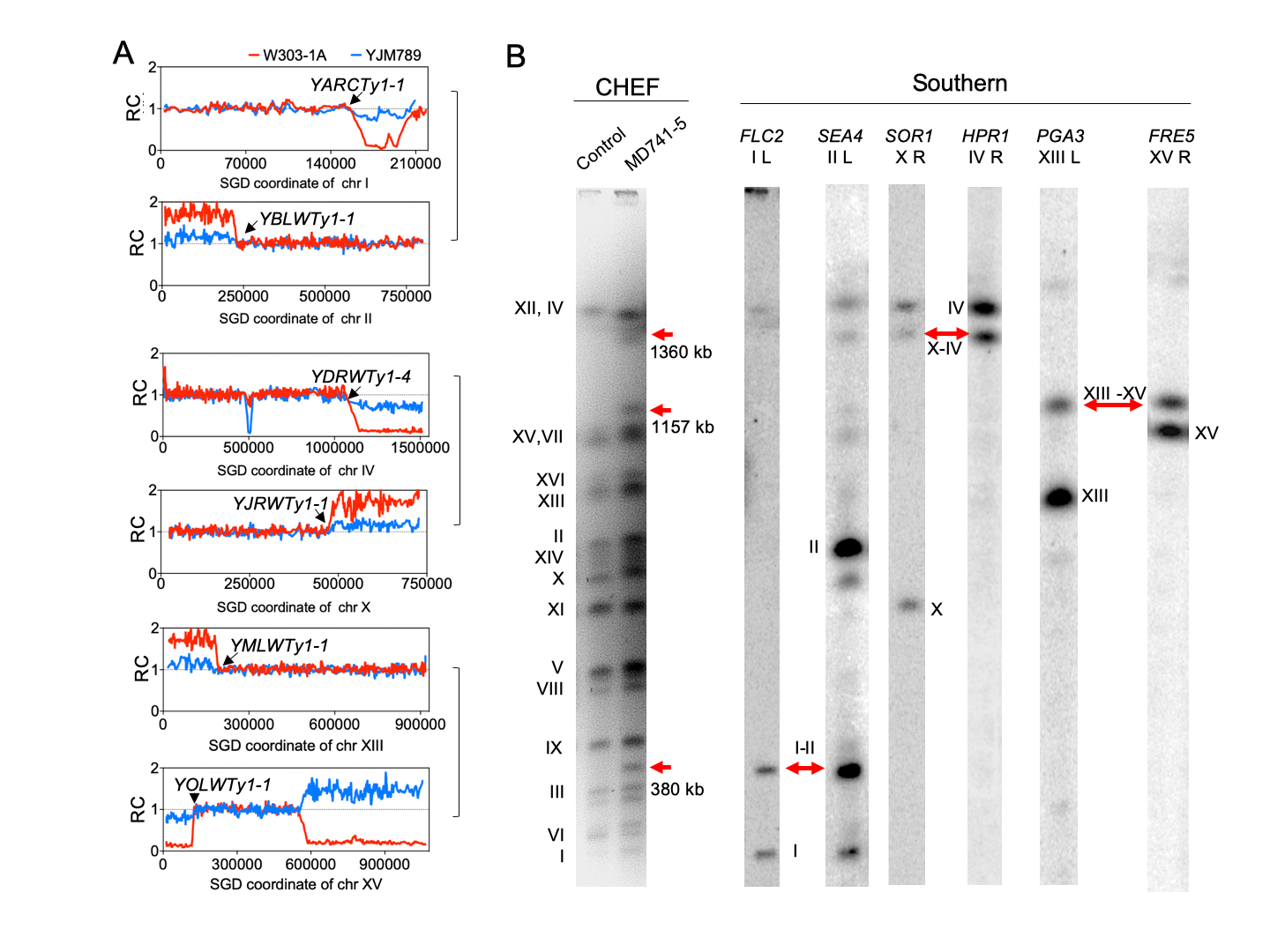

Supplement: S8 Fig — (A) Microarray analysis of chromosomes I, II, IV, X, XIII, and XV. The variation of ratio of coverage (RC) indicates copy-number changes of W303-1A- (red) and YJM789 (blue)-derived SNPs. Chromosomes I, IV, and XV have T-DELs, whereas chromosomes II, X, and XIII have T-DUPs. (B) Based on the patterns of terminal duplications and deletions, as well as the sizes of novel chromosome bands, we calculated that the three translocations involved are I and II, XIII and XV, and IV and X (details in S1 Text). The left part of B. shows the ethidium-bromide-stained gel of the control and MD741-5 samples with red arrows indicating novel chromosome bands. The nature of the translocations was verified by Southern analysis using the following probes: FLC2 (left arm of I), SEA4 (left arm of II), SOR1 (right arm of X), HPR1 (right arm of IV), PGA3 (left arm of XIII) and FRE5 (right arm of XV). (TIF) [file pgen.1010590.s008.tif]

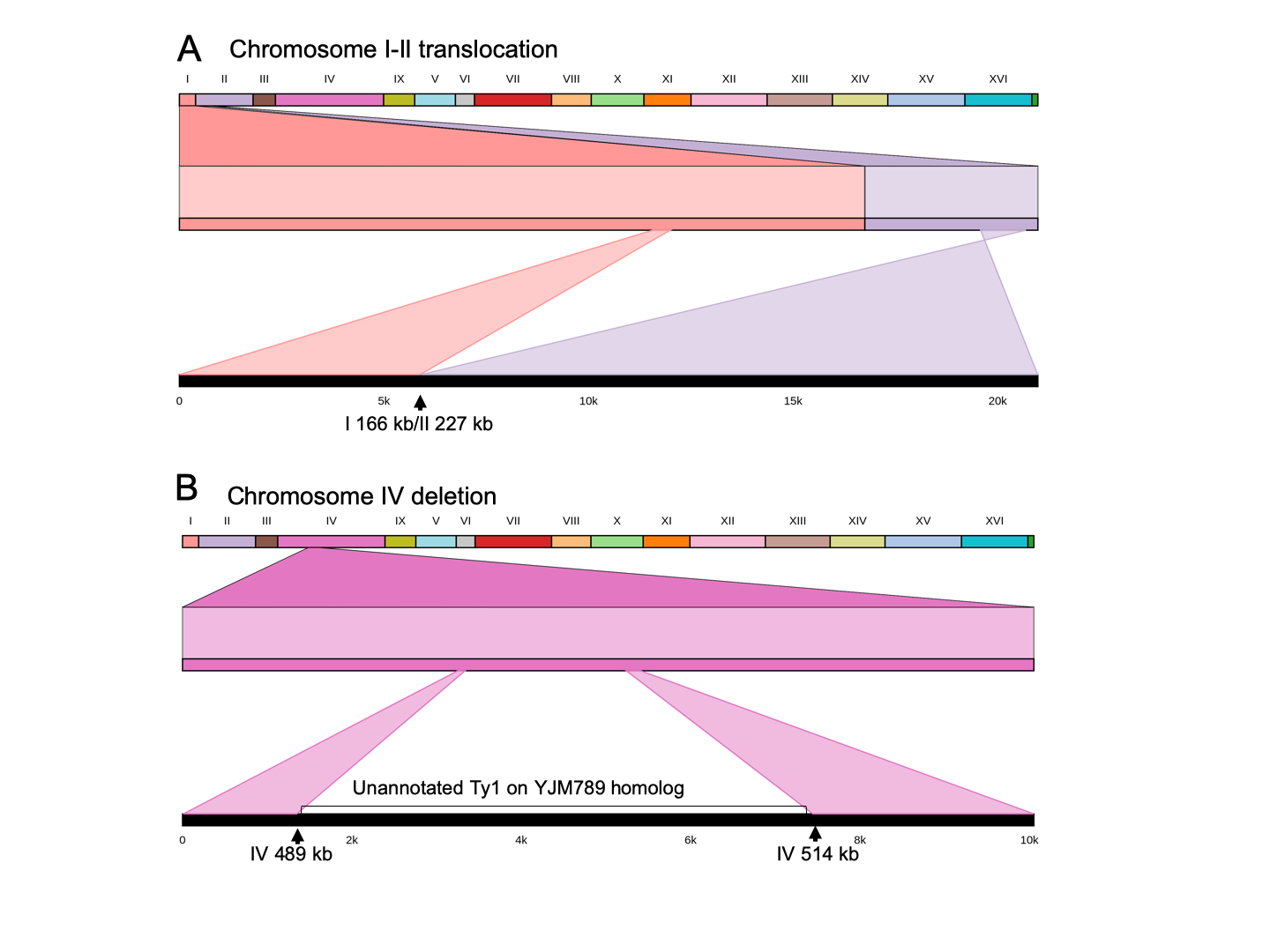

Supplement: S9 Fig — (A) Chromosome I-II translocation. The numbers below the black line are coordinates in a contiguous sequence demonstrating the translocation. Based on the coordinates in SGD, the breakpoint on chromosome I was 166,162 (close to a Crick-oriented Ty1, S2 Data) and the breakpoint on chromosome II was 226, 954 (close to a Watson-oriented Ty1, S2 Data). These breakpoints are also consistent with the microarray data (S2 Data). (B) Chromosome IV deletion. Based on the Nanopore sequencing data, there is a deletion on chromosome IV between coordinates 489,024 and 513,669, the approximate location of two Crick-oriented Ty elements in the YJM789 homolog (Dataset S1.3 in S1 Data). The Ty1 elements are in the gap in the Nanopore sequencing read (bottom part of figure) since the elements are not present in the reference strain S288c. (TIF) [file pgen.1010590.s009.tif]

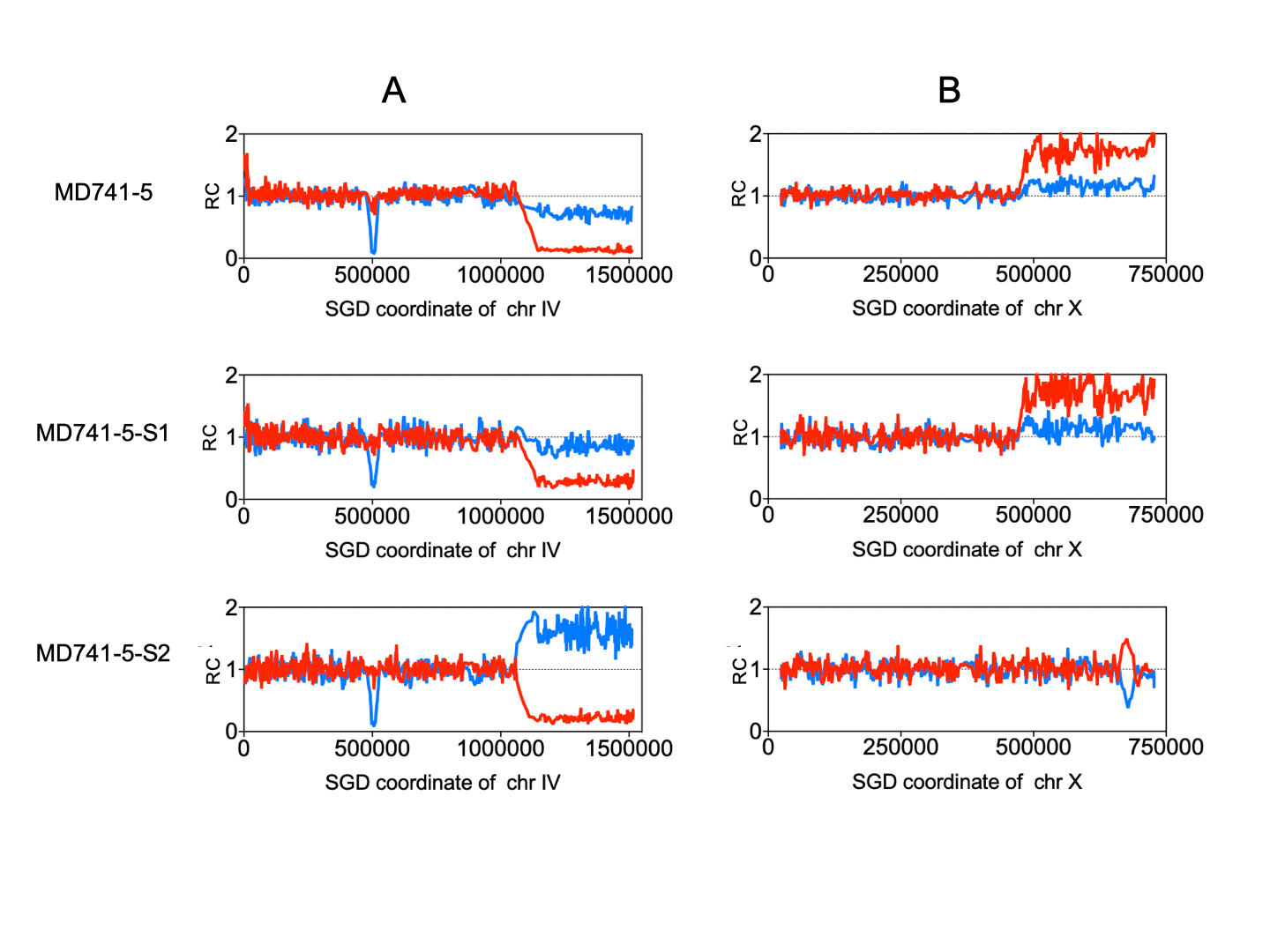

Supplement: S10 Fig — By genomic sequencing, we examined the original isolate of MD741-5 in addition to two sub-clones derived from the original isolate (MD741-5-S1 and MD741-5-S2). (A) Patterns of LOH on chromosome IV. On chromosome IV, all three strains had the same deletion near coordinate 500 kb. Both MD741-5 and MD741-5-S1 had a terminal deletion near coordinate 1100 kb. However, MD741-5-S2 had a terminal LOH event with a breakpoint at approximately the same position. (B) Strains MD741-5 and MD741-5-S1 had terminal duplications near coordinate 480 kb. In contrast, MD741-5-S2 had an I-LOH event near coordinate 670 kb. (TIF) [file pgen.1010590.s010.tif]

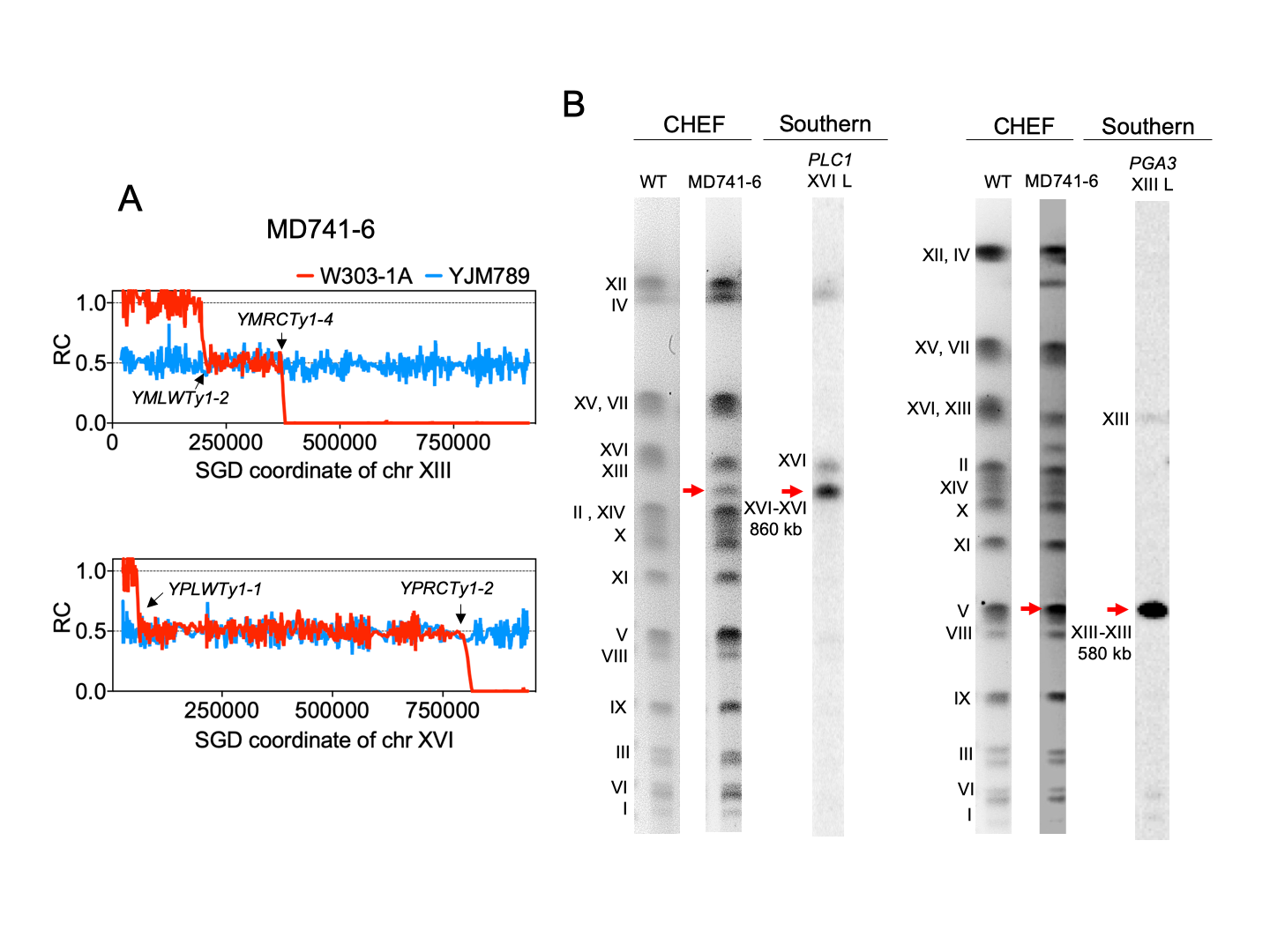

Supplement: S11 Fig — (A) Genomic sequence analysis of chromosomes XIII and XVI. T-DELs were evident on chromosomes XIII and XVI on one arm, and T-DUPs were evident on the opposite arm of the same chromosome. Based on the observed sizes of the novel chromosomes and the breakpoints on XIII and XVI, we hypothesized that the chromosome of 860 kb represented a recombination event between Ty elements on the left and right arms of XIII, and the 580 kb chromosome was a consequence of recombination between Ty elements on the left and right arms of XVI (additional details in S1 Text). (B) By Southern analysis, we found that the 860 kb chromosome hybridized strongly to a probe from the left arm of XVI (PLC1), and the 560 kb chromosome hybridized strongly to a probe from the left arm of XIII (PGA3). (TIF) [file pgen.1010590.s011.tif]

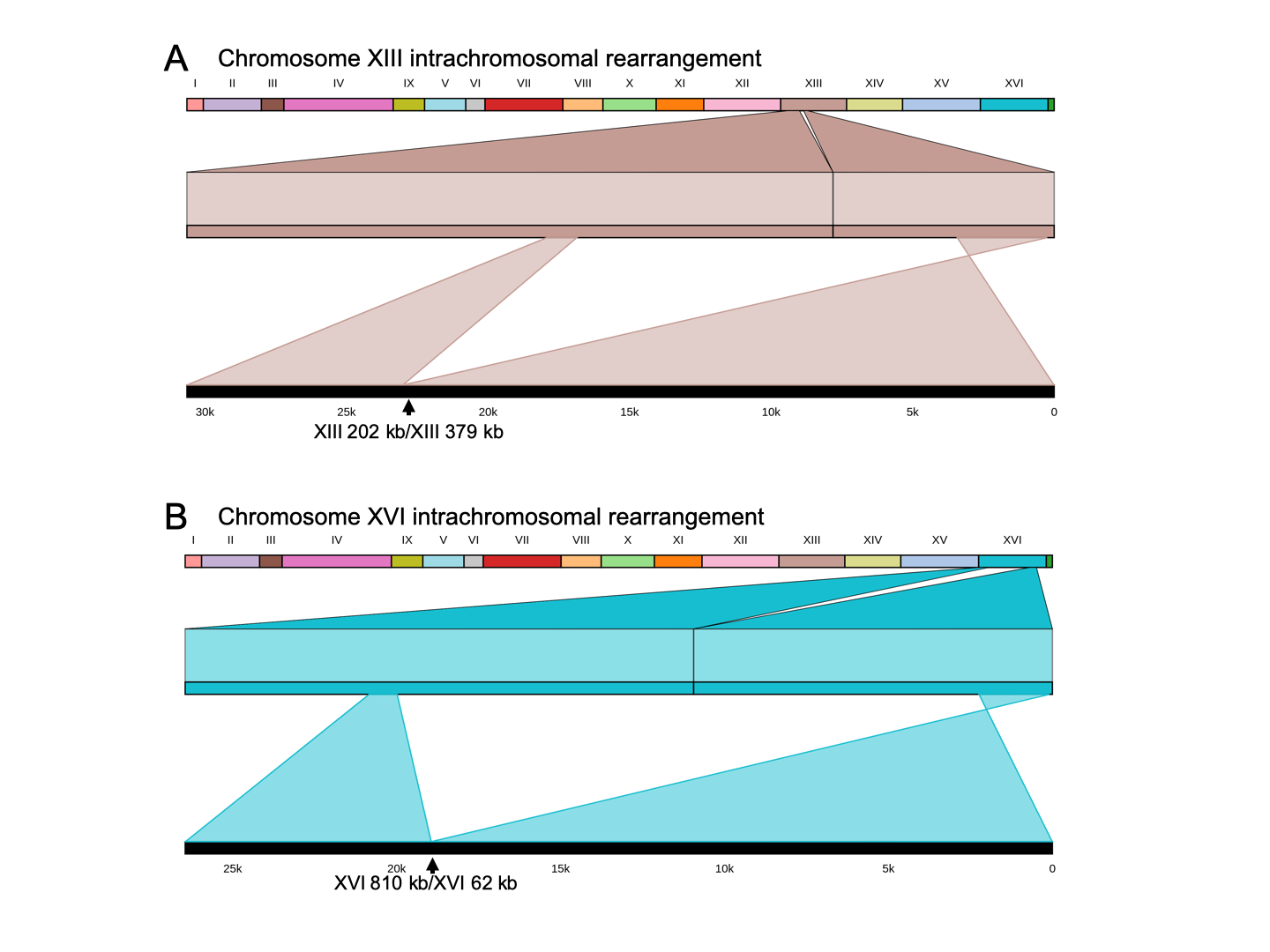

Supplement: S12 Fig — (A) Coupled duplication and deletion on chromosome XIII as a consequence of recombination between Ty elements located on the opposite arms of XIII. The breakpoints obtained from Nanopore sequencing were 202,221 (close to a Watson-oriented Ty at 202 kb, S2 Data) and 378,618 (close to a Crick-oriented Ty at 379 kb, S2 Data). (B) Coupled duplication and deletion on chromosome XVI as a consequence of recombination between Ty elements located on the opposite arms of XVI. The breakpoints obtained from Nanopore sequencing were 62,375 (close to a Watson-oriented Ty at 62 kb, S2 Data) and 810,564 (close to a Crick-oriented Ty at 810 kb, S2 Data). (TIF) [file pgen.1010590.s012.tif]

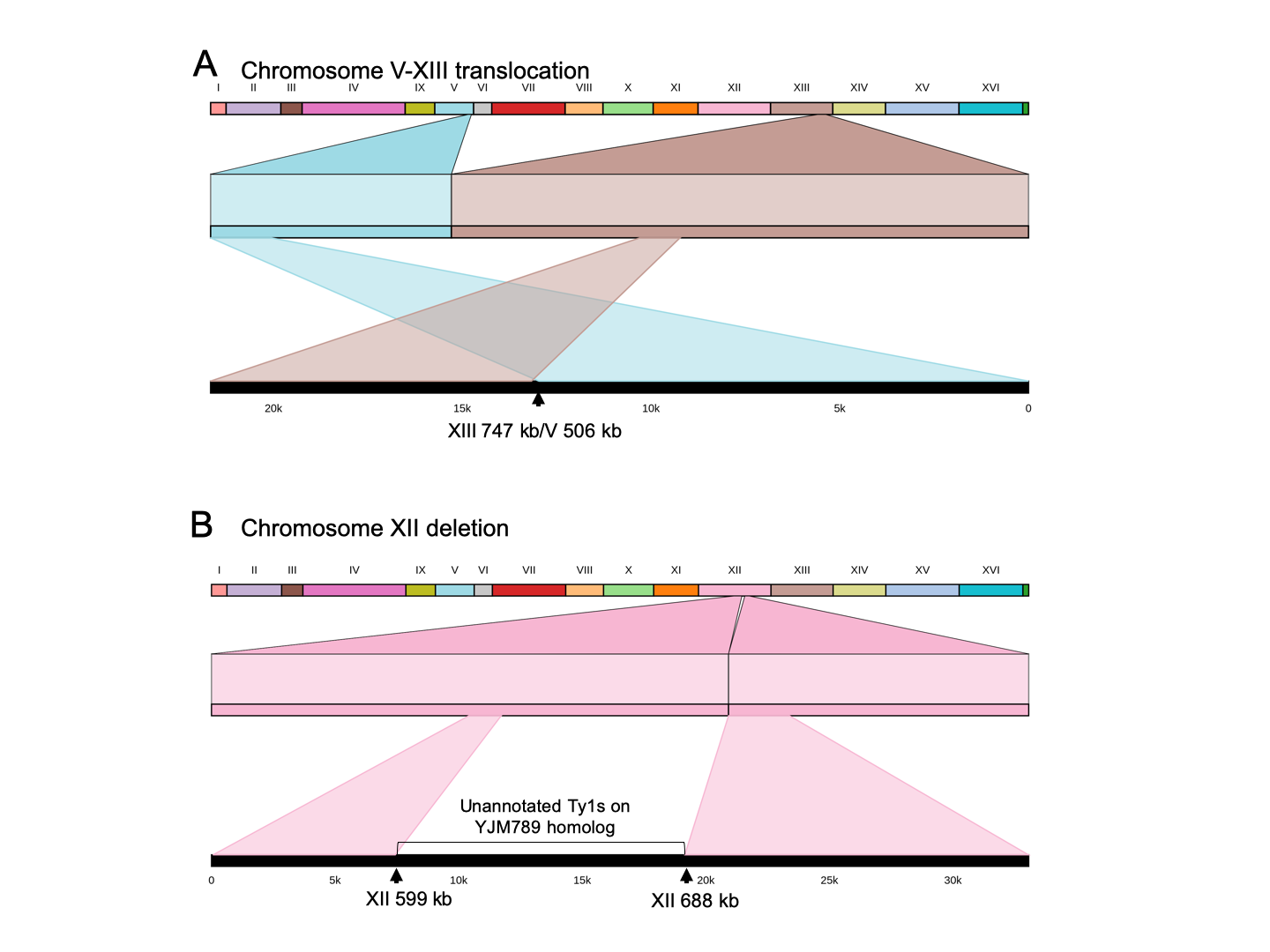

Supplement: S13 Fig — (A) Chromosome V-XIII translocation. The breakpoints of the translocation are 493,149 on V (close to a Crick-oriented Ty at 493 kb, S2 Data) and 748,223 (close to a Crick-oriented Ty at 748 kb, S2 Data) on XIII. (B) I-DEL on chromosome XII. The breakpoints of the deletion on XII are 599,053 (close to Crick-oriented Ty at 599 kb, S2 Data) and 688,178 (close to a Crick-oriented Ty at 688 kb). (TIF) [file pgen.1010590.s013.tif]

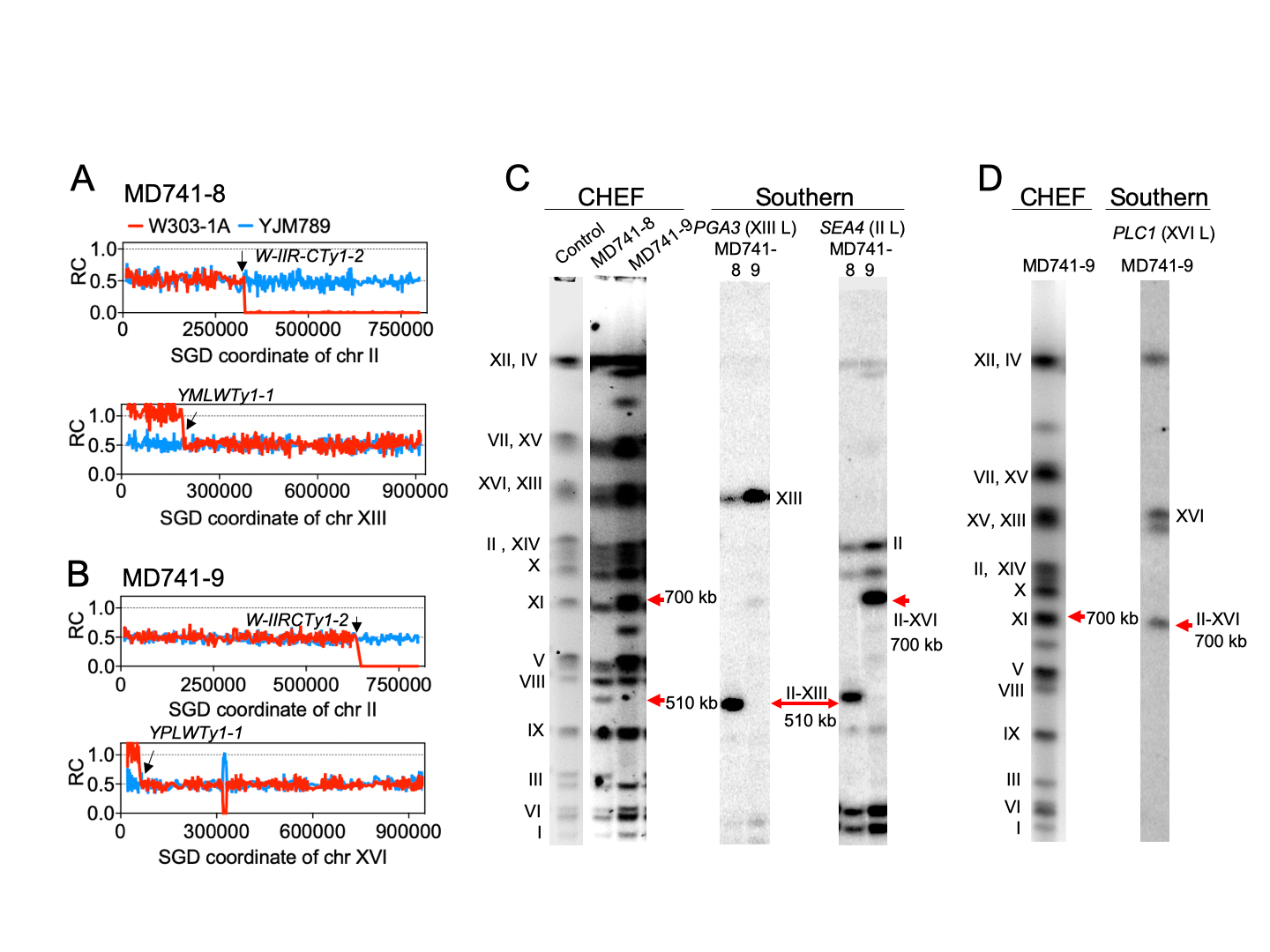

Supplement: S14 Fig — (A) From the genomic sequence analysis, isolate MD741-8 has a T-DEL event on chromosome II, and a T-DUP event on XIII. If these changes in gene dosage reflect the II-XIII translocation, the expected size of the translocation is about 510 kb, and a novel chromosome of this size is detectable by CHEF gel analysis (S14C Fig). (B) In MD741-9, chromosomes II and XVI represent T-DEL and T-DUP products, respectively. If these products reflect the II-XVI translocation event, the expected size of the translocation is about 700 kb, and a novel chromosome (marked with red arrows in S14C and S14D Fig) is observed at this position. (C) By Southern analysis, the 510 kb chromosome in MD741-8 hybridizes to probes derived from the left arm of II (SEA4) and the left arm of XIII (PGA3). The 700 kb chromosome in MD741-9 hybridizes to a probe from the left arm of II (SEA4). (D) By Southern analysis, the 700 kb chromosome hybridizes to a probe from the left arm of XVI (PLC1), confirming the II-XVI translocation in MD741-9. (TIF) [file pgen.1010590.s014.tif]

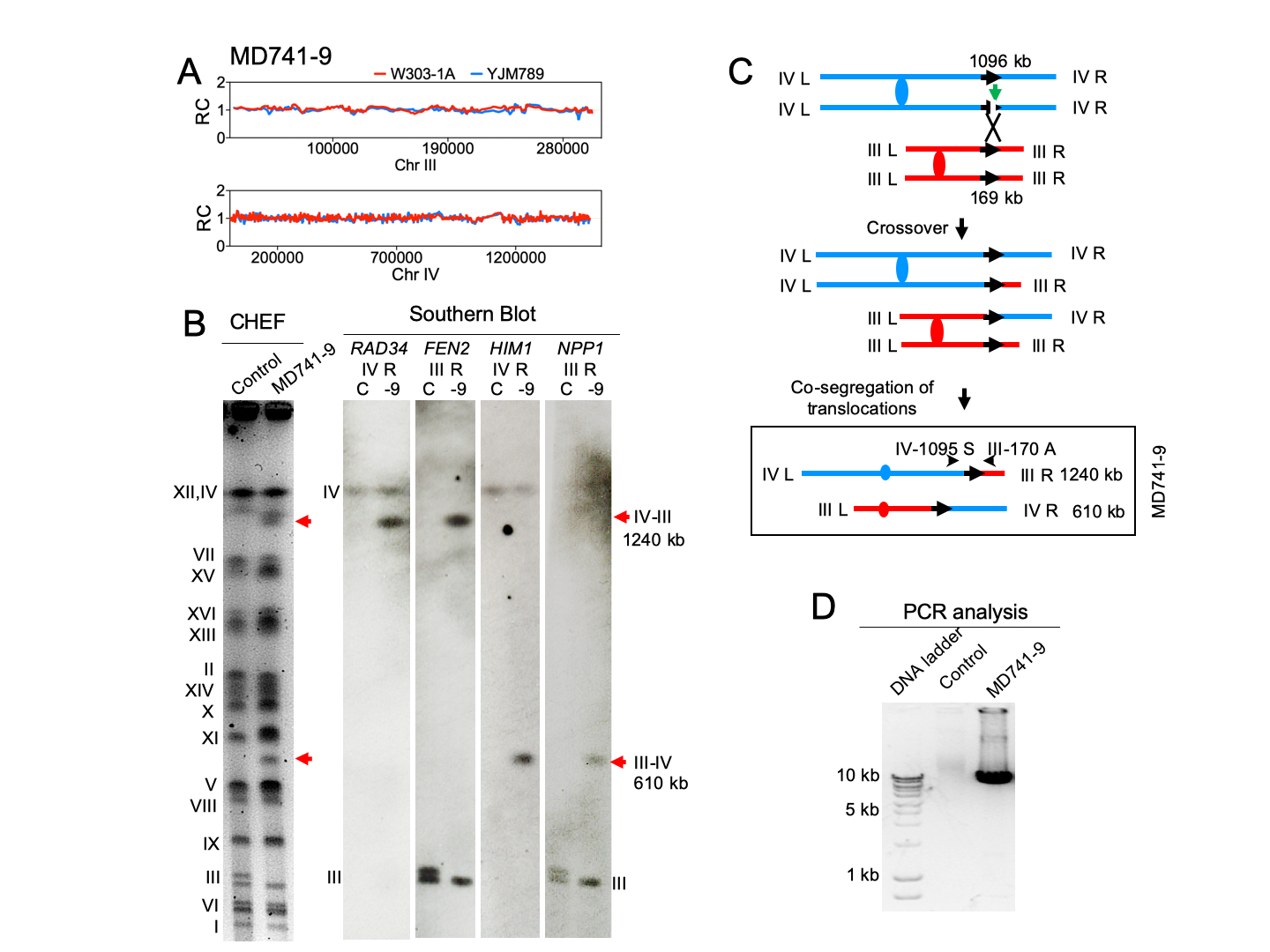

Supplement: S15 Fig — In addition to a translocation between II and XVI that was detectable by changes in gene dosage (S14B Fig), MD741-9 had several novel chromosomes that did not result in changes in gene dosage; the novel chromosomes were the result of a balanced translocation between chromosomes III and IV. (A) Microarray analysis showing that chromosomes III and IV had no alterations in gene dosage. (B) CHEF gel analysis indicating the presence of two novel chromosomes, one about 1240 kb and one about 610 kb (marked with red arrows). Southern analysis showed that the 1240 kb chromosome had a portion of the sequences from the right arm of IV (RAD34, coordinate 1092 kb) and the right arm of III (FEN2, coordinate 171 kb). The 610 kb chromosome hybridized to sequences derived from the right arm of IV (HIM1, coordinate 1102 kb) and the right arm of III (NPP1, coordinate 164 kb), although the breakpoints for the two translocations were different. (C) Depiction of the recombination event producing the III-IV reciprocal translocation. Ty elements are marked with black arrows. Co-segregation of the two translocations would maintain a balanced gene dosage for chromosomes III and IV. (D) Confirmation of the IV-III translocation of the 1240 kb chromosome by PCR. Primers located at coordinate 1095 kb of IV and 170 kb of III were used in a PCR reaction with genomic DNA of MD741-9. A fragment of the expected size (about 10 kb) was observed in the experimental, but not the control strain. (TIF) [file pgen.1010590.s015.tif]

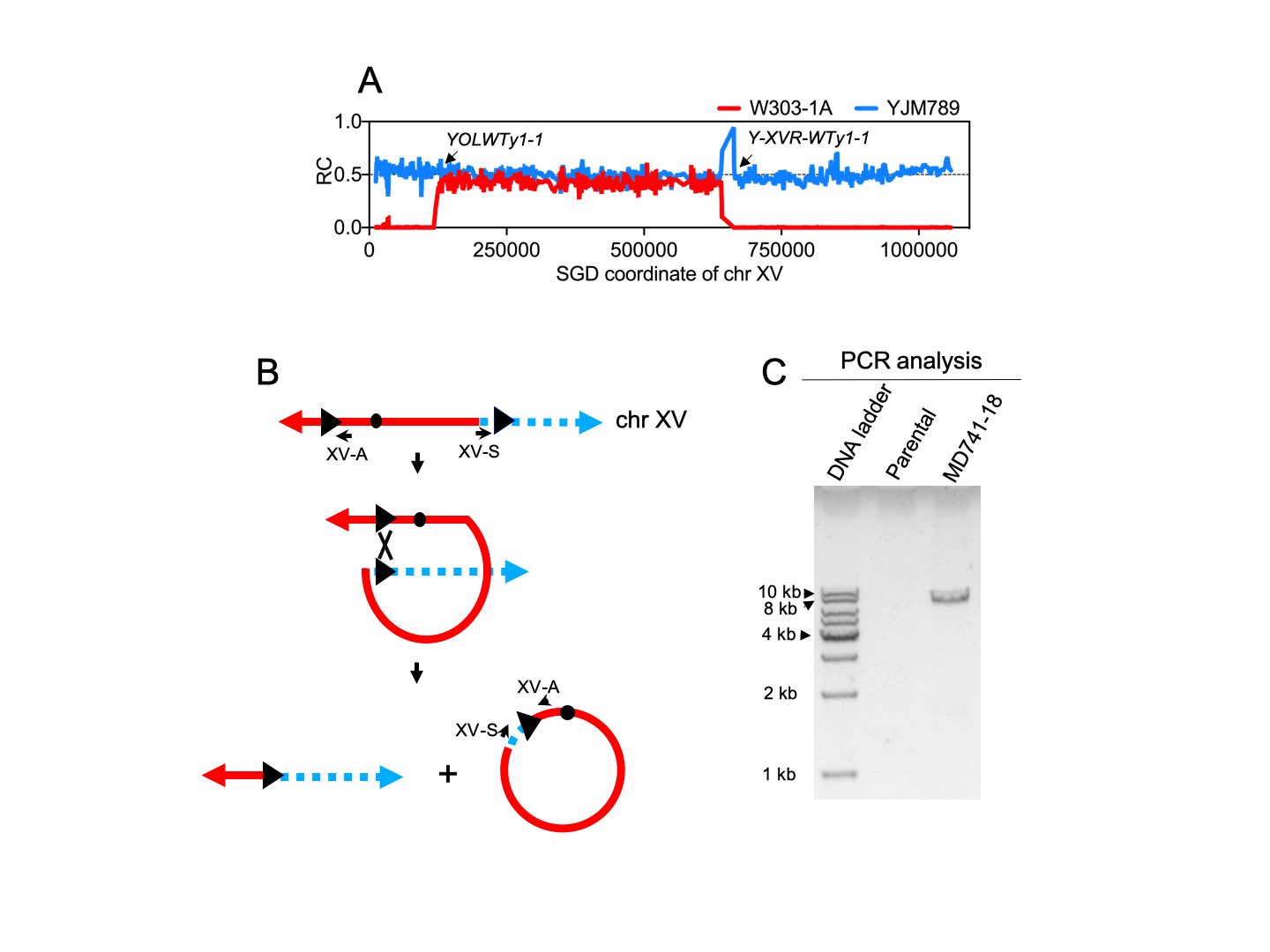

Supplement: S16 Fig — (A) By genomic sequence analysis, the strain MD741-18 had T-DELs on both the left and right arms of chromosome XV, but no T-DUPs on other chromosomes. (B) Generation of a double deletion by recombination between Ty elements on the opposite arms of chromosome XV derived from the W303-1A homolog. The acentric linear product would be expected to be lost, generating the observed double deletion. Continuous and dotted lines indicate the sequences on chromosome XV near the translocation breakpoint, and black triangles show the involved Ty elements. (C) PCR analysis showing the band expected for the circular chromosome. Primers XV-A and XV-S (shown in S16B Fig) were used in the PCR reaction. (TIF) [file pgen.1010590.s016.tif]

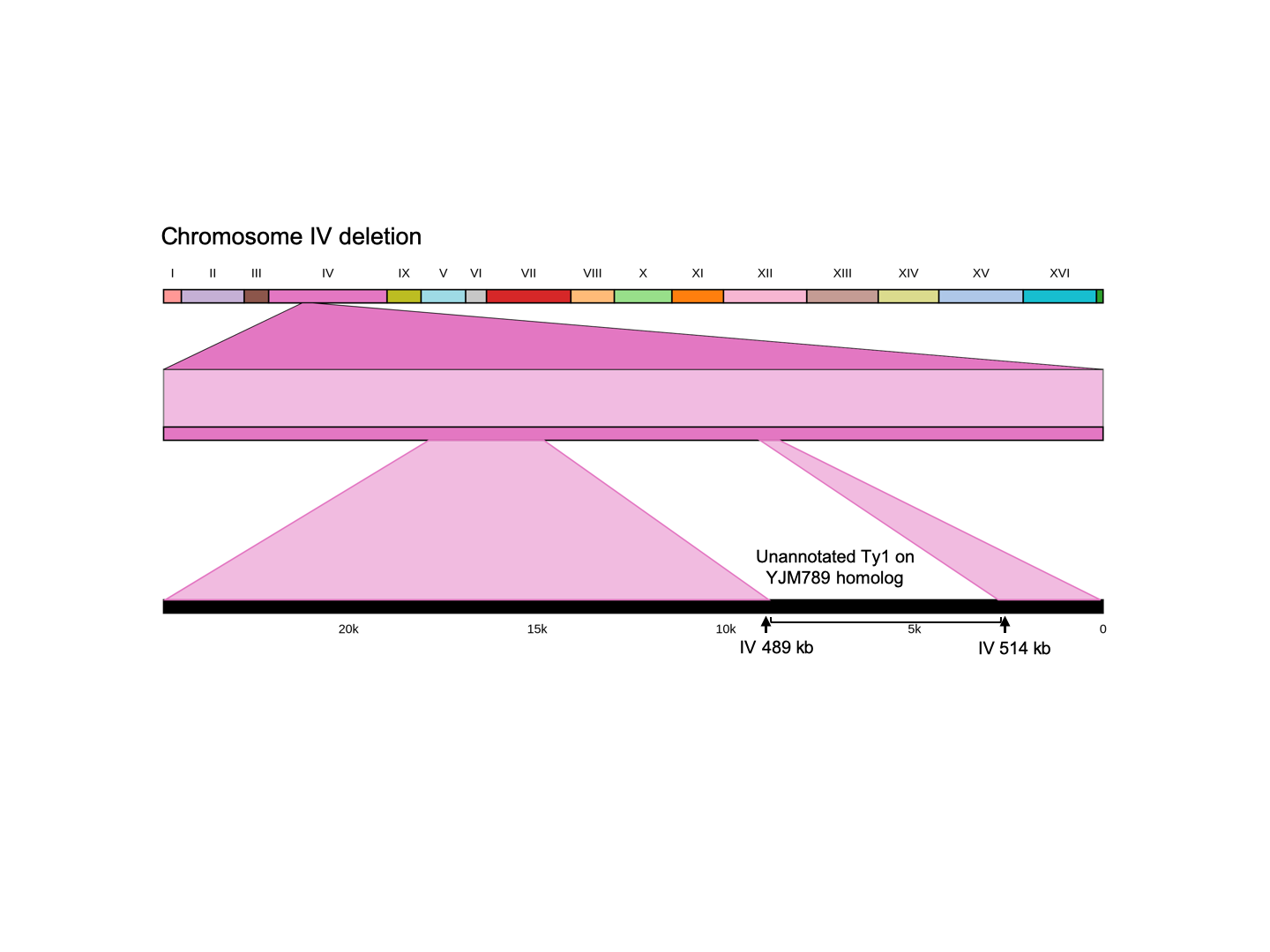

Supplement: S17 Fig — The I-DEL (coordinates 489–514 kb) on chromosome IV, evident by sequencing, has the same Ty-associated breakpoints as in MD741-5 (S9B Fig). The Ty elements that are at the breakpoints of the YJM789-derived homolog, but are absent in S288c are shown as a gap. (TIF) [file pgen.1010590.s017.tif]

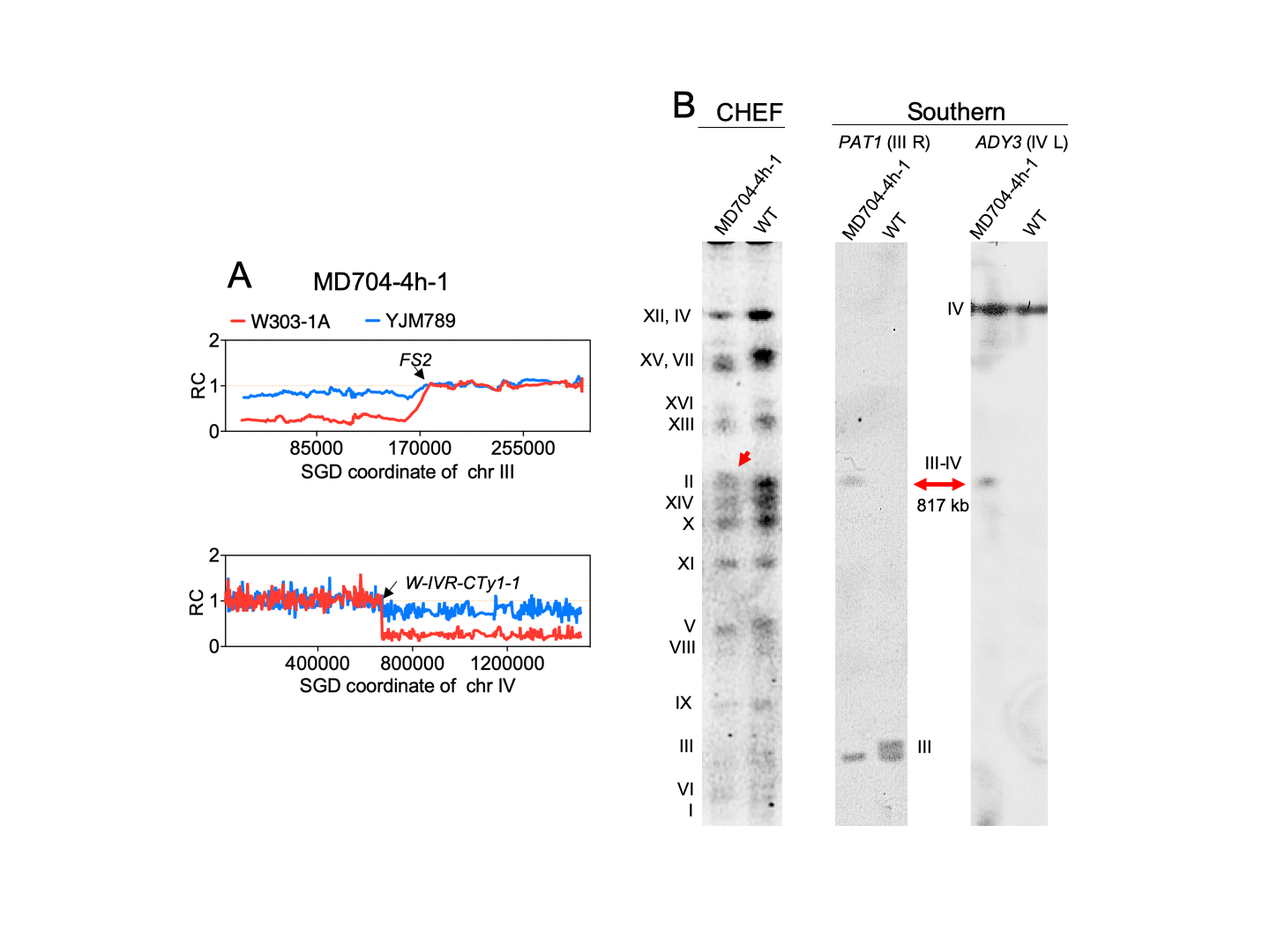

Supplement: S18 Fig — (A) By microarray analysis, this isolate has T-DEL events on chromosomes III and IV with breakpoints in Ty elements. As shown in S19 Fig, this pattern can be explained as a consequence of a crossover between the Ty elements on the right arms of chromosomes III (FS2 has an inverted pair of Ty elements near coordinates 169 kb) and IV (Ty element at coordinate 668 kb), followed by a non-disjunction event. (B) CHEF gel analysis demonstrating the III-IV translocation. By ethidium-bromide staining, there an incompletely visualized novel chromosome in MD704-4h-1 with a size of about 817 kb. This band hybridizes to a probe from the right arm of chromosome III (PAT1 located at coordinate 250 kb) and the left arm of chromosome IV (ADY3 at coordinate 26 kb). The expected size of this translocation is 817 kb. (TIF) [file pgen.1010590.s018.tif]

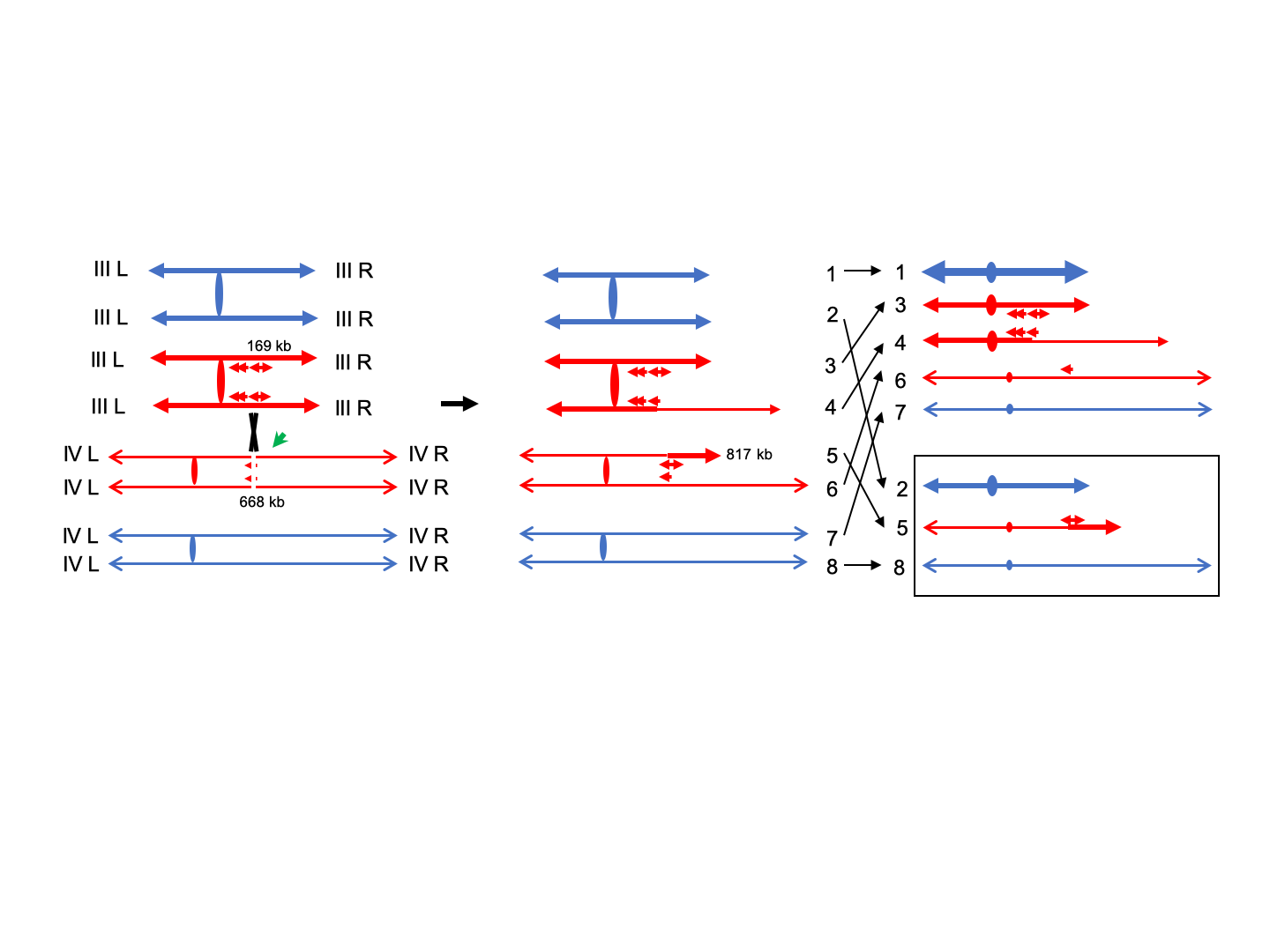

Supplement: S19 Fig — Chromosomes III and IV are shown as thick and thin lines, respectively, with the red color indicating W303-1A-derived homologs and the blue color indicating YJM789-derived homologs. The event begins with a crossover between a Watson-oriented Ty1 at coordinate 668 kb on IV and a Watson-oriented Ty1 on III in FS2 at 169 kb. The second step is a non-disjunction event in which both chromatids with CEN3 sequences derived from W303-1A are segregated into one daughter cell. The daughter cell that is outlined in the black rectangle will have the pattern of LOH events observed in MD704-4h-1. (TIF) [file pgen.1010590.s019.tif]

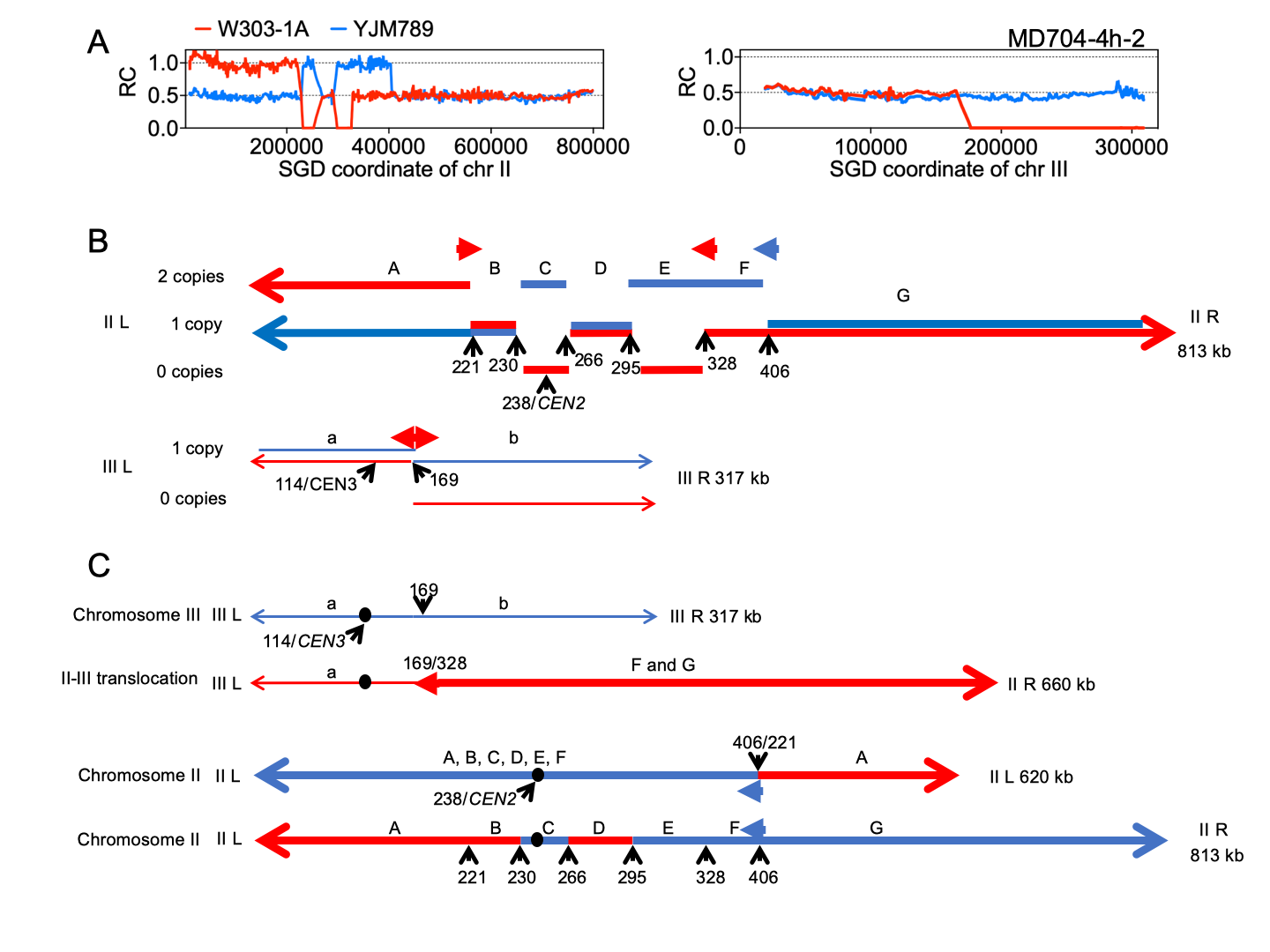

Supplement: S20 Fig — (A) Genomic sequencing analysis of gene dosages on chromosomes II and III. Chromosome II has multiple transitions between regions with different gene dosages, whereas chromosome III has a single T-DEL event. On chromosome II, S20A Fig does not show a small region of heterozygosity between coordinates 221–230 kb at the scale used in the figure. (B) Based on Dataset S2.3 in S2 Data, we depict the ratio of coverage for different segments of chromosome II and III with the breakpoints of transitions shown as SGD coordinates. Horizontal arrows indicate Ty elements. Red and blue lines show W303-1A and YJM789 sequences, respectively. Thick and thin lines represent chromosome II and III sequences, respectively. (C) Arrangement of chromosome segments in MD704-4h-2 that are consistent with the analysis in S20A and S20B. Recombination events that could produce these rearranged chromosomes are shown in S22 Fig. (TIF) [file pgen.1010590.s020.tif]

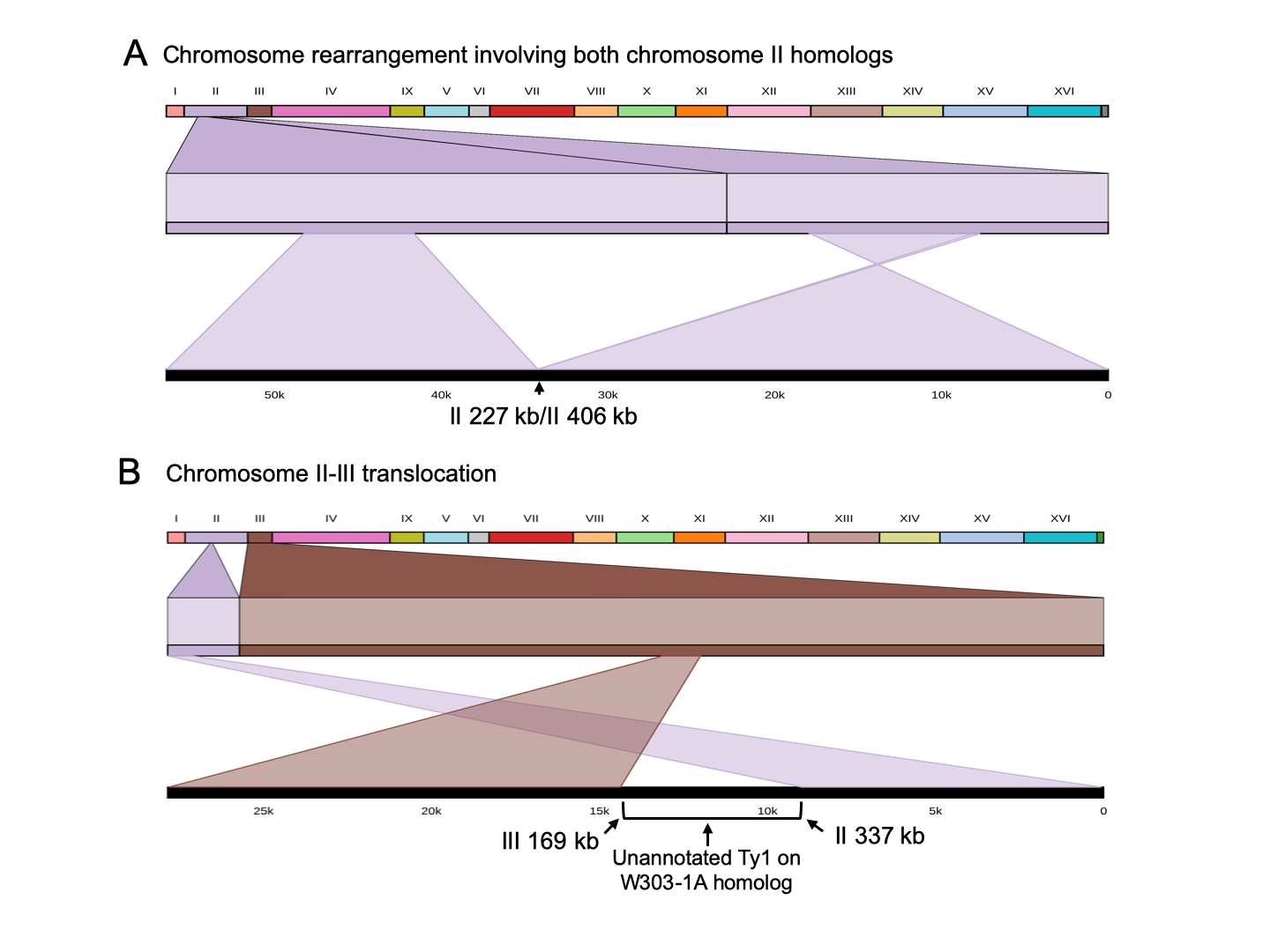

Supplement: S21 Fig — (A) Inversion/pseudo-isochromosome rearrangement on II. Based on microarray data, we predicted an inversion on chromosome II with a breakpoint fusing coordinates 227 kb and 406 kb (S22C Fig and S2 Data). By Nanopore sequencing, the inversion involves similar breakpoints at 226,953 and 405,572. There are Ty1 elements at both breakpoints. (B) II-III translocation. As predicted by the microarrays, the Nanopore sequencing confirmed a translocation between coordinate 336,548 of II and 168,934 of III; Ty1 sequences are located at both breakpoints. (TIF) [file pgen.1010590.s021.tif]

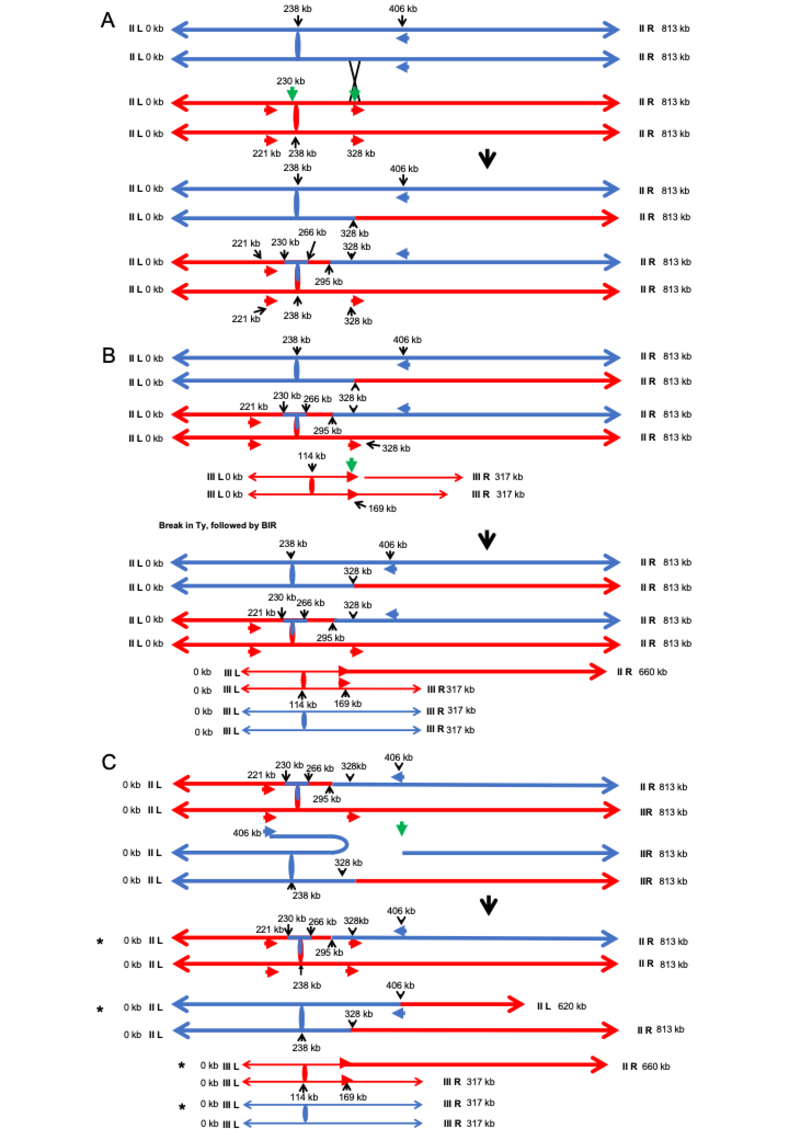

Supplement: S22 Fig — In this figure, we show one pathway that could produce the patterns of LOH and I-DELs observed in MD704-4h-2. Other homologous recombination events could likely produce the same patterns. Other observations in support of these chromosome rearrangements are described in S1 Text. W303-1A- and YJM789-derived homologs are shown as red and blue lines, respectively. Green arrows show the position of DSBs that initiate the events, and black arrows show the SGD coordinates at the breakpoints of the rearrangements (consistent with the depictions in S20 Fig). (A) Gene conversions and a crossover on chromosome II. One conversion event, unassociated with a crossover, is the result of a DSB near coordinate 230 kb on the W303-1A homolog with a conversion tract that extends through the centromere to coordinate 266 kb. A second DSB occurs in the Ty element near coordinate 328 kb. The repair of this DSB is associated with a conversion event that extends to coordinate 295 kb and a crossover between the two homologs. Ty elements are shown as short horizontal arrows. (B) Recombination between chromosomes II and III. A DSB in a Ty element located on the W303-1A homolog of chromosome III is repaired by a BIR event with a Ty1 element on one of the chromosome II homologs. The resulting translocation would be about 660 kb. Chromosome III and II are shown as a thin and thick lines, respectively. (C) Recombination between Ty elements located on opposite arms of chromosome II. A DSB within a Ty element located on the right arm of YJM789 homolog of II is repaired by a BIR event involving the Ty element located at 221 kb on the left arm of the W303-1A homolog of II. The resulting translocation would be 620 kb. Co-segregation of the chromatids marked with asterisks would produce the observed patterns of LOH. (TIF) [file pgen.1010590.s022.tif]

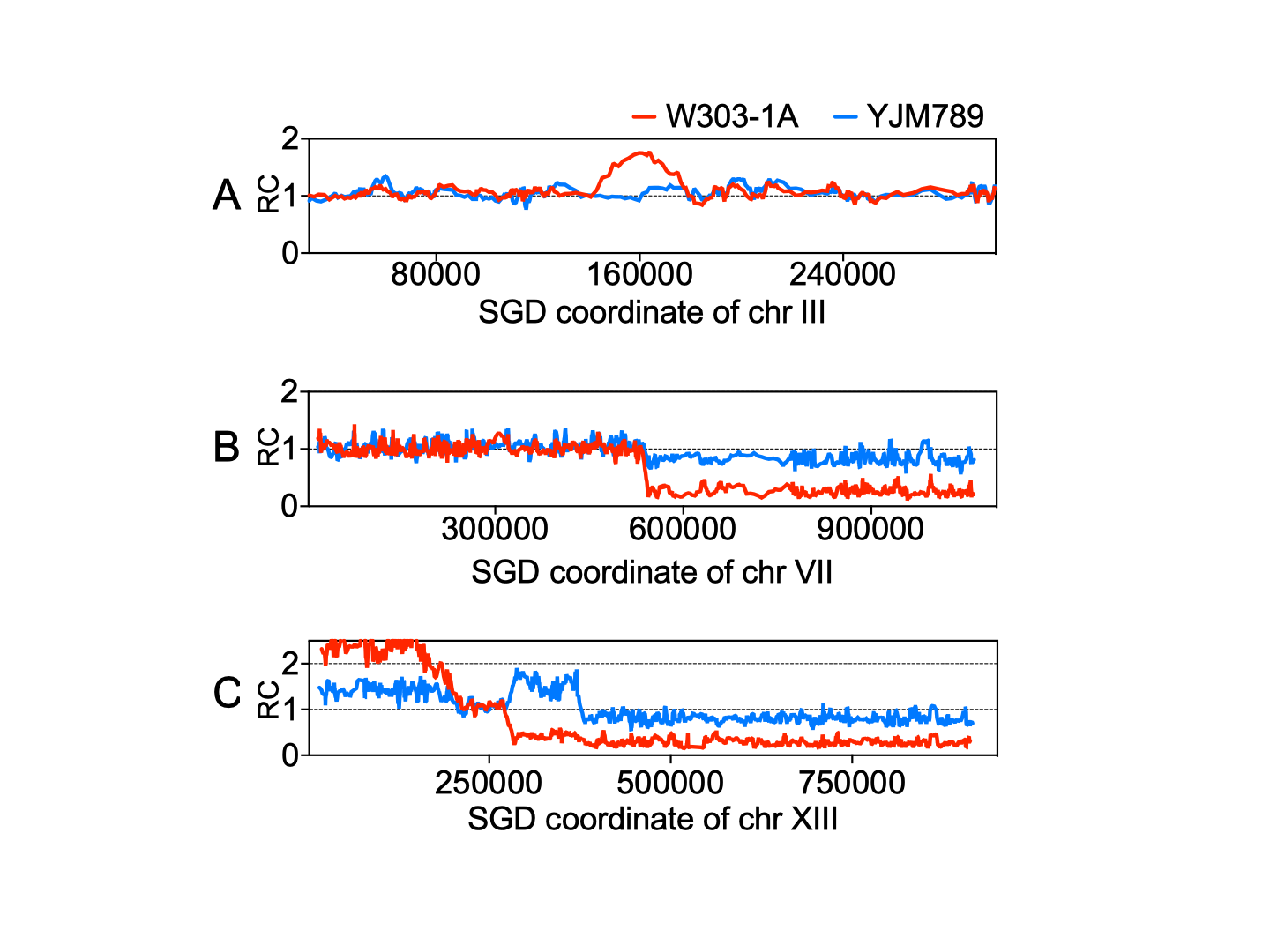

Supplement: S23 Fig — (A) Chromosome III has a duplication of the sequences between Ty elements located in FS1 and FS2 in the W303-1A homolog. (B) Chromosome VII has a T-DEL with a breakpoint in a Ty located near coordinate 536 kb. (C) Chromosome XIII has a complex pattern of alterations (breakpoints of each transition in parentheses): 1. Triplication of W303-1A-derived sequences (from left end of chromosome to 184 kb), 2. One copy of W303-1A and YJM789 sequences (184–280 kb), 3. Two copies of YJM789-derived sequences and no copies of W303-1A-derived sequences (280–372 kb), and 4. One copy of YJM789-derived sequences and no copies of W303-1A-derived sequences (372 kb to the right end of chromosome). (TIF) [file pgen.1010590.s023.tif]

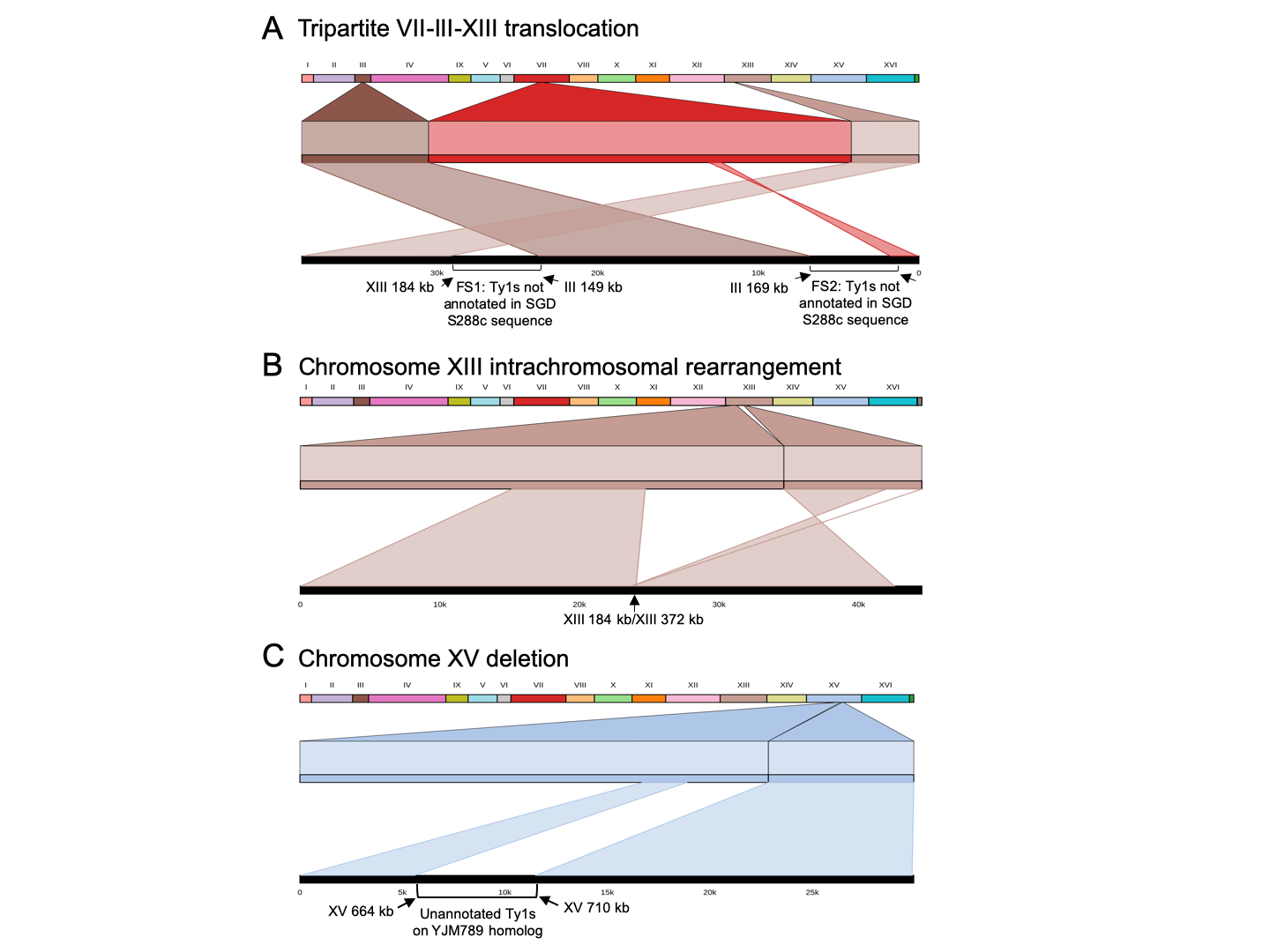

Supplement: S24 Fig — (A) Tripartite VII-III-XIII translocation. As shown in S23 and S25 Figs, the microarray experiments indicate formation of a tripartite translocation as the result of a DSB on chromosome VII being repaired by a BIR event involving chromosome III, followed by a dissociation and a re-invasion of chromosome XIII. All breakpoints occur at the positions of Ty elements. (B) Chromosome XIII-XIII pseudo-isochromosome. This event reflects a recombination event between chromosome XIII homologs involving a Crick-oriented Ty1 element on the right arm of XIII near coordinate 372 kb and a Watson-oriented Ty element on the left arm of XIII located near coordinate 184 kb (S23C Fig). (C) I-DEL on chromosome XV. The Nanopore sequencing confirms that the deletion on chromosome XV results from recombination between two Watson-oriented Ty1 elements located near coordinates 664 kb and 710 kb (S2 Data). (TIF) [file pgen.1010590.s024.tif]

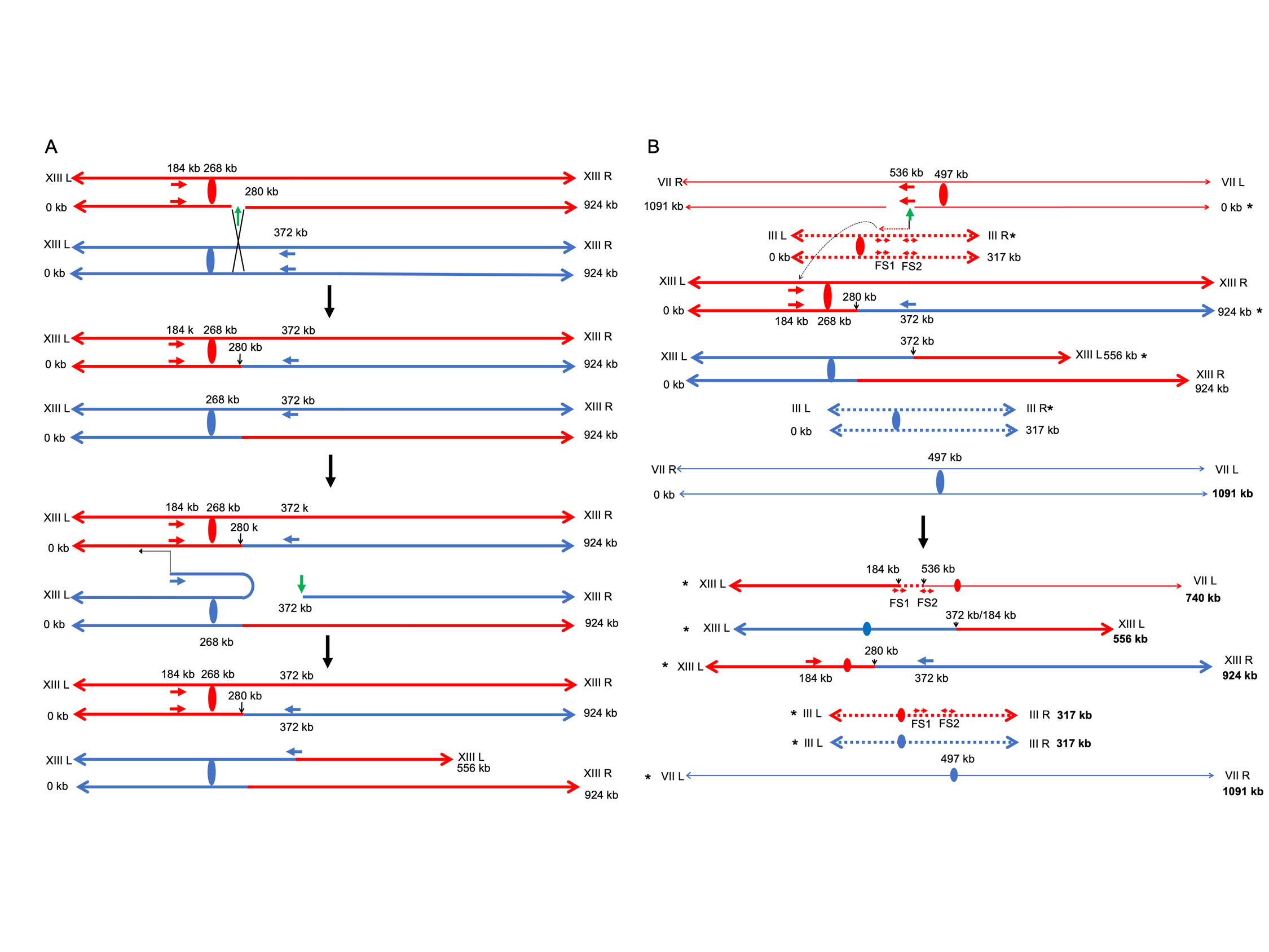

Supplement: S25 Fig — W303-1A and YJM789 homologs are drawn in red and blue, respectively; arrows show the location of Ty elements and ovals indicate centromeres. Chromosomes XIII and VII are shown as thick and thin lines, respectively. Chromosome III is shown as a dotted line. (A) Recombination events involving chromosome XIII. A reciprocal crossover occurs between the two homologs at a breakpoint near coordinate 280 kb. In a second event, a DSB within the YJM789 homolog at a Ty located at coordinate 372 kb is repaired by a BIR event involving an invasion of the end into a Ty element located at position 184 kb on the W303-1A homolog. The resulting chromosome is about 556 kb, and has sequences from the left arm of XIII on both ends. (B) Recombination between chromosomes VII and XIII. A break occurs on the W303-1A-derived VII at a Ty element located at 536 kb. The broken end is repaired by two consecutive BIR events, the first invading a Ty at 169 kb on chromosome III and replicating sequences to a non-allelic Ty at position 149 kb. The end is then extruded and invades a Ty on the left arm of chromosome XIII at coordinate 184 kb, performing a second BIR event. The resulting chromosome contains sequences derived from chromosomes III, VII, and XIII and is about 740 kb. The bottom part of this figure shows the chromosomes (marked with asterisks) that could segregate together to yield the observed microarray pattern. (TIF) [file pgen.1010590.s025.tif]

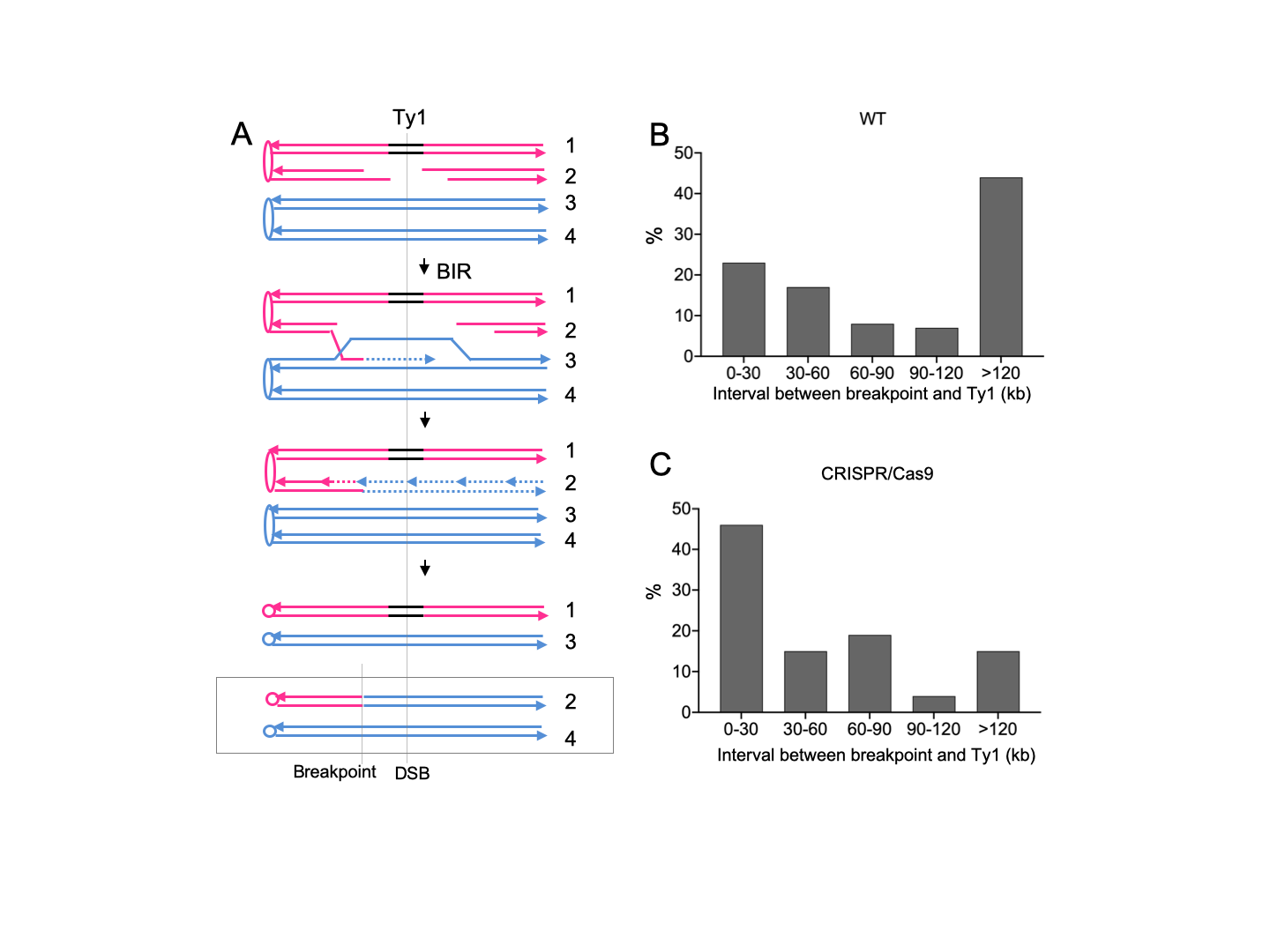

Supplement: S26 Fig — Chromosomes are shown as double-stranded DNA molecules with centromeres indicated as circles or ovals. Dotted lines indicate DNA synthesis during the BIR event. (A) A DSB in the Ty1 followed by degradation of the centromere-proximal broken end can produce a recombinant chromosome in which the breakpoint is displaced from the initiating DSB. (B) For spontaneous T-LOH events, we examined the distance between the recombination breakpoint and the nearest centromere-distal Ty1 (shown as a percentage of the total events) [12]. (C) For T-LOH events induced by CRISPR/Cas9, we show a bar graph comparable to that of S26B Fig. (TIF) [file pgen.1010590.s026.tif]
