# Supplementary material for: Shuffling the yeast genome using CRISPR/Cas9-generated DSBs that target the transposable Ty1 elements
Source: PLoS Genet. 2023 Jan 26;19(1):e1010590. doi: 10.1371/journal.pgen.1010590 (PMC9879454; doi:10.1371/journal.pgen.1010590)
Supplement: S1 Table — (DOCX) [file pgen.1010590.s031.docx]

**S1 Table. Yeast strains used in this study**

| **Strains** | **Background** | **Genotype** | **Construction** |
| --- | --- | --- | --- |
| LSY3877 | W303-1A | *MATa RAD5 leu2-3,112 trp1-1 ura3-1 can1 his3::GAL1p-CAS9-FLAG-HIS5(S. pombe) ade2-1* | [1] |
| QL62 | W303-1A | *MATa RAD5 leu2-3,112 trp1-1 ura3-1 can1 his3::GAL1p-CAS9-FLAG-HIS5(S. pombe) ade2-1* + pMD97 | Transform LSY3877 with pMD97 |
| FW588 | S288c | *MATα his4-912(URA3b) ura3-52* | [2] |
| MD743 | W303-1A x S288c | *MATa/MAT*α *RAD5/RAD5* *LEU2/leu2-3,112 TRP1/trp1-1 ura3-1/ura3-52 ADE2/ade2-1 HIS3/ his3::GAL1p-CAS9-FLAG-HIS5(S. pombe) HIS4/his4-912(URA3b) CAN1/can1* | Cross  LSY3877 with  FW588 |
| MD744 |  | *MATa leu2-3,112 trp1-1 ade2-1 his3::GAL1p-CAS9-FLAG-HIS5(S. pombe) his4-912(URA3b) CAN1* | Spore colony  from MD743 |
| MD745 |  | *MATa leu2-3,112 trp1-1 ade2-1 his3::GAL1p-CAS9-FLAG-HIS5(S. pombe) his4-912(URA3b) CAN1* +pMD97 | Transform  pMD97 into  MD744 |
| MD747 |  | *MATa leu2-3,112 trp1-1 ade2-1 his3::GAL1p-CAS9-FLAG-HIS5(S. pombe) his4-912(URA3b) CAN1* +pAA2 | Transform pAA2 into MD744 |
| JSC20-1 | YJM789 | *MAT*α *ura3 gal2 ade2-1 ho::hisG IV1510886::SUP4-o* | [3] |
| JSC21-1 | YJM789 | *MAT*α *ura3 can1::natMX4 gal2 ade2-1 ho::hisG IV1510886::SUP4-o* | [3] |
| JSC24-2 | W303-1A x YJM789 | *MATa/MATα::natMX4 ura3/ura3-1 ade2- 1/ade2-1 trp1-1/TRP1 his3-11,15/HIS3 leu2-3,112/LEU2 RAD5/RAD5 IV1510386:kanMX6-can1- 100/IVI1510386::SUP4-o GAL2/gal2* | [3] |
| MD702 | YJM789 | *MATα leu2::hphMX4 ura3 can1::natMX4 gal2 ade2-1 ho::hisG IV1510886::SUP4-o* | Replace *LEU2*  with *hphMX4*  in JSC21-1 |
| MD703-3 | W303-1A x YJM789 | *MATa/MATα RAD5/RAD5 leu2-3,112/leu2::hphMX4 trp1-1/TRP1 ura3-1/ura3 can1/can1::natMX4 HIS3/ his3::GAL1p-CAS9-FLAG-HIS5(S. pombe) ade2-1/ade2-1 GAL2/gal2 IV1510886/IV1510886::SUP4-o* | Cross LSY3877 with MD702 |
| MD704-A | W303-1A x YJM789 | *MATa/MATα RAD5/RAD5 leu2-3,112/leu2::hphMX4 trp1-1/TRP1 ura3-1/ura3 can1/can1::natMX4 HIS3/ his3::GAL1p-CAS9-FLAG-HIS5(S. pombe) ade2-1/ade2-1 GAL2/gal2 IV1510886/IV1510886::SUP4-o* + pMD97 | Transform pMD97 into MD703 |
| MD704-B | W303-1A x YJM789 | *MATa/MATα RAD5/RAD5 leu2-3,112/leu2::hphMX4 trp1-1/TRP1 ura3-1/ura3 can1/can1::natMX4 HIS3/ his3::GAL1p-CAS9-FLAG-HIS5(S. pombe) ade2-1/ade2-1 GAL2/gal2 IV1510886/IV1510886::SUP4-o* + pMD97 | Transform pMD97 into MD703 |
| MD741 | W303-1A x YJM789 | *MATa/MATα RAD5/RAD5 leu2-3,112/leu2::hphMX4 trp1-1/TRP1 ura3-1/ura3 can1/can1::natMX4 HIS3/ his3::GAL1p-CAS9-FLAG-HIS5(S. pombe) ade2-1/ade2-1 GAL2/gal2 IV1510886/IV1510886::SUP4-o* | Isolate of MD704 grown to form colony on 2% galactose medium, followed by loss of pMD97 |

**References**

1. Al-Zain AM. Mutagenic repair outcomes of DNA double-strand breaks. : Columbia University; 2021.

2. Roeder GS, Fink GR. Construction of yeast strains containing genetically marked transposons. Fed Proc. 1982;41(10):2653-2655. <https://doi.org/10.1371/journal.pgen.1008632> PMID: 6286365

3. St Charles J, Petes TD. High-resolution mapping of spontaneous mitotic recombination hotspots on the 1.1 Mb arm of yeast chromosome IV. PLoS Genet. 2013;9(4):e1003434. <https://doi.org/10.1371/journal.pgen.1003434> PMID: 23593029
