# Supplementary material for: Shuffling the yeast genome using CRISPR/Cas9-generated DSBs that target the transposable Ty1 elements
Source: PLoS Genet. 2023 Jan 26;19(1):e1010590. doi: 10.1371/journal.pgen.1010590 (PMC9879454; doi:10.1371/journal.pgen.1010590)
Supplement: S2 Table — (DOCX) [file pgen.1010590.s032.docx]

**S2 Table. Primers used in this study.**

| **Primer** | **Sequence (5' to 3')** | **Purpose** |
| --- | --- | --- |
| LEU2 KO F | CAAGGATATACCATTCTAATGTCTGCCCCTAAGAAGATCGTCGTTTTGCCCGTACGCTGCAGGTCGAC | Knock out *LEU2* with *hphMX4* |
| LEU2 KO R | CGGCGACAGCATCACCGACTTCGGTGGTACTGTTGGAACCACCTAAATCATCGATGAATTCGAGCTCG |  |
| LEU2 KO CHK F | CCAATAGGTGGTTAGCAATCGTC | Verify *LEU2* knock out |
| HygCK R | CAGAAACTTCTCGACAGACGTC |  |
| Ty1.A.for.2 | TTAACGAATCAGATGTCGACgtttt | pMD97 construction primers |
| Ty1.A.rev.2 | GTCGACATCTGATTCGTTAAgatca |  |
| SNR52F | CTTGGGCTAGCGGTAAAGGTGCGC | Verify pMD97 construction |
| ADH1R | CAGGTATAGCATGAGGTCGCTC |  |
| CHKdeltaF | TATTACACAGCGCAGTTGTG | Classify 5-FOA-resistant colonies as Class 1, 2, 3, or 4. |
| CHKdeltaR-3 | GCAGTTCTTCTAGGCAGTCG |  |
| URA3 R | TCCTTCTGCTCGGAGATTAC |  |
| Ty IN | TCACTATGCATTAATGACATC |  |
| Ty OUT | TGAGAGTTAGCCTTAGTGGA |  |
| Ty912-URA3-S | GAAGAAACATGAAATTGCC | Check *URA3* mutation in Ty912 |
| Ty912-URA3-A | AATTGAAGCTCTAATTTGTGAG |  |
| FLC2 F | CACAAACGACACTACTCCGGC | Generate probe to Chromosome I |
| FLC2 R | GTATTGATGATACCCATGGAC |  |
| SEA4 F | CCAGCGGTGGTAAGGCAGCTC | Generate probe to left arm of II |
| SEA4 R | CTTGACGCTGCACCGGATCCA |  |
| HPR1 F | CTCCATTCGACATGAACCACTTC | Generate probe to right arm of IV |
| HPR1 R | CACGTGCTGATCTGTTCCATTC |  |
| PDA1 F | CTTCGCACTCCCACCAGGATAG | Generate probe to right arm of V |
| PDA1 R | CGCGGAGGATCTTGAAGCGGC |  |
| SOR1 F | CTGGTATCTGCGGCTCTGATATTC | Generate probe to Chromosome X |
| SOR1 R | CGGCATCAATGCAAACATCAG |  |
| RIF2 F | CAGTGACAAGATTGTGAAAGC | Generate probe to right arm of XII |
| RIF2 R | GTCGATAGGAGAGACGAAATGC |  |
| PGA3 F | CGGGATCTATATTCCTGCCGC | Generate probe to left arm of XIII |
| PGA3 R | GTCTCATTGGCATATAGCAGGGAG |  |
| FRE5 F | CAATGGGACCAGATAGCAATTG | Generate probe to right arm of XV |
| FRE5 R | CACTTGGCTTCTGCGATCACTGAG |  |
| PLC1 F | CGCGAACAATGGAAGTACTGTG | Generate probe to left arm of XVI |
| PLC1 R | GACCAGTTGACACATAAATATG |  |
| II-304kS | TGACATTGTTTCTTTCCACG | Generate probe to II, *HMT1* |
| II-304kA | CTAAGAGGCAAGTTGGAGG |  |
| III-128kS | GTTCCATCGGCGTCTAAAT | Generate probe to III, *SAT4* |
| III-128kA | GCTGATGTGGGTGATAAGGA |  |
| IV-1095 S | AACCCCTTAGGTAGTTCTCC | Verify III-IV translocation in MD741-9 |
| III-170 A | GCAAGATGTTCTGAAGGATG |  |
| IV-1090221S | TTCTATTCTTCTCTTCGGTGA | Generate probe to IV, *RAD34* |
| IV-1090547A | ACTACTGGGACTCTCGTTCT |  |
| III-170778S | CTTTAGAAGTGTCTTAGCGG | Generate probe to III, *FEN2* |
| III-171122A | CCCAAGTTTGAGAGAGGTT |  |
| III-165056S | AATAACATTTGAGGGAACTACG | Generate probes to III, *NPP1* |
| III-165304A | ACACTGAACGAAAGACCACA |  |
| IV-1102832S | ACTATGGTGTGCTCTTTGGG | Generate probes to IV, *HIM1* |
| IV-1103123A | ATTAGGATTTCCGTGCTGTC |  |
| IV26kS | CAACTGTAGGAATAGGCTCG | Generate probe to IV, *ADY3* |
| IV26kA | CTCTTCATTTTGGGACTGG |  |
| VII-459kS | GGTGAAGAAGAGACGAAAGT | Generate probe to VII, *ATE1* |
| VII-459kA | AGAAATAGTCTTCCCTCCAG |  |
| XV-S | ATGCAGTCAACATCTCCCTT | Verify the circular DNA formation in MD741-18 |
| XV-A | CTTCACAGTGCTTCCTATGG |  |
| Ty1-1 | GTACCCACAGCAGTGCATGATG | Southern blot probes for Ty1 element |
| Ty1-2 | GATATGAATGCCATTATTGTTCAG |  |
| Ty1-3 | CATACTGACATATTTGGTCCAG |  |
| Ty1-4 | GATAGATGATATATCCATAAGAG |  |
| Ty1-5 | CGTTGATGATATGATATTGTTC |  |
| Ty1-6 | GTAGTTGAAGTACATGTTAATG |  |
