# Supplementary material for: Shuffling the yeast genome using CRISPR/Cas9-generated DSBs that target the transposable Ty1 elements
Source: PLoS Genet. 2023 Jan 26;19(1):e1010590. doi: 10.1371/journal.pgen.1010590 (PMC9879454; doi:10.1371/journal.pgen.1010590)
Supplement: S3 Table — (DOCX) [file pgen.1010590.s033.docx]

**S3 Table. PCR diagnosis of MD745 5-FOA^R^ mutants.**

| 5-FOA^R^ Classes | Number of events | PCR product size (bp)^1^ | | | |
| --- | --- | --- | --- | --- | --- |
|  |  | 1 | 2 | 3 | 4 |
| WT |  | 8038 | 3586 | 638 | 1460 |
| Class 1 | 1 | 8038 | 3586 | 638 | 1460 |
| Class 2 | 25 | 646 | No band | No band | No band |
| Class 3 | 9 | 6876 | 2424 | 638 | No band |
| Class 4 | 1 | Non-specific | 2424 | 638 | No band |

The primers for the PCR reactions labeled 1-4 were: 1 (CHKdeltaF and CHKdeltaR-3) 2 (CHKdeltaF and Ty IN), 3 (Ty OUT and CHKdeltaR-3), and 4 (CHKdeltaF and URA3 R).
