# Supplementary material for: Shuffling the yeast genome using CRISPR/Cas9-generated DSBs that target the transposable Ty1 elements
Source: PLoS Genet. 2023 Jan 26;19(1):e1010590. doi: 10.1371/journal.pgen.1010590 (PMC9879454; doi:10.1371/journal.pgen.1010590)
Supplement: S1 Text — (DOCX) [file pgen.1010590.s034.docx]

**S1 Text**

**Shuffling the yeast genome using CRISPR/Cas9-generated DSBs that target the transposable Ty1 elements**

Lei Qi, Yang Sui, Xing-Xing Tang, Ryan J. McGinty, Xiao-Zhuan Liang, Margaret Dominska, Ke Zhang, Sergei M. Mirkin, Dao-Qiong Zheng, Thomas D. Petes

**Mapping of Ty elements in the W303-1A and YJM789 genomes relative to Ty elements in Saccharomyces Genome Database (SGD) reference strain S288c**

In the genome of our isolate of the haploid S288c, there are 58 complete Ty elements (SGD annotation), the most common elements being the related elements Ty1 (39 copies) and Ty2 (13 copies) (Dataset S1.1 in S1 Data). The guide RNA used in our experiments is specific for all 39 copies of Ty1 (Fig 3A). In a BLAST search of the S288c genome, we identified two partial Ty1 elements (one located on chromosome XII (near SGD coordinate 215 kb) and on chromosome IV (near SGD coordinate 803 kb) that also match the guide RNA target sequence. In addition to the delta elements associated with complete Ty1 elements, there are 279 solo delta elements [1]. Although the delta element does not contain a match to the guide RNA, it is possible that a DSB in a Ty1 element, following processing of the broken end, could be repaired by recombination with a delta element [2].

Using the sequencing data derived from Pacific Biosciences (PacBio) technology, Matheson *et al.* [3] examined the genome of the haploid strain W303-1A. In this assembled genome of W303-1A, we detected 58 intact Ty elements including 37 Ty1, 15 Ty2, 3 Ty3, 2 Ty4, and 1 Ty5 (Dataset S1.2 in S1 Data). This strain also has the same two incomplete Ty1 elements with guide RNA targets described above for S288c. Lastly, we sequenced the genome of a YJM789-derived strain JSC20-1 [4] by PacBio technology. 17 Ty1 and 9 Ty2 elements were identified in the YJM789 genome (Dataset S1.3 in S1 Data).

The distributions of Ty1 elements in S288c, W303-1A, and YJM789-derived are compared in S2 Fig. Out of the 58 Ty elements in W303-1A, 44 share the same locations with those of S288c genome. In addition, these two strains are highly conserved in DNA sequence (<0.1% sequence divergence) [3]. The YJM789 genome has about half of the number of Ty1 elements as the other two genomes, and the location of these elements are not well conserved with the other two genomes. For example, there are only three Ty1 elements that share the same location and orientation between W303-1A and YJM789 genomes (S2 Fig). Consistent with our previous Southern analysis [5], chromosome III in W303-1A had two tandem Ty1 elements located near SGD coordinate 149 kb and two Ty1 elements arranged as inverted repeats near coordinate 169 kb (S2 Fig). These two pairs of Ty1 elements (termed fragile sites 1 (FS1) and 2 (FS2)) are hotspots for mitotic recombination in strains with low levels of DNA polymerases alpha [5] and delta [6]. We also sequenced our isolate of S288c using the Nanopore technology and found that both FS1 and FS2 are present in chromosome III of this isolate (S2 Fig). Thus, even different isolates of purportedly the same strain can have different patterns of Ty insertions.

**Strain construction**

The genotype of strains used in this study are listed in S1 Table, and all primers used for strain construction or analysis are listed in S2 Table. The haploid strain used in most of our constructions (LSY3877) was provided by L. Symington (Columbia University) and is described in Al-Zain [7]. LSY3877 was derived from W303-1A [8]by integrating the *Asc*I-treated plasmid pRG203Cas9 into the yeast strain LSY0678 [7]. The resulting genotype is: *MAT***a** *RAD5 leu2-3,112 trp1-1 ura3-1 can1 his3::GAL1p-CAS9-FLAG-HIS5(S. pombe) ade2-1*. In this strain, the expression of the *CAS9* gene is regulated by the galactose-inducible *GAL1,10* promoter. It should be noted that the *S. pombe HIS5* gene complements the *S. cerevisiae his3* mutation. The haploid MD702 was constructed by transforming the YJM789-derived strain JSC21-1 [4] with a PCR fragment containing the *hphMX4* gene obtained by amplifying the plasmid pAG32 [9] with the primers LEU2 KO F and LEU2 KO R. The genotype of MD702 was *MAT*α *leu2::hphMX4 ura3 can1::natMX4 gal2 ade2-1 ho::hisG IV1510886::SUP4-o*. The diploid strain MD703-3 was constructed by mating LSY3877 and MD702. In addition to the markers shown in S1 Table, MD703-3 is heterozygous for about 55,000 SNPs that allow the mapping of multiple classes of chromosome alterations [4].

We transformed MD703-3 with the plasmid pMD97 which contains a guide RNA specific for Ty1 elements, and has *LEU2* as a selectable marker for transformation. The construction of pMD97 is described below. The strains transformed with pMD97 are MD704-A and MD704-B. MD704-B derivatives, before galactose-induction of *CAS9*, had two small LOH regions not present in the parental strain MD703-3. There was an interstitial LOH event on chromosome VII between coordinates 759-783 kb that duplicated YJM789 sequences, and an I-LOH event on chromosome IX (coordinates between 198 and 224 kb) that duplicated W303-1A-derived sequences. Neither of the LOH intervals contained Ty1 elements. The *CAS9*-induced chromosome rearrangements in MD704-A and MD704-B strains were similar, and data from these two strains were pooled. Following colony formation on galactose-containing medium, we purified derivatives of independent colonies that had lost the pMD97 plasmid. These isolates were designated MD741, followed by numbers indicating independent isolates.

We constructed a haploid strain (MD744) in order to examine the effects of expressing a Ty1-targeted *CAS9* activity on the expression of a single marked Ty1 element. The initial step in the construction was a cross of the S288c derivative FW588 (*MAT*α *his4-912(URA3b) ura3-52*) to LSY3877. FW588 [10] contains a Ty1 element with an insertion of *URA3* within the element (*his4-912(URA3b)*). Loss of the *URA3* insertion (selectable on medium containing 5-fluoro-orotate) can occur by a variety of recombination mechanisms including gene conversion with an unmarked Ty element at a non-allelic location, “pop-out” recombination between the two long terminal repeats that flank the element, and others. Following the cross of FW588 to LSY3877, we sporulated the resulting diploid (MD743), and dissected tetrads. We selected a spore (MD744) with the genotype: *MAT***a** *leu2-3,112 trp1-1 ade2-1 his3::GAL1p-CAS9-FLAG-HIS5(S. pombe) his4-912(URA3b) CAN1*. MD744 was transformed with pMD97 to detect the effects of expressing the Ty1-targeted *CAS9* protein on the *his4-912(URA3b)* marked Ty (MD745). The control strain MD747 was a transformant of MD744 with pAA2, a plasmid without the guide RNA.

**Plasmids used in the study**

The plasmid pMD97 was constructed by inserting an oligonucleotide with the sequence of the Ty1-specific sgRNA into a unique *Bae*I site downstream of the *SNR52* promoter in the plasmid pAA2 using primers Ty1.A.for.2 and Ty1.A.rev.2 (S2 Table). The plasmid pMD97 in this study was based on pAA2 which was derived from the plasmid pML107 [11]. The plasmid pML107 is a 12.4-kb plasmid containing *CAS9*, *LEU2*, a replication origin derived from the two-micron yeast plasmid, and a polycloning site located downstream of the *SNR52* promoter to allow insertion of sequences of a guide RNA. The plasmid pAA2 [7] was derived from the plasmid pML107 in several steps: 1. Deletion of an *Xho*I fragment containing the *CAS9* gene, 2. Deletion of a *Bae*I site located in the *LEU2* gene, and 3. Insertion of an oligonucleotide containing a *Bae*I site downstream of the *SNR52* promoter. To construct pMD97, we inserted an oligonucleotide containing the guide RNA sequences (produced by annealing primers Ty1.A.for.2 and Ty1.A.rev.2; S2 Table) into the *Bae*I-treated pAA2 plasmid. The sequence of the insertion in the resulting plasmid (pMD97) was confirmed by PCR and Sanger sequencing using the primers SNR52F and ADH1R (S2 Table).

**Analysis of translocations and other chromosome rearrangements by microarrays, CHEF gel electrophoresis and Nanopore sequencing**

*MD741-3*

By DNA sequencing, this isolate had two T-DELs, one on II (328 kb, *W-IIR-CTy1-2*) and one on XIII (372-379 kb, *YMRCTy1-4*) (S7 Fig). In addition, the isolate had two T-DUPs, one on XII (592-601 kb, *Y-XIIRCTy1-1*) and one on XIII (196-202 kb, *YMLWTy1-2*). There are Ty elements located at the breakpoints of both deletions and duplications. From our previous studies, most of the isolates with both T-DELs and T-DUPs reflect translocations with the joining of a centromere-containing fragment to an acentric fragment. The predicted sizes of the acentric fragments (duplicated regions) are 479 kb (XII) and 196 kb (XIII); the predicted sizes of the centromere-containing fragments are 328 kb (II) and 372 kb (XIII). To get two translocations with single centromeres, we either have 328+479= 807 kb and 372+196=568 kb chromosomes or 328+196= 524 kb and 372+479=851 kb chromosomes. The observed novel chromosomes have sizes of about 530 kb and 840 kb (S7 Fig). Therefore, the sizes of the translocations suggest that the two translocations are: II-XIII (530 kb) and XIII-XII (840 kb).

The II-XIII translocation hybridizes to a probe from the left end of II (*SEA4*; 18-21 kb) (S7 Fig). This probe also hybridizes to several other chromosomes, particularly chromosome I. This translocation also hybridizes to a probe from the left end of XIII (*PGA3*, 20-22 kb). The XIII-XII translocation hybridizes to the *PGA3* probe, and to the *RIF2* probe from the right end of XII (1041-1043 kb). The sequencing data suggest that the XIII-XII translocation is likely not present in all cells of the MD741-3 isolate. It should also be noted that a T-LOH event on XII near coordinate 480 kb partially obscures the duplication on XII.

*MD741-5*

Based on microarrays, there are three T-DUPs and three T-DELs, suggesting the formation of three translocations. The breakpoints of all of the duplications/deletions are at Ty elements (S8 Fig). The predicted sizes of the acentric fragments (duplicated regions) are: 220 kb (II), 250 kb (X), and 184 kb (XIII) (S2 Data). The predicted sizes of the centromere-containing fragments are: 160 kb (I), 1100 kb (IV), and 970 kb (XV). The sizes of the new bands on the CHEF gel are about 380 kb, 1157 kb, and 1360 kb (S8 Fig). Assuming that these translocations require one duplication and one deletion, the only pair small enough to represent the 380 kb translocation are I-II. The other possible pairs are: X-IV (1360 kb); XV-XIII (1157 kb) or XIII-IV (1280 kb) and XV-X (1220 kb). The X-IV and XV-XIII pairs fit the data best. The I-II pair was confirmed by showing that the 380 kb band hybridized to a probe from the left end of I (*FLC2* at 46 kb) and the left end of II (*SEA4* at 18-21 kb) (S8 Fig). The XIII-XV translocation was confirmed using a probe from the left end of XIII (*PGA3* at 20-22 kb) and the right arm of XV (*FRE5* at 1062 kb). The X-IV translocation was confirmed using a probe from the right end of X (SOR1, 736 kb) and the right end of IV (*HPRI*, 730 kb). For all three translocations, the Ty elements were in the correct orientation to produce monocentric chromosomes. There is also an internal deletion of 36 kb on chromosome IV with Ty elements at the breakpoints. There is a chromosome in the CHEF gel that is smaller than the “normal” IV, consistent with this deletion.

The microarray data and the Nanopore sequencing for MD741-5 were not in complete agreement. Nanopore sequencing confirmed the I-II translocation, and the I-DEL of chromosome IV predicted by the microarray data (S9 Fig). However, there were two additional translocations (XIII-XV and IV-X) that were detectable by microarray analysis that were not observed by sequencing. One possible reason for this discrepancy was that the MD741-5 isolate contained cells with a mixture of different chromosome rearrangements. We purified two single colonies from the MD741-5 isolate, and examined patterns of LOH, deletions, and duplications by microarrays. Some patterns were conserved between the original isolate, and the two sub-clones. For example, the I-DEL on chromosome IV had the same breakpoints in all three strains (S10A Fig). However, other events were different. In the original isolate and one of the sub-clones, there was a T-DEL on chromosome IV with a breakpoint at about 1.1 Mb. In the other sub-clone, instead of a T-DEL at that position, there was a T-LOH event (duplication of YJM789-derived sequences and loss of W303-1A-derived sequences) (S10A Fig); in addition, the T-DUP on chromosome X that was observed in the original isolate and one of the sub-clones was missing in the other sub-clones (S10B Fig). This observation suggests that a DSB in the Ty element near 1.1 Mb on chromosome IV was repaired in two different pathways within a single isolate, one pathway generating a IV-X translocation and a second pathway producing an LOH event on chromosome IV. Thus, at least some of the isolates of MD741 had on-going genetic instability even in the absence of the guide RNA. Causes of on-going genetic instability could be: 1. Continued expression of CRISPR/Cas9 for several cell cycles until the guide RNA and CAS9 protein are sufficiently diluted, 2. Formation of dicentric chromosomes that give rise to continued chromosome breakage, and 3. Delayed repair of DSBs, allowing different patterns of rearrangements in different cells of the isolate. It should be emphasized, however, that in most of the samples examined by both microarray analysis and Nanopore sequencing, the same alterations were observed.

*MD741-6*

Although most paired terminal duplications and deletions reflect the formation of translocations, one exception was observed in the isolate MD741-6. From DNA sequence analysis (S11 Fig), this strain has two T-DELs and two T-DUPs. The terminal deletions are on XIII with a breakpoint in a Ty1 Crick located at position 372 kb, and on chromosome XVI with a breakpoint in a Ty1 Crick located at position 803 kb. The T-DUPs are on XIII with a breakpoint in a Ty1 Watson with a breakpoint at about 196 kb and on XVI in a Ty Watson with a breakpoint about 56 kb. The predicted sizes of XIII-XVI translocations are: 432 kb and 999 kb. Alternatively, if the deletion-duplications occur within a single chromosome, the predicted product for XIII is 568 kb and for XVI is 866 kb. There is a novel band at about 860 kb on the CHEF gel (S11 Fig) close to the size predicted for the intrachromosomal chromosome XVI rearrangement. There is also a band near the position of chromosome V, about 580 kb. The band at 860 kb hybridizes with *PLC1* (left arm of XIV at 36 kb). The band at 580 kb hybridizes to *PGA3* (left arm of XIII at 20-22 kb). In summary, the two chromosome rearrangements in MD741-6 both represent intrachromosomal deletion/duplication events associated with recombination between Ty1 elements. The orientations of the elements are expected to generate monocentric chromosomes. The internal chromosome rearrangements on XIII and XVI were also confirmed by Nanopore sequencing (S12 Fig).

*MD741-7*

The chromosome rearrangements in this isolate are depicted in Fig 6 and are described in the main text. The isolate has a V-XIII translocation and a 96 kb internal deletion on chromosome XII on the YJM789-derived homolog. The translocation was confirmed using probes derived from chromosomes V and XIII to the translocation. There are two Crick-oriented Ty elements at the breakpoints on XII that would be expected to generate the deletion by homologous recombination. The V-XIII translocation and the deletion on XII were confirmed by Nanopore sequencing (S13 Fig).

*MD741-8*

This isolate has one T-DEL (II near coordinate 328 kb) and one T-DUP (XIII near coordinate 184 kb) (S14A Fig). Ty elements are present at both breakpoints (S2 Data). The expected size of the acentric fragment is 190 kb, and the expected size of the centromere-containing fragment is 328 kb. Thus, a translocation between II and III would be expected to be about 510 kb, and a chromosome of this size is detected by CHEF gel analysis (S14C Fig). This novel chromosome hybridizes to probes from chromosomes II and XIII.

*MD741-9*

In this isolate (S14B Fig), we observed a T-DEL of W303-1A sequences on chromosome II with a breakpoint near a Crick-oriented Ty1 element on the right arm of chromosome II (coordinate 643 kb). In addition, there was a T-DUP of W303-1A sequences on the left arm of chromosome XVI with a breakpoint near a Watson-oriented Ty element (coordinate 56 kb). The expected size of a translocation involving these elements is 643 kb (centric fragment) plus 56 kb (acentric fragment) or 699 kb. A band of about 700 kb hybridizes to probes from chromosomes II and XVI as expected (S14C and S14D Figs).

In addition to the translocation described above, the MD741-9 isolate had two novel bands on the CHEF gel, one of approximately 1240 kb and one of approximately 610 kb (S15 Fig). In addition, the W303-1A chromosome III homolog (340 kb in size) was missing. Since no terminal deletions or duplications other than those on chromosomes II and XVI described above were present in this isolate, we hypothesized that the 1240 kb and 610 kb chromosomes were the products of balanced translocations that did not affect the gene dosage. Assuming that this hypothesis is correct, the two chromosomes involved in the translocation must sum to 1850 kb in size. Since the band representing the W303-1A chromosome III homolog is missing on the CHEF gel (S15B Fig), this chromosome is likely involved. The size of the other chromosome must be about 1510 kb, very close to the size of chromosome IV (1530 kb). The W303-1A-derived III homolog was involved in the translocation, rather than the YJM789-derived homolog, since the 340 kb band, rather than the 315 kb band, is missing on the CHEF gel (S15B Fig). Based on the locations of Ty1 elements on chromosomes III and IV, the most likely sites for the elements involved in the translocations are located at one of the two pairs of Ty elements on III at positions 149 kb and 169 kb, and a Watson-oriented Ty on IV (*YDRWTy1-4*) at position 1096 kb. A recombination event between *YDRWTy1-4* and the Ty elements at 149 kb on III would produce translocation chromosomes of 1246 kb and 584 kb, whereas a recombination events between *YDRWTy1-4* and the Ty elements at 169 kb on III would produce translocations of 1266 kb and 604 kb. Since these two classes of translocations are not very different in size, we examined the pattern of hybridization of chromosome-specific probes to 1240 kb and 610 kb. As shown in S15B and S15C Figs, the hybridization patterns demonstrate that the translocations were formed by crossing over between *YDRWTy1-4* at 1096 kb on IV and a Watson-oriented Ty1 element at 169 kb on III. Lastly, the new junction on the IVL-IIIR translocation was confirmed by PCR analysis using primers IV-1095 S and III-170 A (S15D Fig and S2 Table). Although we characterized only one such event, the III-IV balanced translocations observed in MD741-9 demonstrates that at least one pair of translocations was generated by a reciprocal crossover between Ty elements rather than BIR.

*MD741-18*

Lastly, we identified the isolate MD741-18 that had terminal deletions on both the left and right arms of chromosome XV, and no terminal duplications. The likely mechanism to produce this pattern is an intrachromosomal crossover between the Watson-oriented Ty elements at the breakpoints. A crossover would generate the double deletion and produce a circular chromosome. PCR analysis confirmed the junction expected for a circular chromosome (S16 Fig).

**MD704 isolates**

Paired terminal deletions and duplications were also common in the MD704 isolates that were exposed to CRISPR/Cas9 for two or four hours (Dataset S2.3 in S2 Data). Although we did a detailed analysis of only a few of these isolates, we found that the events were similar to those observed for MD741.

*MD704-2h-1*

The isolate MD704-2h-1 had I-LOH events on chromosomes II, XII, and XIII, monosomy of III, a large I-DEL on chromosome IV, and T-LOH events on chromosomes VI and XI. The I-DEL on chromosome IV had Crick-oriented Ty elements at both breakpoints, indicating that the deletion was a consequence of homologous recombination between the Ty elements (Dataset S2.3 in S2 Data). The deletion on chromosome IV was confirmed by Nanopore sequencing (S17 Fig).

*MD704-4h-1*

In most isolates of MD741 or MD704, terminal deletions of one chromosome are associated with terminal duplications of another chromosome, as expected if the isolate contains one translocated chromosome. In MD704-4h-1, chromosomes III and IV have T-DELs, but no terminal duplication is present (S18A Fig). Further, since the terminal deletion of chromosome III includes the centromere, a chromosome with this deletion would be acentric and unstable. One explanation of the events in this isolate is that a homologous recombination event occurred between the Crick-oriented Ty1 element located near coordinate 169 kb of chromosome III and the Crick-oriented Ty1 element near coordinate 668 kb on chromosome IV. One of the expected translocations would contain the left arm and centromere of chromosome IV and part of the left arm of chromosome III with an expected length of 817 kb. We observed a novel chromosome of this size that hybridized to probes from chromosomes III and IV (S18B Fig). If there was a non-disjunction event resulting in loss of one pair of chromatids (containing the other translocation product and the unrecombined W303-1A-derived chromosome IV), then the cell with the unrecombined YJM789-derived copies of chromosomes IV and III in addition to the III-IV translocation would contain the observed double T-DELs on chromosomes III and IV. A possible pathway for generating the chromosome alterations is shown in S19 Fig. Two other MD704 isolates (MD704-2h-4 and MD704-4h-3-5) also had similar LOH patterns that are explicable by formation of a translocation, followed by chromosome loss.

*MD704-4h-2*

Several of the isolates had multiple regions of LOH that required invoking multiple steps in their generation. For example, the MD704-4h-2 had a complex pattern of LOH events on chromosome II, as well as a simple T-DEL on chromosome III (S20A Fig). Various segments of chromosome II were present in 0-2 copies with six transitions between these segments (S20B Fig). The chromosome rearrangements consistent with this pattern of LOH events are shown in S20C Fig, and include one unrearranged copy of chromosome III, a II-III translocation, a chromosome which has a duplication of the left arm of chromosome II and a deletion of part of the right arm, and a copy of chromosome II with portions of both W303-1A- and YJM789-derived sequences. The chromosome rearrangements involving the two chromosome II homologs, and the chromosome II-III translocation were confirmed by Nanopore sequencing in S21A and S21B Figs, respectively.

A possible pathway for generating the chromosome alterations of MD704-4h-2 is shown in S22 Fig. The steps include: 1. An I-LOH event near the centromere of chromosome II (S22A Fig), 2. A crossover on chromosome II near coordinate 328 kb (S22A Fig), 3. A recombination event between Ty elements on the right arms of chromosomes II and III (S22B Fig), and 4. A DSB on the right arm of chromosome II that is repaired by recombination with a Ty element on the left arm of chromosome II (S22C Fig).

*MD704-4h-6P*

Although most of the chromosome rearrangements of MD741 and MD704 isolates reflect intrachromosomal recombination events or translocations between two non-homologous chromosomes, we also found isolates in which portions of three different homologs were recombined into a single rearranged chromosome. In MD704-4h-6P, we observed an I-DUP of a portion of chromosome III, a T-DEL on chromosome VII, and a complicated pattern of deletions, duplications, and LOH events on chromosome XIII (S23 Fig). Subsequent analysis demonstrated that this isolate had a recombined chromosome XIII, a novel chromosome with a deletion of the right end of XIII and a duplication of the left end of XIII, and a tripartite chromosome containing the left arm and centromere of chromosome XIII adjacent to a segment derived from chromosome III adjacent to a segment containing the left arm of chromosome VII. These conclusions, largely based on microarray analysis and CHEF gels, were supported by Nanopore sequencing data showing the VII-III-XIII translocation (S24A Fig), the chromosome XIII intrachromosomal rearrangement (S24B Fig), and the chromosome XV deletion (S24C Fig).

A pathway to generate the chromosome rearrangements of MD704-4h-6P as a consequence of the recombinational repair of multiple DSBs within Ty elements is shown in S25 Fig.

Step 1. A crossover occurs on chromosome XIII with a breakpoint near 280 kb. Following chromosome segregation, the relevant cell has one recombinant product (W303-1A sequences from left telomere to position 280 kb and YJM789 sequences from 280 kb to the right telomere), and one non-recombinant YJM789-derived chromosome XIII.

Step 2. A DSB at a Crick-oriented Ty element on the right arm of XIII at 372 kb is repaired by a BIR event with a Watson-oriented Ty element on the left arm of XIII duplicating W303-1A sequences from the recombinant chromosomes. This event creates a rearrangement in which a portion of the left arm of XIII (from the left telomere to 184 kb) replaces a portion of the right arm (from 372 kb to the right telomere). The predicted size of this chromosome is about 556 kb. A novel chromosome of this size that hybridizes to a probe from the left arm of XIII was detected by CHEF gels.

Step 3. A DSB on a Watson-oriented Ty element on the right arm of VII (coordinate 536 kb) invades chromosome III at one of the Ty elements in FS2. Sequences from III between FS1 and FS2 are copied, and then the invading end is displaced and re-invades the left arm of chromosome XIII at a Watson-oriented Ty element located at 184 kb. The resulting three-chromosome translocation has sequences from the W303-1A-derived homolog of XIII from 0-184 kb, chromosome III sequences from 149-169 kb, and sequences from 0-536 kb from the W303-1A copy of chromosome VII. The predicted size of this chromosome is 740 kb, and a band of this size hybridizing to probes *PGA3* (from the left arm of XIII) and *ATE1* (from the left arm of VII) was observed. Lastly, recombination between Ty1 elements located at coordinates 664 kb and 704 kb likely produced the 40 kb internal deletion on chromosome XV.

**Analysis of delta-delta “pop-outs” and recombination breakpoints in Ty1 elements by Nanopore sequencing**

As described in the main text, in haploid cells expressing Ty1-directed CRISPR/Cas9, most (70%) of the events that deleted a *URA3* insertion within a marked Ty1 element were a consequence of delta-delta recombination. To determine whether a similar pattern was observed in diploid cells, we examined in detail the Nanopore sequencing data of the isolates MD741-6, MD741-7, MD704-2h-1, MD704-4h-2, and MD704-4h-6P. There was a total of 54 complete Ty1 elements in the diploid. For each Ty1 element (excluding elements that were involved in formation of translocations or other chromosome rearrangements), we determined whether the isolate retained the Ty1 or lost the Ty1 (leaving a solo delta). Of a total of 269 Ty1 elements examined for the five isolates, only one lost the Ty1element, and there were 8 Ty1-mediated chromosome rearrangements. We also examined by Nanopore sequencing ten isolates of a haploid strain (QL62) that expressed CRISPR/Cas9. We found nine delta-delta pop-outs and no chromosome rearrangements. Thus, the pathways of recombination used to repair DSBs in haploid and diploid cells are substantially different.

Based on microarray analysis and CHEF gel studies, we identified isolates that were likely to have hybrid Ty1 elements formed by recombination between non-allelic Ty1s. For some isolates, we used Nanopore sequencing to confirm these events. To identify the position of the recombination event, we did a BLAST comparison of the parental Ty elements that were involved in the exchange. This analysis allowed us to determine the number and location of SNPs that distinguish the two Ty1 elements. As an example, we will discuss the hybrid Ty1 formed by recombination between *YPLWTy1-1* and *YPRCTy1-2* (Fig 7D and S5 Fig). A BLAST comparison of these two elements shows that there are about 100 SNPs. From Nanopore sequencing, we had 15 “reads” that contained the intact hybrid Ty element. For every SNP, we determined whether these reads had the SNP characteristic of *YPLWTy1-1* or *YPRCTy1-2*.

**Calculation of the expected and observed frequencies of intrachromosomal and interchromosomal recombination events between Ty elements in diploid strains**

Using the information in S1 Data, we first calculated the expected numbers of intrachromosomal and interchromosomal recombination events that would produce a chromosome alteration detectable by short-read whole-genome sequencing (I-DEL, I-DUP, T-DEL, T-DUP) for each Ty1 in the genome. The expected numbers of such events for intrachromosomal and interchromosomal events were 302 and 2692, respectively. The observed numbers of intrachromosomal and interchromosomal events were 43 and 52 (S2 Data), respectively. By chi-square analysis, these numbers were very significantly different (p<0.0001). The expected proportions of intra- and interchromosomal events were 0.10 and 0.90, respectively. The observed proportions of intra- and interchromosomal events were 0.45 and 0.55, respectively.

**Supplementary references**

1. Curcio MJ, Lutz S, Lesage P. The Ty1 LTR-retrotransposon of budding yeast, *Saccharomyces cerevisiae*. Microbiol Spectr. 2015;3(2):1-35. <https://doi.org/10.1128/microbiolspec.MDNA3-0053-2014> PMID: 26104690

2. Argueso JL, Westmoreland J, Mieczkowski PA, Gawel M, Petes TD, Resnick MA. Double-strand breaks associated with repetitive DNA can reshape the genome. Proc Natl Acad Sci U S A. 2008;105(33):11845-11850. <https://doi.org/10.1073/pnas.0804529105> PMID: 18701715

3. Matheson K, Parsons L, Gammie A. Whole-genome sequence and variant analysis of W303, a widely-used strain of *Saccharomyces cerevisiae*. G3 (Bethesda). 2017;7(7):2219-2226. <https://doi.org/10.1534/g3.117.040022> PMID: 28584079

4. St Charles J, Petes TD. High-resolution mapping of spontaneous mitotic recombination hotspots on the 1.1 Mb arm of yeast chromosome IV. PLoS Genet. 2013;9(4):e1003434. <https://doi.org/10.1371/journal.pgen.1003434> PMID: 23593029

5. Lemoine FJ, Degtyareva NP, Lobachev K, Petes TD. Chromosomal translocations in yeast induced by low levels of DNA polymerase a model for chromosome fragile sites. Cell. 2005;120(5):587-598. <https://doi.org/10.1016/j.cell.2004.12.039> PMID: 15766523

6. Zheng DQ, Zhang K, Wu XC, Mieczkowski PA, Petes TD. Global analysis of genomic instability caused by DNA replication stress in *Saccharomyces cerevisiae*. Proc Natl Acad Sci U S A. 2016;113(50):8114-8121. <https://doi.org/10.1073/pnas.1618129113> PMID: 27911848

7. Al-Zain AM. Mutagenic repair outcomes of DNA double-strand breaks. : Columbia University; 2021.

8. Thomas BJ, Rothstein R. Elevated recombination rates in transcriptionally active DNA. Cell. 1989;56(4):619-630. <https://doi.org/10.1016/0092-8674(89)90584-9> PMID: 2645056

9. Goldstein AL, McCusker JH. Three new dominant drug resistance cassettes for gene disruption in *Saccharomyces cerevisiae*. Yeast. 1999;15(14):1541-1553. <https://doi.org/10.1002/(SICI)1097-0061(199910)15:14><1541::AID-YEA476>3.0.CO;2-K PMID: 10514571

10. Winston F, Chaleff DT, Valent B, Fink GR. Mutations affecting Ty-mediated expression of the *HIS4* gene of *Saccharomyces cerevisiae*. Genetics. 1984;107(2):179-197. <https://doi.org/10.1093/genetics/107.2.179> PMID: 6329902

11. Laughery MF, Hunter T, Brown A, Hoopes J, Ostbye T, Shumaker T, et al. New vectors for simple and streamlined CRISPR-Cas9 genome editing in *Saccharomyces cerevisiae*. Yeast. 2015;32(12):711-720. <https://doi.org/10.1002/yea.3098> PMID: 26305040
